# Pathways through which water, sanitation, hygiene, and nutrition interventions reduce antibiotic use in young children: a mediation analysis of a cohort nested within a cluster-randomized trial

**DOI:** 10.1016/j.eclinm.2025.103147

**Published:** 2025-03-06

**Authors:** Anna T. Nguyen, Gabby Barratt Heitmann, Andrew Mertens, Sania Ashraf, Md Ziaur Rahman, Shahjahan Ali, Mahbub Rahman, Benjamin F. Arnold, Jessica A. Grembi, Audrie Lin, Ayse Ercumen, Jade Benjamin-Chung

**Affiliations:** a Department of Epidemiology and Population Health, School of Medicine, Stanford University, Stanford, CA, USA; b Division of Epidemiology and Biostatistics, School of Public Health, University of California, Berkeley, Berkeley, CA, USA; c Environmental Interventions Unit, Health System and Population Studies Division, icddr,b, Dhaka, 1212, Bangladesh; d Department of Microbiology and Environmental Toxicology, UC Santa Cruz, Santa Cruz, CA, USA; e Infectious Disease Division, International Centre for Diarrhoeal Disease Research, Bangladesh, Dhaka, Bangladesh; f Francis I. Proctor Foundation and Department of Ophthalmology, University of California, San Francisco, San Francisco, CA, USA; g Division of Infectious Diseases and Geographic Medicine, Department of Medicine, School of Medicine, Stanford University, Stanford, CA, USA; h Department of Forestry and Environmental Resources, North Carolina State University, Raleigh, NC, USA; i Chan Zuckerberg Biohub, San Francisco, CA, USA

**Keywords:** WASH interventions, Antibiotic use, Causal mediation, Diarrhea, Acute respiratory infection, Enteric virus

## Abstract

**Background:**

Low-cost, household-level water, sanitation, and hygiene (WASH) and nutrition interventions can reduce pediatric antibiotic use, but the mechanism through which interventions reduce antibiotic use has not been investigated.

**Methods:**

We conducted a causal mediation analysis using data collected between September 2013 and October 2015 from a cohort nested within the WASH Benefits Bangladesh cluster-randomized trial (NCT01590095). Among a subsample of children within the WASH, nutrition, nutrition + WASH, and control arms (N = 1409 children; 267 clusters), we recorded caregiver-reported antibiotic use at ages 14 and 28 months and collected stool at age 14 months. Our primary outcome was any caregiver-reported antibiotic use by index children within the past 30 or 90 days measured at age 14 and 28 months. Mediators included caregiver-reported child diarrhea, acute respiratory infection (ARI), and fever; and enteric pathogen carriage in stool measured by qPCR. Both intervention-mediator and mediator-outcome models were controlled for mediator-outcome confounders.

**Findings:**

The receipt of any WASH or nutrition intervention reduced caregiver-reported antibiotic use through all pathways in the past month by 5.5 percentage points (95% CI 1.2, 9.9), from 49.5% (95% CI 45.9%, 53.0%) in the control group to 45.0% (95% CI 42.7%, 47.2%) in the pooled intervention group. When separating this effect into different pathways, we found that interventions reduced antibiotic use by 0.6 percentage points (95% CI 0.1, 1.3) through reduced diarrhea, 0.7 percentage points (95% CI 0.1, 1.5) through reduced ARI with fever, and 1.5 percentage points (95% CI 0.4, 3.0) through reduced prevalence of enteric viruses. Interventions reduced antibiotic use through any of these measured mediators by 2.1 percentage points (95% CI −0.3, 4.5).

**Interpretation:**

WASH and nutrition interventions reduced pediatric antibiotic use through the prevention of enteric and respiratory infections in a rural, low-income population. Given that many of these infections are caused by viruses or parasites, WASH and nutrition interventions may help reduce inappropriate antibiotic use in similar settings.

**Funding:**

10.13039/100000865Bill & Melinda Gates Foundation, 10.13039/100000060National Institute of Allergy and Infectious Diseases.

**Research in context**

**Evidence before this study**

We searched for primary studies and systematic reviews that investigated mediation of antibiotic use by water, sanitation and hygiene (WASH) interventions in Scopus using (TITLE-ABS-KEY ((“WASH” OR “sanitation” OR “water” OR “hygiene” OR “nutrition”) AND (“antibiot∗”) AND (“interven∗”) AND (“mediat∗” OR “indirect effect∗” OR “pathway” OR “mechanism”) AND (“use” OR “practice∗”)). We included all publications from any date until September 4, 2024. We restricted results to studies in English, focused on humans, and within medicine, agricultural and biological sciences, immunology or microbiology, or environmental science. Our search yielded 115 studies. We found no relevant research studies. Four review studies discussed the need for improved sanitation and drinking water as an AMR control strategy in LMICs. Two study protocols described longitudinal observational studies in LMICs that will explore the relationship between WASH and antibiotic resistance.

**Added value of this study**

We used causal mediation analysis to investigate mechanisms through which WASH and nutrition interventions reduced antibiotic use in young children in a community setting in rural Bangladesh. This study is rigorous because it leverages a randomized trial with high intervention adherence and includes objectively measured mediators. We found that WASH and nutrition interventions reduced antibiotic use via reduced diarrhea, ARI with fever, and enteric virus carriage. This study improves on previous studies by identifying a specific mechanism through which WASH and nutrition interventions reduced pediatric antibiotic use in an understudied setting and population.

**Implications of all the available evidence**

In a previous analysis of a randomized trial of WASH and nutrition interventions, we found that pediatric antibiotic use was lower in the intervention arms compared to control. Here, using causal mediation analysis, we identified several biologically plausible pathways through which interventions likely reduced antibiotic use. This analysis bolsters a causal interpretation that low-cost, household-level WASH and nutrition interventions can reduce pediatric antibiotic use in settings with similar infectious disease dynamics and antimicrobial access.

## Introduction

Antimicrobial resistance (AMR) is one of the top global health threats and was linked to 4.95 million deaths worldwide in 2019,^1^ with a larger burden than malaria or HIV. Deaths and antimicrobial-resistant infections are concentrated among low- and middle-income countries (LMICs),^1^ where there are high rates of antibiotic misuse due to inappropriate prescriptions for non-bacterial infections.^2^^,^^3^ Diarrhea cases of unconfirmed etiology in LMICs are frequently treated with antibiotics,^4^ despite enteric viruses being a leading cause of diarrhea among children under 24 months.^5^^,^^6^ Consumption of antibiotics that have higher potential for resistance according to the WHO grew by 165% in LMICs from 2000–2015.^7^

Though much research has investigated AMR transmission in hospital settings,^8^ a growing body of research points to a high burden of AMR in community settings in LMICs.^9^ Antibiotic-resistant pathogens can be spread through contaminated water, food, or fomites.^10^ Improved water, sanitation, and handwashing (WASH) infrastructure could reduce transmission of Antibiotic-resistant pathogens from human and zoonotic sources.^11^ Improved WASH was found to reduce the abundance of antibiotic resistance genes (ARGs) by 22% in community settings^12^ and a modeling analysis suggested that universal access to improved WASH could reduce the number of antibiotics used by 60% in LMICs.^13^ Enhanced child nutrition may also play a role in combating AMR by boosting immunity and prevent enteric and respiratory infections,^14^ which are drivers of antibiotic use and AMR.^3^^,^^15^

We previously found that low-cost, household-level WASH and nutrition interventions reduced antibiotic use in rural communities in Bangladesh by 10% and 14% within the WASH Benefits cluster-randomized trial.^16^ However, the mechanism for these reductions is poorly understood. It is possible that WASH and nutrition interventions reduce diarrhea, enteric pathogen infections, and respiratory infections, and these reductions resulted in lower antibiotic use. Among children under 2 years, WASH interventions reduced diarrhea by 31–40%,^17^ enteric virus carriage by approximately 50%,^18^ and acute respiratory infection (ARI) by 25–33%.^19^ Nutrition interventions reduced childhood diarrhea by 26%^17^ and enteric virus carriage by up to 42%.^18^ WASH and nutrition interventions may also affect antibiotic use through the promotion of healthy child immune development. Preventing chronic infections and reducing micronutrient deficiencies could prevent downstream infections that would have been treated with antibiotics.^20^^,^^21^ No prior studies have formally investigated pathways through which WASH and nutrition interventions reduce antibiotic use in LMICs.

We investigated whether reductions in pediatric antibiotic use due to WASH and nutrition interventions were mediated by reduced enteric virus carriage, diarrhea, or ARI in children under 2 years in rural Bangladesh using data from a cohort nested within the WASH Benefits Bangladesh trial.^17^ Understanding the relative importance of specific pathways may help inform whether WASH and nutrition interventions reduce necessary vs. unnecessary antibiotic use. If their effect on antibiotic use operates through infections that are likely caused by viruses, WASH and nutrition interventions may be effective at reducing inappropriate antibiotic use and subsequent threats of AMR.

## Methods

We published a pre-analysis plan at https://osf.io/ytmcr and listed deviations from this plan in Supplement 1.

### Study design & population

We conducted a causal mediation analysis using data from a prospectively measured cohort nested within the WASH Benefits Bangladesh trial. The trial enrolled 720 clusters including 5551 pregnant mothers in four districts of rural Bangladesh between May 31, 2012, and July 7, 2013.^17^ These districts were chosen because they were primarily rural, had low levels of iron and arsenic in drinking water (to allow for effective water treatment with chlorine), had no major ongoing nutrition or WASH programs, and did not include haor areas, which are shallow depressions that flood in the monsoon season.^22^ Clusters, comprising of 8 eligible women that lived close enough to be visited by a single community promotor, were randomly allocated to the following arms: water (W), hygiene (H), sanitation (S), combined water, sanitation, and hygiene (WASH), nutrition (N), combined N + WASH, and a double-sized control. Randomization was stratified by geographic block. Pregnant mothers in their first two trimesters were eligible for inclusion if they planned to live in the study village for the next two years. Only one pregnant woman was enrolled per compound, but if the woman gave birth to twins, both children were enrolled. Index children were followed for two years to assess the primary outcomes of child diarrhea and growth.

This study utilized data from the environmental enteric dysfunction (EED) substudy of WASH Benefits in which additional measurements were collected from index children in 267 clusters in the N, WASH, N + WASH, and C arms.^23^ Clusters were selected into the substudy based on logistical feasibility for the collection and transport of specimens. Clusters were equally distributed between study arms but did not adhere to the geographic matching of the parent trial. Baseline characteristics of selected households were balanced between study arms and were similar to the characteristics of households enrolled in the full trial (Table 1). The EED cohort enrolled 1531 children from 257 clusters at age 14 months and 1531 children from 267 clusters at age 28 months. All eligible children were sampled at 14- and 28-months, but some children were absent from either follow-up period for various reasons.

**Table 1:** Distribution of baseline characteristics by treatment group.

Characteristic	Any intervention (N = 1294 children)	Control (N = 422 children)
Child's sex		
Female	1142 (49.2%)	372 (50.61%)
Male	1179 (50.8%)	363 (49.39%)
Birth order		
1	786 (33.86%)	272 (37.01%)
2+	1486 (64.02%)	416 (56.6%)
Missing	49 (2.11%)	47 (6.39%)
Mother's age	24.16 (5.30)	23.45 (4.85)
Mother's height	150.30 (5.41)	150.84 (5.16)
Mother's education		
No education	334 (14.39%)	78 (10.61%)
Primary (1–5 y)	692 (29.81%)	179 (24.35%)
Secondary (>5 y)	1295 (55.79%)	478 (65.03%)
Household food insecurity		
Food secure	1626 (70.06%)	543 (73.88%)
Mildly food insecure	201 (8.66%)	62 (8.44%)
Moderately/severely food insecure	494 (21.28%)	130 (17.69%)
Number of individuals in household under age 18	1.63 (1.27)	1.56 (1.25)
Number of individuals in compound	11.41 (6.55)	10.10 (6.07)
Distance to water source	0.89 (3.94)	0.75 (1.53)
Improved roof material (tin, cement)	2289 (98.62%)	729 (99.18%)
Improved wall material (wood, brick, tin)	1530 (65.92%)	449 (61.09%)
Improved floor material (wood, concrete)	274 (11.81%)	121 (16.46%)
Household wealth index		
Wealth Q1	516 (22.23%)	100 (13.61%)
Wealth Q2	540 (23.27%)	138 (18.78%)
Wealth Q3	472 (20.34%)	185 (25.17%)
Wealth Q4	523 (22.53%)	193 (26.26%)
Missing	270 (11.63%)	119 (16.19%)

Sample sizes, child demographics, and household characteristics, in the pooled intervention vs. control arm. For categorical variables, the number of occurrences and percentages are reported. For continuous variables, the mean and standard deviation (SD) are reported. We report the distribution of these variables in individual, unpooled treatment arms in Supplemental Table S2.

Our analysis was restricted to children with non-missing antibiotic use in the follow-up rounds when they were approximately 14 months or 28 months (N = 1528) old. Antibiotic use data was available for 99.6% of children at 14 months and 99.3% of children 28 months; the distribution of missing values across specific outcomes and intervention groups is included in Table S1. Missing values for antibiotic use represent cases where a survey was administered, but the caregiver did not respond to the corresponding question. We did not complete a sample size calculation for this mediation analysis and used all available measurements from the collected data.

### Interventions

The WASH intervention included chlorine tablets for water treatment and a safe water storage vessel; double-pit latrine upgrades, child potties, and hoes for removing feces; and handwashing stations. The nutrition intervention included promotion of age-appropriate maternal and infant nutrition practices and lipid-based nutrient (LNS) supplements for children from 6 to 24 months of age. Interventions were given to participants free of charge, and consumables were restocked during trial follow-up. Intervention fidelity was high as measured by structured observations and spot checks.^24^ Nutrition interventions were a child-level interventions, while drinking water and handwashing were household-level interventions. The latrine intervention was delivered to the compound, a collection of familial households that share common outdoor space. Additional intervention details are included in Supplement 2.

### Mediators

Mediators included caregiver-reported diarrhea, ARI, ARI with fever, difficulty breathing in the prior 7 days, fever in the prior 7- or 14- days, and enteric virus carriage. Diarrhea was defined as three or more loose or watery stools within 24 h or at least one bloody stool in the prior 7 days,^25^ ARI was defined as persistent cough or panting/wheezing/difficulty breathing in the prior 7 days.^26^ Diarrhea, ARI, difficulty breathing, and fever in the prior 7-days were measured at approximately 14 and 28 months, while fever in the prior 14-days was only measured at approximately 28 months.

Stool samples were collected at age 14 months and analysed for enteropathogens via quantitative polymerase chain reaction (PCR) with a Taqman array card.^5^^,^^27^ Further details of enteropathogen data collection have been described elsewhere.^18^ The Taqman enteropathogen panel included viruses, bacteria, and protozoa. Our primary analysis assessed mediation by carriage of adenovirus 40/41, norovirus GII, and sapovirus because a prior analysis found no effects of interventions on bacteria, protozoa, or other viruses.^18^ As a secondary analysis, we included all bacteria, parasites, and viruses in the Taqman panel with a prevalence greater than 5%. We included indicators of any viral and parasite carriage but could not investigate carriage of any bacteria, since bacteria were detected in over 95% of stool samples.

To account for joint effects of mediators, we also included indicators for different combinations of mediators, including any enteric virus that is included in the Taqman panel, any enteric virus with diarrhea, ARI with fever, and any mediator (diarrhea, fever, ARI, or enteric virus).

### Outcomes

Our primary outcome was any caregiver-reported antibiotic use by index children within the past 30 or 90 days measured at age 14 and 28 months. Field staff provided caregivers with a list of commonly used antibiotics^28^ and asked them to report the index child's antibiotic use. These antibiotics included Ceftriaxone, Cefixime, Amoxycillin, Ciprofloxacin, Metronidazole, Cefuroxime, Azithromycin, Cloxacillin, Ampicillin, Flucloxacillin, Levofloxacin, Gentamicin, Cephradine, Cefepime, Erythromycin, Neomycin, and Betapen.

Here, we focused on any antibiotic use in the prior 30 days as our primary outcome, since it was more likely for diarrheal disease and acute respiratory infections (reported for the prior 7 days) to impact antibiotic use in the prior 30 days vs. the prior 90 days. While uncertainty in the temporal ordering of infections and antibiotic use is a limitation of our study, focusing on 30-day utilization makes it more reasonable that infections preceded antibiotic use. We conducted additional analyses on the secondary outcomes of any antibiotic use, multiple antibiotic use, number of episodes of antibiotic use, and number of days of antibiotic use in the 90-day recall period.

### Statistics

Our primary analysis compared a pooled intervention group (WASH, N, or N + WASH) to the control arm in an intention-to-treat analysis. We conducted secondary analyses comparing individual intervention arms to the control arm, as well as those that grouped (1) WASH and WASH + Nutrition and (2) Nutrition and WASH + Nutrition. We estimated prevalence ratios (PR) from treatment-outcome, treatment-mediator, and mediator–outcome relationships using generalized linear models with robust standard errors that account for clustering at the block level to account for the block-matched design. For continuous outcomes, we used a Gaussian family and identity link function. For categorical outcomes, we used a Poisson family and log link function with robust standard errors.^29^ We estimated prevalence differences (PD) from treatment-outcome models using g-computation with logistic regression and constructed confidence intervals with 1000 iterations of bootstrapped resampling at the block level. All models were adjusted for child age to account for differences in infection risk and antibiotic use at 14 and 28 months.

We adjusted mediator-outcome models for all possible, measured confounders: time of fecal sample/data collection (measured in 3-month periods); sex; birth order; mother's age, height and education; baseline household food insecurity; number of individuals <18 years in household; number of individuals living in compound; distance to household's primary drinking water source; and a household wealth index calculated from the first principal component of a principal components analysis of household assets. All covariates were measured at baseline except for month of measurement, child age, and child sex. Child age and sex was recorded through caregiver survey at each follow-up period.

We will use causal mediation methods to estimate the natural indirect effect of WASH and nutrition interventions on antibiotic use. The total effect of WASH and nutrition interventions on antibiotic use can be decomposed into the mediated effect (i.e., “natural indirect effect” (NIE)) or the direct effect (i.e., “natural direct effect”).^30^ The NIE is the difference in potential outcomes under the predicted values of the mediator if all children had been in the treatment arm vs. if all children had been in the control arm. In other words, the NIE estimates the effect of WASH and nutrition interventions through pathways that involve a mediator. We assumed that a NIE was only possible for a particular mediator-outcome pair if the mediator had a significant (*p*-value <0.05) or large effect (PR > 1.1 for categorical outcomes, mean difference >0.5 for continuous outcomes) on the outcome, independent from the intervention. For the filtered mediator–outcome relationships, we estimated NIEs and 95% confidence intervals using quasi-Bayesian approximations implemented by the mediation R package,^24^ in which the coefficients of the intervention-mediator and mediator-treatment models are re-drawn from random distributions. In these mediation analyses, we used intervention-mediator and mediator-outcome models that followed the same generalized linear modeling approach described above. We adjusted both models for mediator-outcome confounders that were identified in covariate screening. We assessed potential intervention-mediator interactions within mediation models. For any models in which there was a difference in NIE in the treatment vs. control greater than 1% or t-test *p*-value <0.2 when a treatment-mediator interaction term was included, we reported the NIEs estimated while holding either treatment status constant to treated (the “total” NIE) or control (the “pure” NIE) (additional details in Supplement 3).^31^

All analyses were conducted in R (version 4.1.3).

#### Assumptions of mediation analyses

A graphical representation of our assumed causal model is included in Figure S1. Because our analysis uses data from a randomized trial with baseline balance, we reasonably met the assumption that there is no unmeasured confounding of the treatment–outcome relationship. We attempted to meet the assumption of no mediator-outcome confounding through covariate adjustment, although residual unmeasured confounding is always a possibility. We performed a complete case analysis, assuming missing data are missing completely at random.

#### Sensitivity analysis

Because some children may carry enteric viruses at low levels, we performed a sensitivity analysis of mediation by enteric viruses in children whose pathogen loads reflect diarrheal etiology. We used published Ct cutoff values for adenovirus 40/41, norovirus GII and sapovirus from the MAL-ED study.^32^ We also conducted a negative control analysis using caregiver-reported bruising in the prior 7 days to detect potential mediator misclassification or residual unmeasured confounding.^33^^,^^34^

### Ethics

All study participants gave written informed consent in Bengali. This study protocol was approved for human subjects research at the icddr,b (PR-11063), University of California, Berkeley (2011-09-3652), and Stanford University (25,863).

### Role of funding source

The funders of this study had no role in the study design, data collection, data analyses, interpretation, writing of the report, or decision to submit for publication.

## Results

**Fig. 1:**
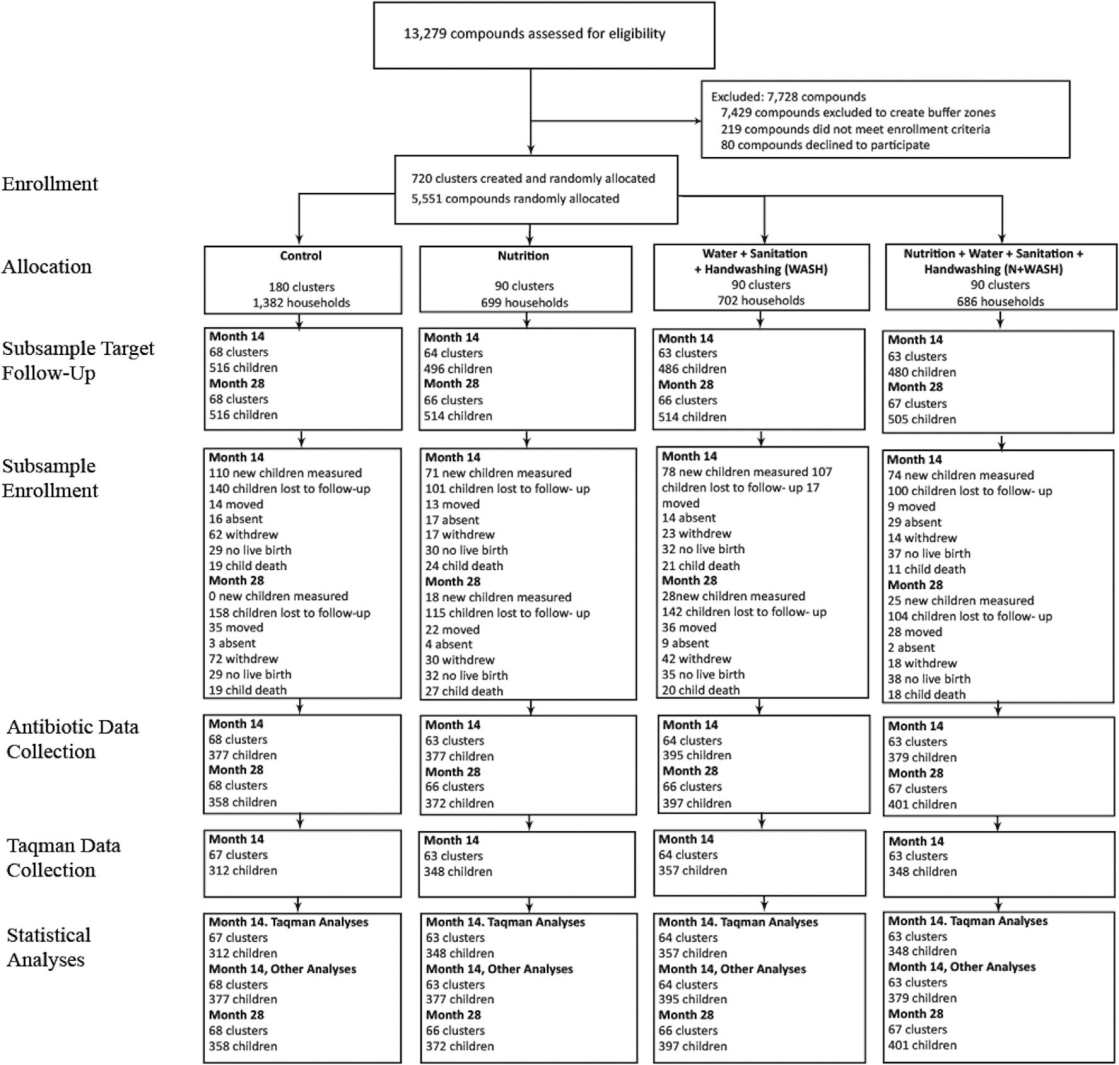
**Participant flowchart**. Flowchart of trial participants that are included in the present analysis, which was restricted to children with non-missing antibiotic use in the follow-up rounds when they were approximately 14 months or 28 months.

This analysis included 1716 children present at 14 or 28 month follow-ups (N = 3056 measurements) conducted between September 2013 and October 2015 (Fig. 1). Baseline characteristics, including child sex, mother's age, and household materials, were well-balanced at baseline between study arms (Table 1, Table S2). In all arms, over two thirds of participants were food secure, while over half of mothers had obtained a secondary education. At 14 months, 128 (5.9%) children were missing stool samples in the intervention arm, and 74 (10.7%) were missing a stool sample in the control arm.

Generally, diarrhea, ARI, and enteric virus carriage were more prevalent at 14 months compared to 28 months (Table 2, Table S3). Co-prevalence of diarrhea, ARI, and enteric virus carriage was relatively rare; <1% of children experienced diarrhea and ARI concurrently and 2% of children experienced ARI while carrying an enteric virus (Figure S2, Table S4).

**Table 2:** Prevalence of mediators and antibiotic use outcomes by treatment group at 14- and 28-months follow-up.

	Any intervention (N = 1294 children)		Control (N = 422 children)	
	14 Mos. N = 1151 measurements	28 Mos. N = 1170 measurements	14 Mos. N = 377 measurements	28 Mos N = 358 measurements
**Child characteristics**				
Month of measurement				
Feb–May	265 (23.02%)	424 (36.24%)	139 (36.87%)	114 (31.84%)
Jun–Sep	633 (55%)	293 (25.04%)	89 (23.61%)	169 (47.21%)
Oct–Jan	253 (21.98%)	453 (38.72%)	149 (39.52%)	75 (20.95%)
Child's age	8.56 (1.74)	22.84 (2.15)	9.42 (1.72)	23.74 (1.98)
**Mediators**				
Diarrhea, fever, ARI, or enteric virus	683 (64.86%)	NA	238 (76.28%)	NA
Diarrhea fever, or ARI	596 (51.78%)	319 (27.69%)	198 (52.52%)	108 (31.03%)
Diarrhea	147 (12.77%)	45 (3.91%)	67 (17.77%)	17 (4.89%)
ARI	347 (30.15%)	86 (7.47%)	119 (31.56%)	38 (10.92%)
ARI with fever	183 (15.9%)	47 (4.08%)	75 (19.89%)	20 (5.75%)
Fever (past 7 days)	368 (31.97%)	257 (22.31%)	126 (33.42%)	83 (23.85%)
Number of enteric viruses	0.38 (0.61)	NA	0.60 (0.63)	NA
Any enteric viruses	330 (31.34%)	NA	163 (52.24%)	NA
**Antibiotic outcomes**				
Any antibiotics (past month)	621 (53.95%)	419 (36.06%)	221 (58.78%)	142 (39.66%)
Any antibiotics (past 3 months)	807 (70.11%)	590 (50.43%)	283 (75.07%)	206 (57.54%)
Days of antibiotic use (past 3 months)	5.39 (5.61)	3.32 (4.47)	6.27 (5.91)	4.01 (4.99)
Episodes of antibiotic use (past 3 months)	1.06 (0.96)	0.65 (0.76)	1.23 (0.99)	0.77 (0.84)
Multiple episodes of antibiotic use (past 3 months)	295 (25.63%)	132 (11.28%)	131 (34.75%)	50 (13.97%)

Sample sizes, child characteristics, mediator prevalence, and antibiotic use prevalence, in the pooled intervention vs. control arm by follow-up period. For categorical variables, the number of occurrences and percentages are reported. For continuous variables, the mean and standard deviation (SD) are reported.

### Total effects

WASH and nutrition interventions (henceforth, “interventions”) led to a modest reduction in antibiotic use, and the strength of evidence was strongest for the effect of interventions on multiple episodes of antibiotic use in the past 3 months (Table 3, Figure S3, Table S5). Interventions reduced the prevalence of any antibiotic use in the past month from 50% in the control arm to 45% in the pooled intervention group (PD −5.5, 95% CI −1.2, −9.9). We observed similar effects of the intervention on any antibiotic use in the past 3 months (PD −7.2, 95% CI −1.7, −12.4). Interventions reduced the prevalence of multiple episodes of antibiotic use in the past 3 months by 7 percentage points, the number of days of antibiotic use by 0.93 days, and the number of episodes by 0.18 episodes. Overall, effect sizes were similar for individual WASH and nutrition interventions, as well as the WASH and WASH + Nutrition group and the Nutrition and WASH + nutrition group.

**Table 3:** Total effects of any WASH or nutrition intervention on antibiotic use.

Outcome	Prevalence/mean difference	Prevalence ratio
Any antibiotics (past month)	−0.055 (−0.099, −0.012)	0.890 (0.813, 0.974)
Any antibiotics (past 3 months)	−0.072 (−0.124, −0.017)	0.892 (0.822, 0.967)
Days of antibiotic use (past 3 months)	−0.929 (−1.405, −0.453)	–
Episodes of antibiotic use (past 3 months)	−0.175 (−0.257, −0.092)	–
Multiple episodes of antibiotic use (past 3 months)	−0.071 (−0.105, −0.038)	0.719 (0.623, 0.829)

Point estimates and 95% confidence intervals (CIs) for the total effect of any WASH or nutrition intervention on antibiotic use outcomes. For categorical outcomes (any antibiotic use, multiple episodes of antibiotic use), the prevalence difference and prevalence ratio are reported. For continuous outcomes (days of antibiotic use, episodes of antibiotic use), only the mean difference is reported.

### Indirect effects through any measured mediator

**Fig. 2:**
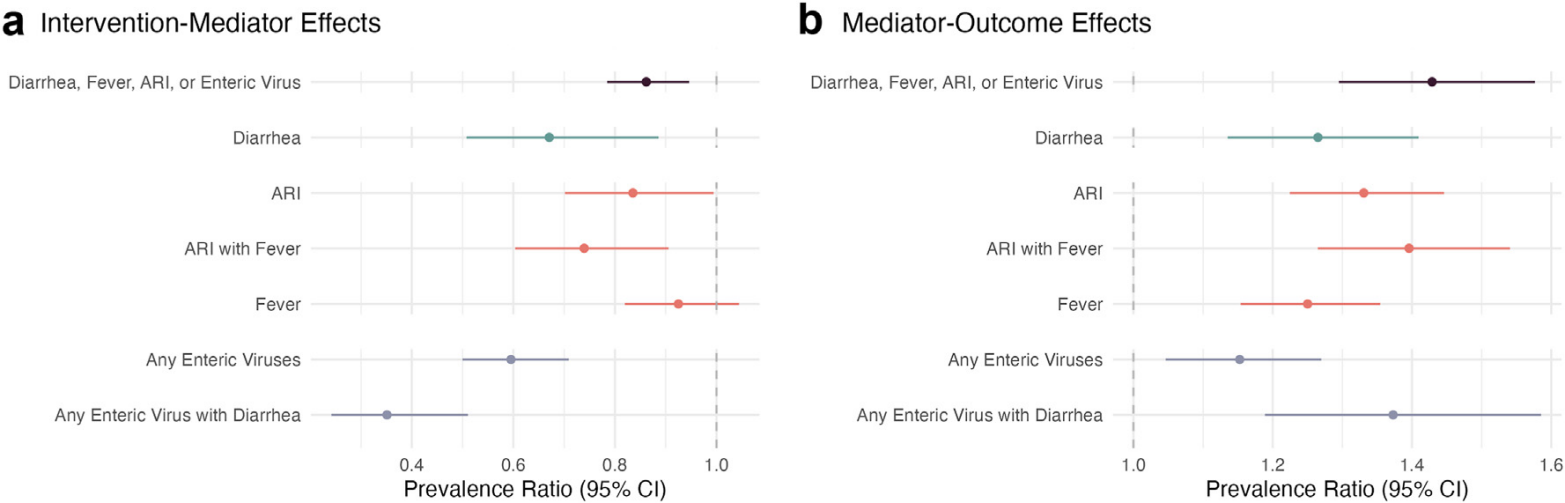
**Effects of any WASH and nutrition intervention on mediators, and effects of mediators on caregiver-reported antibiotic use**. Prevalence ratios and 95% confidence intervals for (a) effects of any water, sanitation, handwashing (WASH) and nutrition intervention on mediators and (b) mediators on any antibiotic use in the past month. Diarrhea, Acute Respiratory Infection (ARI), ARI with Fever, and Fever are all reported by a caregiver under a 7-day lookback period at 14 and 28 months. Any Enteric Virus is the presence of adenovirus 40/41, norovirus GI, norovirus GII, sapovirus, rotavirus, or astrovirus in stool collected at 14 months, and Any Enteric Virus with Diarrhea is the presence of any enteric virus with caregiver reported diarrhea in the prior 7 days at 14 months.

**Fig. 3:**
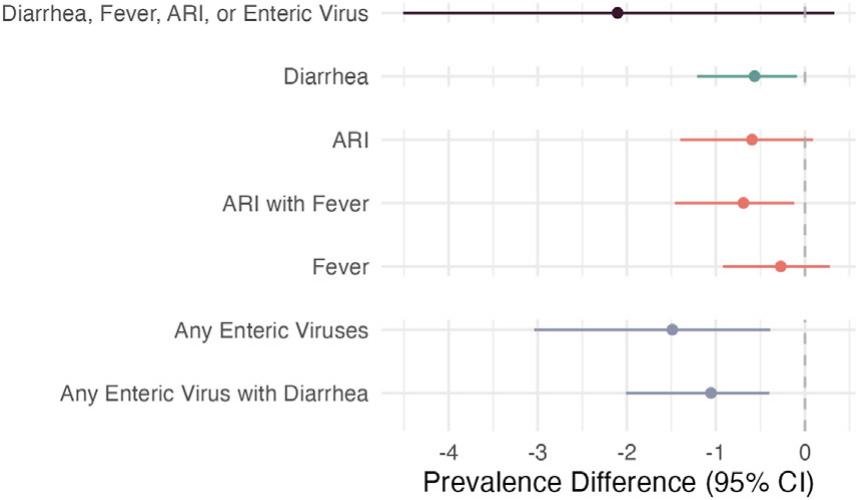
**Indirect effects of WASH and Nutrition interventions on caregiver-reported antibiotic use**. Natural indirect effects and 95% confidence intervals of water, sanitation, handwashing (WASH) and nutrition interventions on caregiver-reports of any antibiotic use in the past month (total effect = 5.5 percentage points reduction, 95% CI 1.2, 9.9). Diarrhea, Acute Respiratory Infection (ARI), ARI with Fever, and Fever are all reported by a caregiver under a 7-day lookback period at 14 and 28 months. Any Enteric Virus is the presence of adenovirus 40/41, norovirus GI, norovirus GII, sapovirus, rotavirus, or astrovirus in stool collected at 14 months, and Any Enteric Virus with Diarrhea is the presence of any enteric virus with caregiver reported diarrhea in the prior 7 days at 14 months.

Next, we assessed whether effects of interventions on antibiotic use occurred through changes in the prevalence of any of the mediators measured in our study (diarrhea, ARI, fever, or enteric virus carriage). Interventions reduced the prevalence of any mediator measured in this study by 14% (95% CI 5%, 21%), and the prevalence of any measured mediator was associated with 43% (95% CI 30%, 58%) higher antibiotic use in the past month (Fig. 2, Figures S4 and S5, Tables S6 and S7). Interventions reduced antibiotic use in the past month through any measured mediator (diarrhea, ARI, fever, or enteric virus carriage) by 2.1 percentage points (95% CI −0.3, 4.5%; Fig. 3, Figure S6, Table S8), which is about 38% of the total intervention effect. This translates to an approximate reduction of 21 cases of antibiotic use per 1000 children through intervention effects on mediators.

### Mediation through acute respiratory infections

Interventions reduced the prevalence of ARI (PR 0.84, 95% CI 0.70, 0.99), which was associated with increased antibiotic use in the prior month (PR 1.33, 95% CI 1.23, 1.45). There were slightly stronger associations between interventions, antibiotic use, and specific symptoms of ARI such as difficulty breathing and fever. In particular, we found that interventions reduced the prevalence of ARI with fever by 26% (95% CI 9%, 40%; control prevalence = 13%), and there was weak evidence of a 29% reduction in the prevalence of difficulty breathing (95% CI −10%, 55%, control prevalence 4%). Similarly, ARI with fever and difficulty breathing were associated with 40–50% higher antibiotic use in the past month (Fig. 2, Figures S4 and S5, Tables S6 and S7).

We found little evidence that ARI, with or without fever, mediated the effect of interventions on antibiotic use (Fig. 3, Figure S6, Table S8). We estimated that 5–11% of the total effect of interventions on antibiotic use in the prior month was mediated through a factor related to ARI, corresponding to an absolute reduction of 0.3–0.7 percentage points. Precision was low for all estimates and most confidence intervals contained the null. There was some evidence of intervention-mediator interactions for ARI, ARI with fever and difficulty breathing, where the total NIE was stronger than the pure NIE for any antibiotic use (Figure S7, Table S9).

### Mediation through diarrhea and enteric pathogen carriage

Interventions reduced caregiver-reported diarrhea prevalence by 33% (95% CI 11%, 49%; control prevalence = 11.6%), and diarrhea was associated with 27% higher antibiotic use in the prior month (95% CI 14%, 41%) (Fig. 2, Figures S4 and S5, Tables S6 and S7). In our mediation analysis, we found that interventions reduced antibiotic use by 0.6 percentage points (95% CI 0.1%, 1.2%) through the prevention of diarrhea (Fig. 3, Figure S6, Table S8).

Carriage of enteric viruses was reduced by the interventions (PR 0.60 95% CI 0.50, 0.71) and associated with increased antibiotic use in the past 30 days (PR 1.15, 95% CI 1.05, 1.2; Figures S8 and S9, Tables S7 and S8). We estimated that interventions reduced antibiotic use in the prior month by 1.5 percentage points (95% CI 0.4%, 3%) via the carriage of any enteric virus. There was weak evidence that Sapovirus had a larger effect than other enteric viruses, but the precision of estimates for individual species was low (Figure S10, Table S9). We found no effects of interventions on bacteria or parasite carriage, and we did not find evidence of associations with bacteria or parasite carriage.

We observed stronger mediation through diarrhea with enteric virus carriage than with diarrhea alone. Interventions reduced diarrhea with enteric virus carriage by 65% (95% CI 49%, 76%), and diarrhea with enteric virus carriage was associated with 37% (95% CI 19%, 59%) higher antibiotic use in the prior month. Intervention effects on diarrhea with enteric virus carriage reduced antibiotic use by 1.1 (95% CI 0.4, 2) percentage points, accounting for about 20% of the total intervention effect. Effect sizes for mediation through diarrhea with enteric virus carriage were similar though slightly weaker for individual nutrition interventions compared to WASH interventions. We found evidence of intervention-mediator interactions for diarrhea with enteric virus carriage for the number of days and episodes of antibiotic use in the prior 3 months, where the pure NIE was stronger than the total NIE. These interactions were only present in the Nutrition and Nutrition + WASH intervention groups (Figure S7, Table S9).

### Sensitivity analyses

In analyses using prevalence of enteric viruses with pathogen loads likely to reflect diarrheal etiology, treatment-mediator and mediator-outcome associations were similar but less estimated with lower precision (Figures S11–S13, Tables S13–S15). There was no evidence of mediation by any indicator of symptomatic enteric virus carriage.

### Negative control

When repeating mediation analyses using bruising prevalence as a negative control for caregiver-reported mediators (e.g., diarrhea, ARI), all 95% confidence intervals for indirect effect estimates contained the null, suggesting that mediator misclassification did not have a large impact on our findings (Figure S14, Table S16).

## Discussion

In this mediation analysis of a cohort nested within a cluster-randomized trial, effects of low-cost, household-level WASH and nutrition interventions on reduced pediatric antibiotic use occurred via reduced diarrhea and enteric viruses. The strongest mediators were enteric virus carriage and diarrhea with enteric virus carriage, suggesting that interventions effectively reduced antibiotic use by preventing viral enteric infections. Overall, mediation patterns were similar for individual WASH and nutrition interventions, with slightly stronger mediated effects for the WASH and WASH + Nutrition interventions.

About a third of the effect of WASH and nutrition interventions on reduced pediatric antibiotic use was mediated by reduced enteric virus carriage, both with and without diarrhea symptoms. Enteric virus carriage that is not accompanied by caregiver-reported diarrhea might reflect infections that caused non-diarrheal symptoms (ex: vomiting) or diarrhea that occurred prior to the 7-day recall period. Prior studies have found that viruses are responsible for a large fraction of clinically-attended childhood diarrhea treated with antibiotics in LMICs,^35^ yet diarrhea is frequently treated with antibiotics.^4^ The ratio of appropriately to inappropriately treated diarrhea cases in LMICs is estimated to be 12.6.^4^ These numbers may be larger in community settings where antibiotics are purchased without clinician guidance. By preventing infections caused by enteric viruses, WASH and nutrition interventions may be an effective strategy to reduce inappropriate antibiotic use where unimproved WASH contributes to high burdens of diarrheal disease.

There was weak evidence that interventions reduced antibiotic use by reducing acute respiratory infections. This is likely because WASH and nutrition intervention effects on ARI were relatively modest, despite large associations with ARI and antibiotic use. While we did not have data on the etiology of ARI in this study, ARI in young children in LMICs are more often of viral etiology than bacterial etiology.^36^, ^37^, ^38^, ^39^ Yet, prior studies in Bangladesh and other LMICs have found that 37%–80% of children under 5 with respiratory symptoms were treated with antibiotics.^40^, ^41^, ^42^ While we did not observed significant indirect effects of WASH and Nutrition interventions on ARI, our findings highlight an opportunity for alternative interventions to reduce inappropriate antibiotic use by preventing viral ARIs, such as influenza or coronavirus.

The mediators measured in this study accounted for 38% of the total effect of interventions on antibiotic use. It is possible that there are other important mediators that we did not measure. WASH interventions may reduce skin or eye infections, and such reductions could have resulted in lower antibiotic use. For the nutrition intervention, a key mediating pathway may be improved immune function, however this was beyond the scope of our analysis. A prior analysis found that WASH and nutrition interventions enhanced immunoprotection and immunoregulation and suppressed the immunopathologic response.^20^ Given that nutrition is likely an upstream determinant of immunity and infection, studies have proposed nutrition interventions as a critical tool for reducing AMR in LMICs.^21^ Understanding the immunologic pathways through which nutrition interventions mediate effects on antibiotic use is an important area for future research.

Our findings of reduced antibiotic use do not necessarily reflect changes in AMR carriage. For example, one study in urban Bangladesh found that community-scale water chlorination reduced child antibiotic use by 7% but did not result in reduced prevalence of AMR genes in child stool.^43^^,^^44^ While we are not aware of any prior studies that have measured effects of lipid nutrient supplementation on AMR, prior studies have found that dietary diversity was associated with reduced indicators of AMR.^15^ Future studies of WASH and nutrition interventions would benefit from inclusion of antibiotic resistance carriage as an outcome.

Strengths of this study include the use of data from a randomized trial with high intervention uptake and minimized measured and unmeasured confounding of the intervention–mediator relationships. There were several limitations. First, we relied upon caregiver-reported diarrhea, respiratory infection, and antibiotic use, which may be subject to courtesy and recall bias. Caregivers may also have given children medications that they are unaware are antibiotics, which could be of concern if antibiotics are obtained outside of a clinical setting. However, our negative control analysis suggests that there was minimal outcome misclassification. Additionally, prior work has shown that there is strong concordance between medical records and caregiver report of antibiotic use in low- and middle-income countries.^45^ Second, the recall period was 1–3 months for antibiotic use and 7 days for diarrhea and ARI, which were measured concurrently; it is possible that in some cases, antibiotic use preceded diarrhea or ARI. Our results were similar using 1 vs. 3 month recall periods, suggesting that lack of temporal ordering did not have a large influence, but we cannot completely rule it out. Third, we did not consider multiple mediators within a single model; it is possible that our approach did not fully capture complex relationships between mediators. However, joint infections were relatively rare, and we did include an analysis using an indicator for any measured mediator. Fourth, it is possible that our mediator-outcome models were subject to residual confounding or that missing confounder values impacted our effect estimates. We conducted a complete case analysis but had to excluded 12% of our data in mediation analyses where measurements for at least one confounder was missing. However, since most confounders were assessed at baseline, we expect missingness to be random across intervention groups, and it is not possible that future infections or antibiotic use impacted missingness. Thus, the exclusion of children with missing cofounder data is unlikely to have biased our estimates, but they may have decreased precision in mediation analyses. Similarly, the selection of clusters into the substudy may have introduced bias. In particular, political unrest associated with the 2014 general election in Bangladesh may have impacted inclusion in the 14-month follow-up. However, the baseline characteristics of selected households at both follow-up periods were balanced between all study arms and were similar to the characteristics of households enrolled in the full trial.^23^ This suggests that there was little evidence of measured confounding. Finally, our results may not generalize to other populations with differing climatic conditions, pathogen transmission patterns, antibiotic availability, animal ownership, and population density.

In conclusion, our findings shed light on the mechanism through which WASH and nutrition interventions reduce antibiotic use and support a causal interpretation for effects on antibiotic use. We provide evidence that the effect of these interventions on pediatric antibiotic use was mediated through the reduction of enteric and respiratory infections that are likely caused by viruses. Taken together with prior studies, our findings support the use of WASH and nutrition interventions to reduce inappropriate antibiotic use and AMR, particularly in regions with similar burdens of viral enteric infections.

## Contributors

Conceptualization: JBC, AE.

Methodology: JBC, ATN, AE, JG, AL, AM, BFA.

Software: ATN, GBH.

Validation: GBH.

Formal analysis: ATN.

Investigation: S Ashraf, S Ali, JG, AL, MR.

Data curation: ZR, S Ali, JG, AL, AE, ATN.

Writing–original draft: ATN, GBH, JBC.

Writing–reviewing & editing: All authors.

Visualization: ATN, GBH.

Supervision: JBC.

Project administration: AL, JG, S Ashraf, ZR, S Ali, MR.

Funding acquisition: BFA.

All authors have read and approved the final version of the manuscript. ATN, GBH, and JBC have directed accessed and verified the underlying data reported in the manuscript.

## Data sharing statement

We have published replication data and software on Open Science Framework (https://osf.io/ytmcr).

## Declaration of interests

BFA is a Data Safety Monitoring Board member for a National Institute of Allergy and Infectious Diseases award (R01AI184756, Multi-component chlorination intervention to reduce neonatal infections in rural health facilities). The other authors declare no competing interests.

## References

[1] Murray C.J.L., Ikuta K.S., Sharara F. (2022). Global burden of bacterial antimicrobial resistance in 2019: a systematic analysis. Lancet.

[2] Sulis G., Adam P., Nafade V. (2020). Antibiotic prescription practices in primary care in low- and middle-income countries: a systematic review and meta-analysis. PLoS Med.

[3] Ocan M., Obuku E.A., Bwanga F. (2015). Household antimicrobial self-medication: a systematic review and meta-analysis of the burden, risk factors and outcomes in developing countries. BMC Public Health.

[4] Lewnard J.A., Rogawski McQuade E.T., Platts-Mills J.A., Kotloff K.L., Laxminarayan R. (2020). Incidence and etiology of clinically-attended, antibiotic-treated diarrhea among children under five years of age in low- and middle-income countries: evidence from the Global Enteric Multicenter Study. PLoS Negl Trop Dis.

[5] Liu J., Platts-Mills J.A., Juma J. (2016). Use of quantitative molecular diagnostic methods to identify causes of diarrhoea in children: a reanalysis of the GEMS case-control study. Lancet.

[6] Platts-Mills J.A., Liu J., Rogawski E.T. (2018). Use of quantitative molecular diagnostic methods to assess the aetiology, burden, and clinical characteristics of diarrhoea in children in low-resource settings: a reanalysis of the MAL-ED cohort study. Lancet Glob Health.

[7] Klein E.Y., Milkowska-Shibata M., Tseng K.K. (2021). Assessment of WHO antibiotic consumption and access targets in 76 countries, 2000-15: an analysis of pharmaceutical sales data. Lancet Infect Dis.

[8] Saharman Y.R., Karuniawati A., Severin J.A., Verbrugh H.A. (2021). Infections and antimicrobial resistance in intensive care units in lower-middle income countries: a scoping review. Antimicrob Resist Infect Control.

[9] Ingle D.J., Levine M.M., Kotloff K.L., Holt K.E., Robins-Browne R.M. (2018). Dynamics of antimicrobial resistance in intestinal Escherichia coli from children in community settings in South Asia and sub-Saharan Africa. Nat Microbiol.

[10] Ayukekbong J.A., Ntemgwa M., Atabe A.N. (2017). The threat of antimicrobial resistance in developing countries: causes and control strategies. Antimicrob Resist Infect Control.

[11] Swarthout J.M., Chan E.M.G., Garcia D., Nadimpalli M.L., Pickering A.J. (2022). Human colonization with antibiotic-resistant bacteria from nonoccupational exposure to domesticated animals in low- and middle-income countries: a critical review. Environ Sci Technol.

[12] Fuhrmeister E.R., Harvey A.P., Nadimpalli M.L. (2023). Evaluating the relationship between community water and sanitation access and the global burden of antibiotic resistance: an ecological study. Lancet Microbe.

[13] O'Neill J. (2016). https://amr-review.org/sites/default/files/160525_Final%20paper_with%20cover.pdf.

[14] Siddiqui F.J., Belayneh G., Bhutta Z.A., Humphries D.L., Scott M.E., Vermund S.H. (2021). Nutrition and infectious diseases: shifting the clinical paradigm.

[15] Oliver A., Xue Z., Villanueva Y.T. (2022). Association of diet and antimicrobial resistance in healthy U.S. Adults. mBio.

[16] Ercumen A., Mertens A.N., Butzin-Dozier Z. (2025). Water, sanitation, handwashing, and nutritional interventions can reduce child antibiotic use: evidence from Bangladesh and Kenya. Nat Commun.

[17] Luby S.P., Rahman M., Arnold B.F. (2018). Effects of water quality, sanitation, handwashing, and nutritional interventions on diarrhoea and child growth in rural Bangladesh: a cluster randomised controlled trial. Lancet Glob Health.

[18] Grembi J.A., Lin A., Karim M.A. (2023). Effect of water, sanitation, handwashing, and nutrition interventions on enteropathogens in children 14 Months old: a cluster-randomized controlled trial in rural Bangladesh. J Infect Dis.

[19] Ashraf S., Islam M., Unicomb L. (2020). Effect of improved water quality, sanitation, hygiene and nutrition interventions on respiratory illness in young children in rural Bangladesh: a multi-arm cluster-randomized controlled trial. Am J Trop Med Hyg.

[20] Lin A., Mertens A.N., Tan S. (2021). Effects of drinking water, sanitation, handwashing, and nutritional interventions on immune status in young children: a cluster-randomized controlled trial in rural Bangladesh. Clin Infect Dis.

[21] Unger S.A., Mark H., Pagliari C. (2019). Nutrition: the missing link in the battle against microbial resistance?. J Glob Health.

[22] Arnold B.F., Null C., Luby S.P. (2013). Cluster-randomised controlled trials of individual and combined water, sanitation, hygiene and nutritional interventions in rural Bangladesh and Kenya: the WASH Benefits study design and rationale. BMJ Open.

[23] Lin A., Ali S., Arnold B.F. (2020). Effects of water, sanitation, handwashing, and nutritional interventions on environmental enteric dysfunction in young children: a cluster-randomized, controlled trial in rural Bangladesh. Clin Infect Dis.

[24] Parvez S.M., Azad R., Rahman M. (2018). Achieving optimal technology and behavioral uptake of single and combined interventions of water, sanitation hygiene and nutrition, in an efficacy trial (WASH benefits) in rural Bangladesh. Trials.

[25] Baqui A.H., Black R.E., Yunus M., Hoque A.R., Chowdhury H.R., Sack R.B. (1991). Methodological issues in diarrhoeal diseases epidemiology: definition of diarrhoeal episodes. Int J Epidemiol.

[26] Feikin D.R., Olack B., Bigogo G.M. (2011). The burden of common infectious disease syndromes at the clinic and household level from population-based surveillance in rural and urban Kenya. PLoS One.

[27] Liu J., Gratz J., Amour C. (2016). Optimization of quantitative PCR methods for enteropathogen detection. PLoS One.

[28] Ercumen A., Mertens A.N., Butzin-Dozier Z. (2024). Can drinking water, sanitation, handwashing, and nutritional interventions reduce antibiotic use in young children?. medRxiv.

[29] Zou G. (2004). A modified Poisson regression approach to prospective studies with binary data. Am J Epidemiol.

[30] Pearl J. (2012). The causal mediation formula--a guide to the assessment of pathways and mechanisms. Prev Sci.

[31] VanderWeele T.J. (2013). A three-way decomposition of a total effect into direct, indirect, and interactive effects. Epidemiology.

[32] Garcia Quesada M., Platts-Mills J.A., Liu J., Houpt E.R., Rogawski McQuade (2024). Leveraging data from a longitudinal birth cohort to improve attribution of diarrhea etiology among children in low-resource settings. J Infect Dis.

[33] Arnold B.F., Ercumen A., Benjamin-Chung J., Colford J.M. (2016). Brief report: negative controls to detect selection bias and measurement bias in epidemiologic studies. Epidemiology.

[34] Lipsitch M., Tchetgen Tchetgen E., Cohen T. (2010). Negative controls: a tool for detecting confounding and bias in observational studies. Epidemiology.

[35] Kotloff K.L., Nataro J.P., Blackwelder W.C. (2013). Burden and aetiology of diarrhoeal disease in infants and young children in developing countries (the Global Enteric Multicenter Study, GEMS): a prospective, case-control study. Lancet.

[36] Berman S. (1991). Epidemiology of acute respiratory infections in children of developing countries. Rev Infect Dis.

[37] Assane D., Makhtar C., Abdoulaye D. (2018). Viral and bacterial etiologies of acute respiratory infections among children under 5 Years in Senegal. Microbiol Insights.

[38] Bezerra P.G.M., Britto M.C.A., Correia J.B. (2011). Viral and atypical bacterial detection in acute respiratory infection in children under five years. PLoS One.

[39] Hoffmann J., Rabezanahary H., Randriamarotia M. (2012). Viral and atypical bacterial etiology of acute respiratory infections in children under 5 Years old living in a rural tropical area of Madagascar. PLoS One.

[40] Levine G.A., Bielicki J., Fink G. (2022). Cumulative antibiotic exposure in the first 5 Years of life: estimates for 45 low- and middle-income countries from demographic and health survey data. Clin Infect Dis.

[41] Hassan M.Z., Monjur M.R., Biswas M.A.A.J. (2021). Antibiotic use for acute respiratory infections among under-5 children in Bangladesh: a population-based survey. BMJ Glob Health.

[42] Fink G., D'Acremont V., Leslie H.H., Cohen J. (2020). Antibiotic exposure among children younger than 5 years in low-income and middle-income countries: a cross-sectional study of nationally representative facility-based and household-based surveys. Lancet Infect Dis.

[43] Pickering A.J., Crider Y., Sultana S. (2019). Effect of in-line drinking water chlorination at the point of collection on child diarrhoea in urban Bangladesh: a double-blind, cluster-randomised controlled trial. Lancet Global Health.

[44] Nadimpalli M.L., Lanza V.F., Montealegre M.C. (2022). Drinking water chlorination has minor effects on the intestinal flora and resistomes of Bangladeshi children. Nat Microbiol.

[45] Rogawski E.T., Platts-Mills J.A., Seidman J.C. (2017). Use of antibiotics in children younger than two years in eight countries: a prospective cohort study. Bull World Health Organ.

