# Supplementary material for: Pathways through which water, sanitation, hygiene, and nutrition interventions reduce antibiotic use in young children: a mediation analysis of a cohort nested within a cluster-randomized trial
Source: eClinicalMedicine. 2025 Mar 6;82:103147. doi: 10.1016/j.eclinm.2025.103147 (PMC11928822; doi:10.1016/j.eclinm.2025.103147)
Supplement: Supplement and Figures [file mmc1.docx]

**Supplemental Materials**

*Pathways through which water, sanitation, hygiene, and nutrition interventions reduce antibiotic use in young children: a mediation analysis of a cluster-randomized trial*

[Supplement 1: Deviations from Pre-Analysis Plan 3](#_Toc190264040)

[Supplement 2. Additional details about study interventions 4](#_Toc190264041)

[Supplement 3. Additional details about mediation analysis 4](#_Toc190264042)

[Figure S1: Directed Acyclic Graph of Assumed Causal Model 5](#_Toc190264043)

[Figure S2: Joint Prevalence of Mediators 6](#_Toc190264044)

[Figure S3: Intervention-Outcome Effects, All Intervention Groups 7](#_Toc190264045)

[Figure S4: Intervention-Mediator Effects, All Intervention Groups and Mediators 8](#_Toc190264046)

[Figure S5: Mediator-Outcome Effects, All Intervention Groups and Mediators 9](#_Toc190264047)

[Figure S6: Mediated Effects, All Intervention Groups and Mediators 10](#_Toc190264048)

[Figure S7: Mediated Effects, with Significant Intervention-Mediator Interactions 11](#_Toc190264049)

[Figure S8: Intervention-Mediator Effects, Pooled Intervention Group and Enteric Pathogen Carriage 12](#_Toc190264050)

[Figure S9: Intervention-Mediator Effects, Pooled Intervention Group and Enteric Pathogen Carriage 13](#_Toc190264051)

[Figure S10: Mediated Effects, Pooled Intervention Group and Enteric Pathogen Carriage 14](#_Toc190264052)

[Figure S11: Intervention-Mediator Effects, for Enteric Viruses with Diarrheal Etiology 15](#_Toc190264053)

[Figure S12: Mediator-Outcome Effects, for Enteric Viruses with Diarrheal Etiology 16](#_Toc190264054)

[Figure S13: Mediated Effects, for Enteric Viruses with Diarrheal Etiology 17](#_Toc190264055)

[Figure S14: Mediated Effects, Negative Control 18](#_Toc190264056)

[Table S1: Distribution of missing antibiotic use data across arms. 19](#_Toc190264057)

[Table S2: Distribution of Baseline Characteristics, Mediators, and Antibiotic Use Outcomes, All Intervention Groups 20](#_Toc190264058)

[Table S3: Distribution of Baseline Characteristics, Mediators, and Antibiotic Use Outcomes, By Intervention Arm and Follow-Up Period 22](#_Toc190264059)

[Table S4: Joint Prevalence of Mediators 24](#_Toc190264060)

[Table S5: Intervention-Outcome Effects 25](#_Toc190264061)

[Table S6: Intervention-Mediator Effects 27](#_Toc190264062)

[Table S7: Mediator-Outcome Effects 30](#_Toc190264063)

[Table S8: Mediated Effects 32](#_Toc190264064)

[Table S9: Mediated Effects, with Intervention-Mediator Interactions 35](#_Toc190264065)

[Table S10: Intervention-Mediator Effects, Pooled Intervention Group and Enteric Pathogen Carriage 38](#_Toc190264066)

[Table S11: Mediator-Outcome Effects, Pooled Intervention Group and Enteric Pathogen Etiology 39](#_Toc190264067)

[Table S12: Mediated Effects, Pooled Intervention Group and Enteric Pathogen Carriage 42](#_Toc190264068)

[Table S13: Intervention-Mediator Effects, for Enteric Viruses with Pathogen Loads Reflecting Diarrheal Etiology 45](#_Toc190264069)

[Table S14: Mediator-Outcome Effects, for Enteric Viruses with Pathogen Loads Reflecting Diarrheal Etiology 46](#_Toc190264070)

[Table S15: Mediated Effects, for Enteric Viruses with Pathogen Loads Reflecting Diarrheal Etiology 47](#_Toc190264071)

[Table S16: Mediated Effects, Negative Control 48](#_Toc190264072)

# Supplement 1: Deviations from Pre-Analysis Plan

Prior to this study, we published a pre-analysis plan at <https://osf.io/ytmcr>. Any deviations that we made to this plan are listed below.

1. We pre-specified the inclusion of Norovirus as a mediator. In the trial, both Norovirus GI and GII were measured, but Norovirus GII was the more prevalent. Additionally, the viral load cutoff for diarrheal etiology was validated for Norovirus GII, but not Norovirus GI, in the MAL-ED study. As a result, we chose to focus on Norovirus GII in this analysis, but Norovirus GI is still included in the composite “Any Enteric Virus” mediator.
2. We did not pre-specify the inclusion of the composite mediators that captured (1) diarrhea, ARI, fever, or enteric virus infection at 14 months or (2) diarrhea, ARI, or fever at 14 and 28 months.
3. We estimated the total effect of the intervention on the outcome on the absolute scale using g-computation with logistic models, but these methods were not pre-specified. The addition of these prevalence difference estimates allowed us to present total and mediated effects on the same scale to assist with interpretation.
4. We pre-specified that age would be included as a confounder if it was associated with the outcome, as indicated by a likelihood ratio test p-value < 0.2. However, we chose to adjust all models for age, regardless of the magnitude of the size of the likelihood ratio test p-value, to account for repeated measurements taken for each child at 14 vs 28 months old.
5. We pre-specified the use of log-binomial models when the response variable was categorical, and the use of modified Poisson models only if the log-binomial failed to converge. However, we found that the log-binomial models failed to converge in most cases, so we defaulted to modified Poisson models in all cases in which the response variable was categorical.
6. We pre-specified the inclusion of models with intervention-mediator interactions if the interactions had a large influence on the magnitude of direct and mediated effect estimates. However, we did not pre-specify the criteria that would be used to assess the significance of interaction terms. We chose to include interaction terms if the estimated average causal mediated effect (ACME) was more than 1% different in the intervention vs control groups, or if a t-test to assess if the difference in ACME between arms was not 0 yielded a p-value less than or equal to 0.2.

In response to peer reviewer comments, we made the following changes to our analyses:

1. We pre-specified the inclusion of only Adenovirus 40/41, Norovirus GII, and Sapovirus, but have included all bacteria, parasites, and viruses that are measured in the Taqman panel.
2. We pre-specified the use of a likelihood ratio test for confounder selection, but have adjusted for all confounders in models to estimate mediator-outcome effects and mediated effects
3. We pre-specified the inclusion of only mediator-outcome relationships that were significant based on a p-value cutoff of 0.05 in our mediation analyses. We have modified this criteria to include mediator-outcome pairs that were significant based on a p-value cutoff of 0.05 OR had a large effect size (PR > 1.1 for categorical outcomes, mean difference > 0.5 for continuous outcomes).

# Supplement 2. Additional details about study interventions

Interventions were delivered close to the time of index child births. The WASH intervention included chlorine tablets for water treatment and a safe water storage vessel; double-pit latrine upgrades for all latrines in the compound, child potties, and hoes for removing feces; and handwashing stations, which included a soapy water bottle and water for rinsing near kitchens and latrines. The nutrition intervention included promotion of age-appropriate maternal and infant nutrition practices and lipid-based nutrient (LNS) supplements for children from 6-24 months of age. Additional details about the interventions are described elsewhere.^1^ Interventions were given to participants free of charge, and consumables were restocked during trial follow-up. Local promoters visited intervention study compounds to promote intervention uptake weekly in the first 6 months of the trial and once every 2 weeks thereafter. Intervention fidelity was high in the trial as indicated through structured observations and spot checks, where adherence to most interventions exceeded 90%.^2^

1. Luby SP, Rahman M, Arnold BF, et al. Effects of water quality, sanitation, handwashing, and nutritional interventions on diarrhoea and child growth in rural Bangladesh: a cluster randomised controlled trial. Lancet Glob Health 2018; 6: e302–15.

2. Parvez SM, Azad R, Rahman M, et al. Achieving optimal technology and behavioral uptake of single and combined interventions of water, sanitation hygiene and nutrition, in an efficacy trial (WASH benefits) in rural Bangladesh. Trials 2018; 19: 358.

# Supplement 3. Additional details about mediation analysis

To identify potential pathways through which WASH and nutrition interventions affect antibiotic use, we estimated the natural indirect effect (NIE) for mediators with significant mediator-outcome relationships (p-value < 0.05). The NIE is the difference in potential outcomes under the predicted values of the mediator if all children had been in intervention arm versus if all children had been in the control arm. This estimates how WASH interventions modify the prevalence of antibiotic use by shifting the distribution of a given mediator. The NIE can be estimated under potential outcome models that hold intervention status constant to treated (the “total” NIE) or control (the “pure” NIE).^1^ In the absence of intervention -mediator interaction, the total and pure NIEs are equivalent. In the presence of intervention -mediator interaction, the total NIE captures the influence of the interaction, while the pure NIE represents the mediated effect in the absence of interaction. Here, we reported the total NIE for all mediation analyses and additionally reported the pure NIE when we found evidence of intervention -mediator interaction (differences in NIE in the intervention vs control greater than 1% or t-test p-value < 0.2 when an intervention-mediator interaction term was included in the outcome model).

The NIE is estimated while holding either intervention status constant to treated (the “total” NIE) or control (the “pure” NIE). The total NIE captures the influence of the interaction, while the pure NIE represents the mediated effect in the absence of interaction. We considered potential intervention-mediator interaction (differences in NIE in the intervention vs control greater than 1% or t-test p-value < 0.2); in the absence of interaction, the total and pure natural indirect effects are approximately equal, and we reported the total NIE. When interaction was present, we reported the total and pure NIE separately.

1. VanderWeele TJ. A Three-way Decomposition of a Total Effect into Direct, Indirect, and Interactive Effects. Epidemiology. 2013 Mar;24(2):224–32.


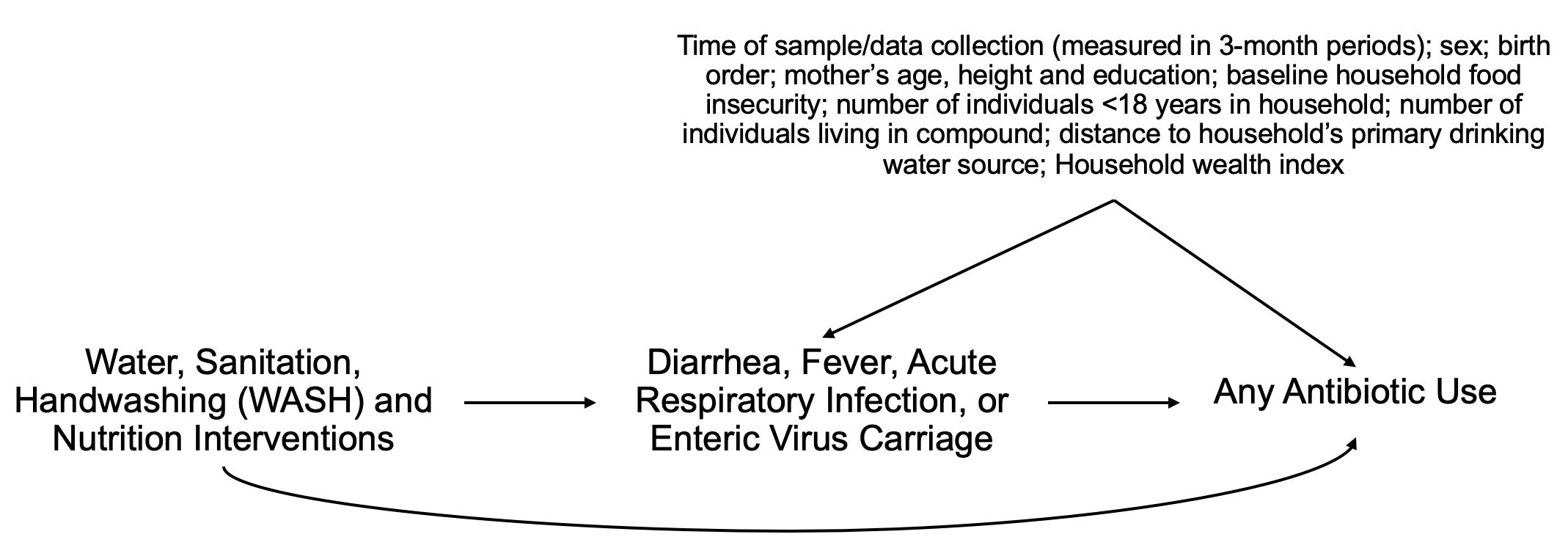


# Figure S1: Directed Acyclic Graph of Assumed Causal Model

Directed acyclic graph depicting the causal relationships that we assume in the mediation analysis. We assume no unmeasured confounding of the intervention-outcome relationship, since interventions were randomly assigned. We assume no mediator-outcome confounding after covariate adjustment. All covariates were measured at baseline except for month of measurement, child age, and child sex.


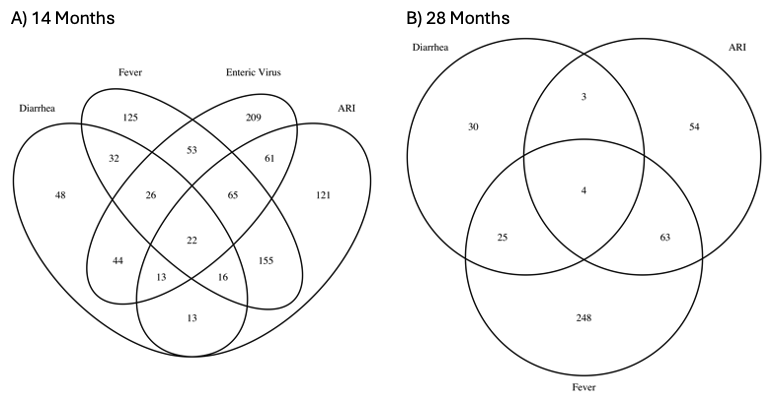


# Figure S2: Joint Prevalence of Mediators

Venn diagram of the number of children with various combinations of co-occurring mediators at measurements taken at approximately 14 and 28 months of age. Diarrhea, ARI, and Fever are all reported by a caregiver under a 7-day lookback period at 14 and 28 months. Enteric Virus is the presence of adenovirus 40/41, norovirus GI, norovirus GII, sapovirus, rotavirus, or astrovirus in stool collected at 14 months.


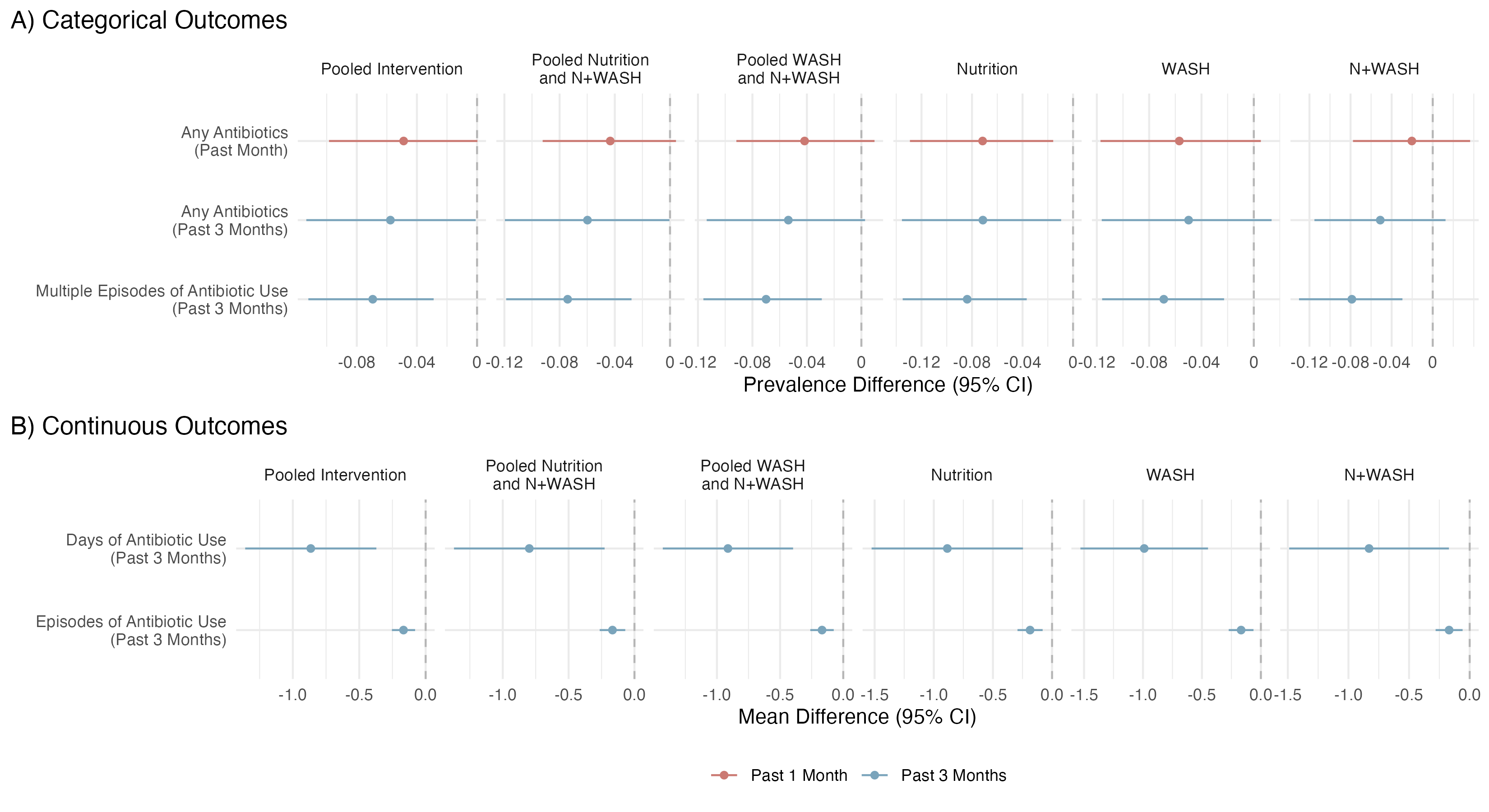


# Figure S3: Intervention-Outcome Effects, All Intervention Groups

(A) Prevalence differences and 95% confidence intervals for the effects of WASH and/or Nutrition interventions on categorical measures of antibiotic use and (B) mean differences and 95% confidence intervals for the effects of WASH and/or Nutrition interventions on continuous measures of antibiotic use. Antibiotic use is reported by a caregiver under either a 1- or 3-month look back period at 14 and 28 months.


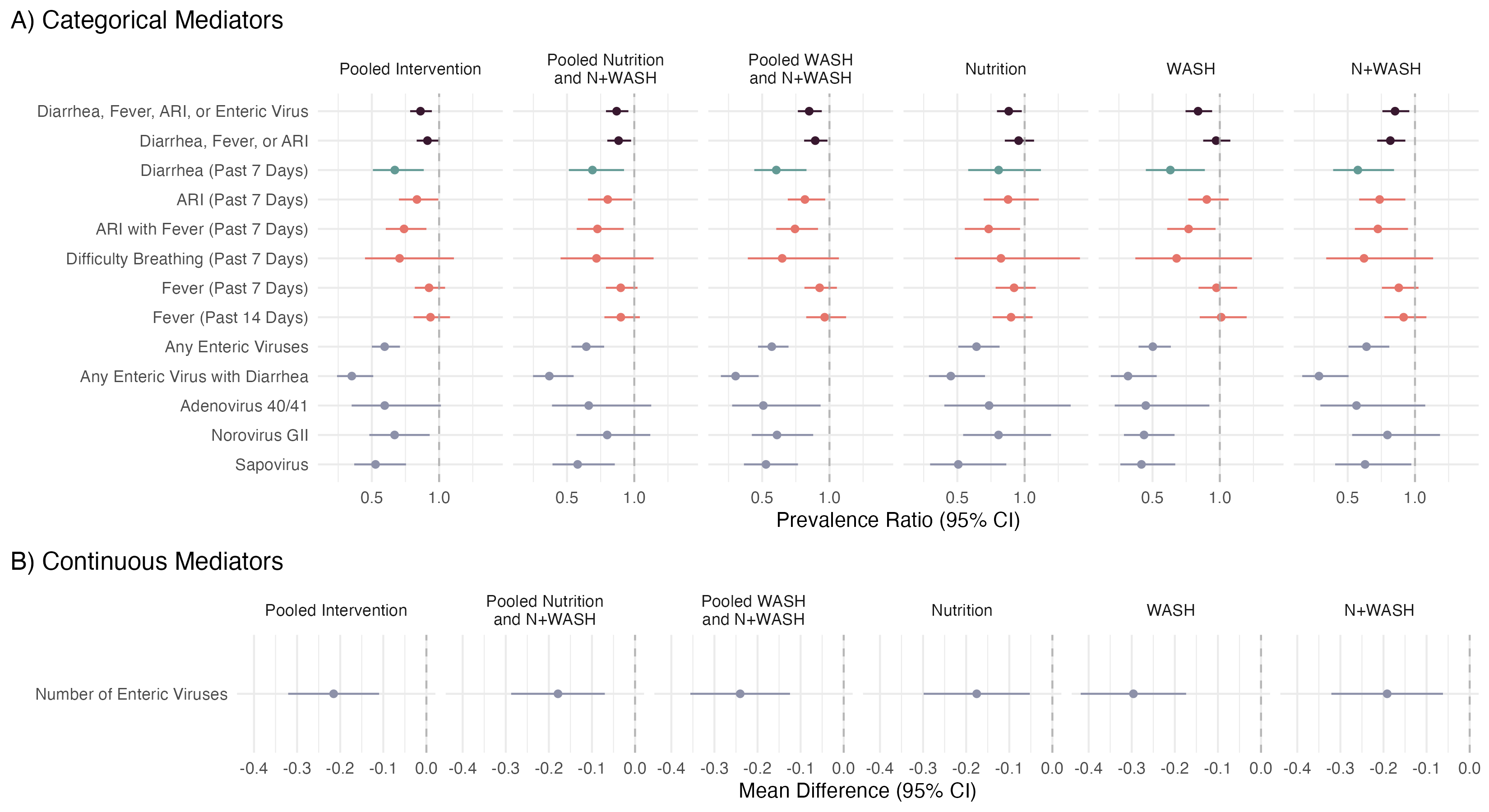


# Figure S4: Intervention-Mediator Effects, All Intervention Groups and Mediators

(A) Prevalence differences and 95% confidence intervals for the effects of WASH and/or Nutrition interventions on categorical mediators and (B) Prevalence differences and 95% confidence intervals for the effects of WASH and/or Nutrition interventions on continuous mediators. Diarrhea, ARI, ARI with Fever, and Fever are all reported by a caregiver under a 7-day lookback period at 14 and 28 months. Any Enteric Virus is the presence of adenovirus 40/41, norovirus GI, norovirus GII, sapovirus, rotavirus, or astrovirus in stool collected at 14 months, and Any Enteric Virus with Diarrhea is the presence of any enteric virus with caregiver reported diarrhea in the prior 7 days at 14 months.


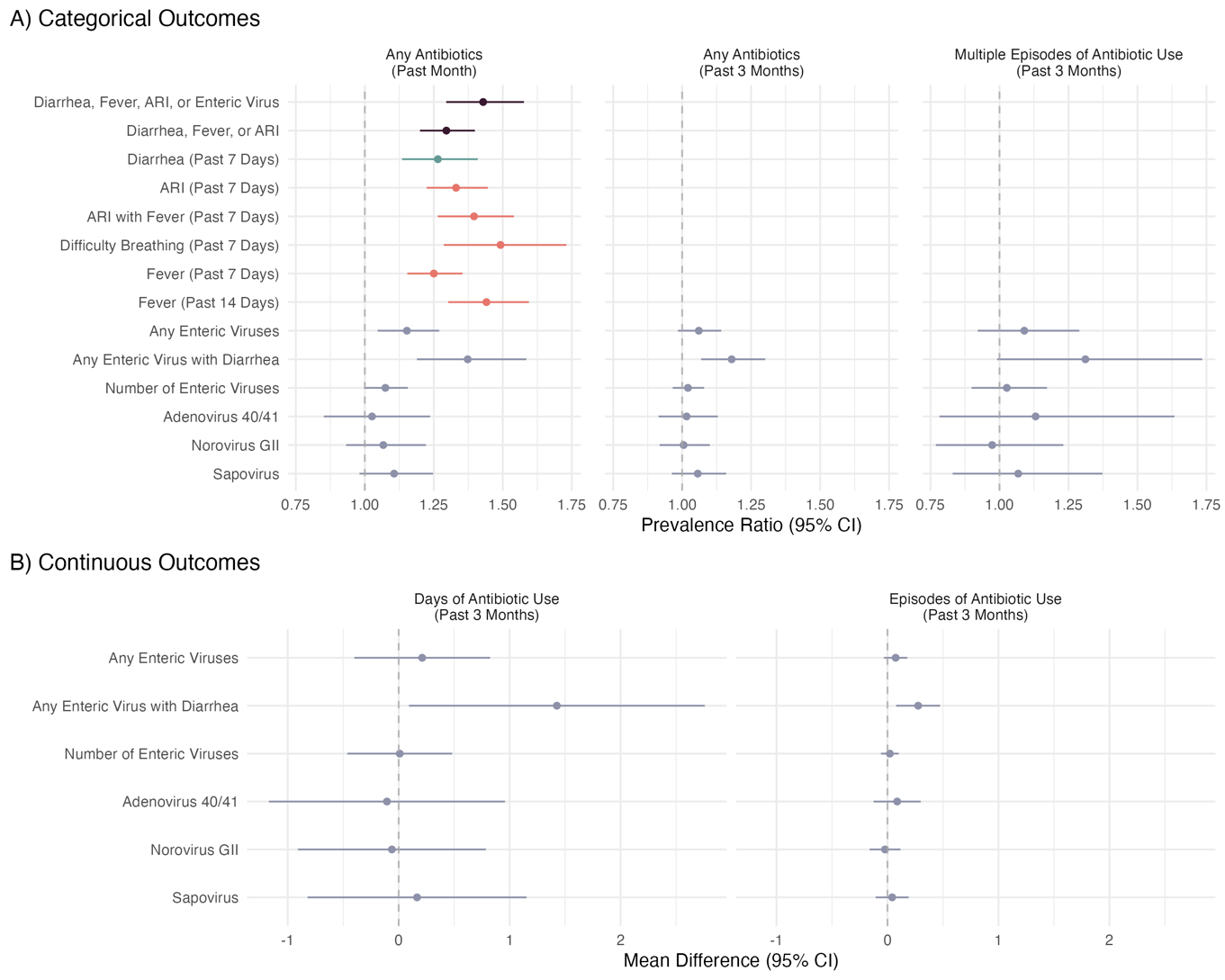


# Figure S5: Mediator-Outcome Effects, All Intervention Groups and Mediators

(A) Prevalence ratios and 95% confidence intervals for the effect of potential mediators on categorical measures of antibiotic use (B) Mean differences and 95% confidence intervals for the effect of potential mediators on continuous measures of antibiotic use. Diarrhea, ARI, ARI with Fever, and Fever are all reported by a caregiver under a 7-day lookback period at 14 and 28 months. Any Enteric Virus is the presence of adenovirus 40/41, norovirus GI, norovirus GII, sapovirus, rotavirus, or astrovirus in stool collected at 14 months, and Any Enteric Virus with Diarrhea is the presence of any enteric virus with caregiver reported diarrhea in the prior 7 days at 14 months. Antibiotic use is reported by a caregiver under either a 1- or 3-month lookback period at 14 and 28 months.


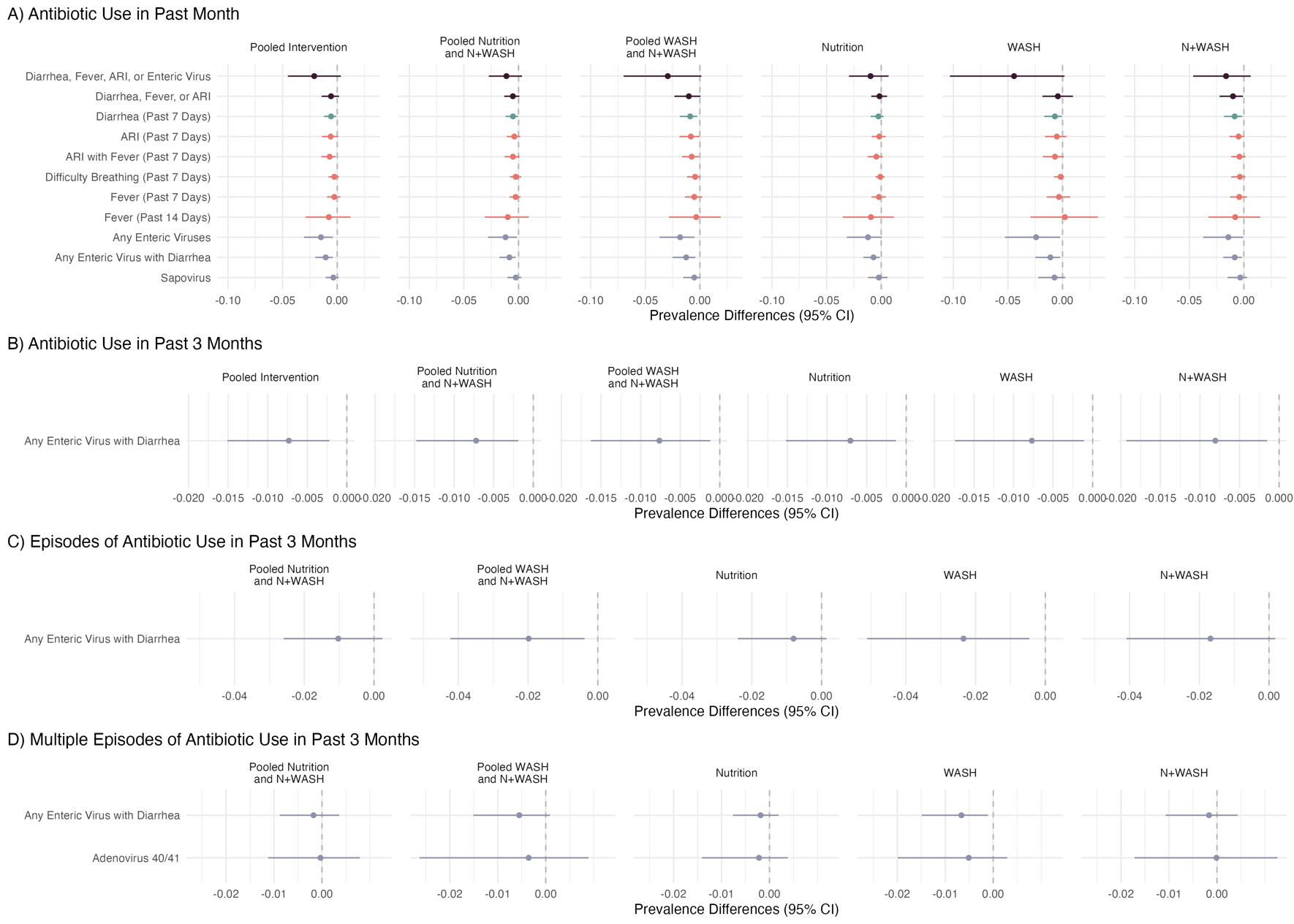


# Figure S6: Mediated Effects, All Intervention Groups and Mediators

Total natural indirect effect estimates and 95% confidence intervals of WASH and/or Nutrition interventions on antibiotic use, for significant mediator-outcome relationships (p-val < 0.05). Diarrhea, ARI, ARI with Fever, and Fever are all reported by a caregiver under a 7-day lookback period at 14 and 28 months. Any Enteric Virus is the presence of adenovirus 40/41, norovirus GI, norovirus GII, sapovirus, rotavirus, or astrovirus in stool collected at 14 months, and Any Enteric Virus with Diarrhea is the presence of any enteric virus with caregiver reported diarrhea in the prior 7 days at 14 months. Antibiotic use is reported by a caregiver under either a 1- or 3-month lookback period at 14 and 28 months.


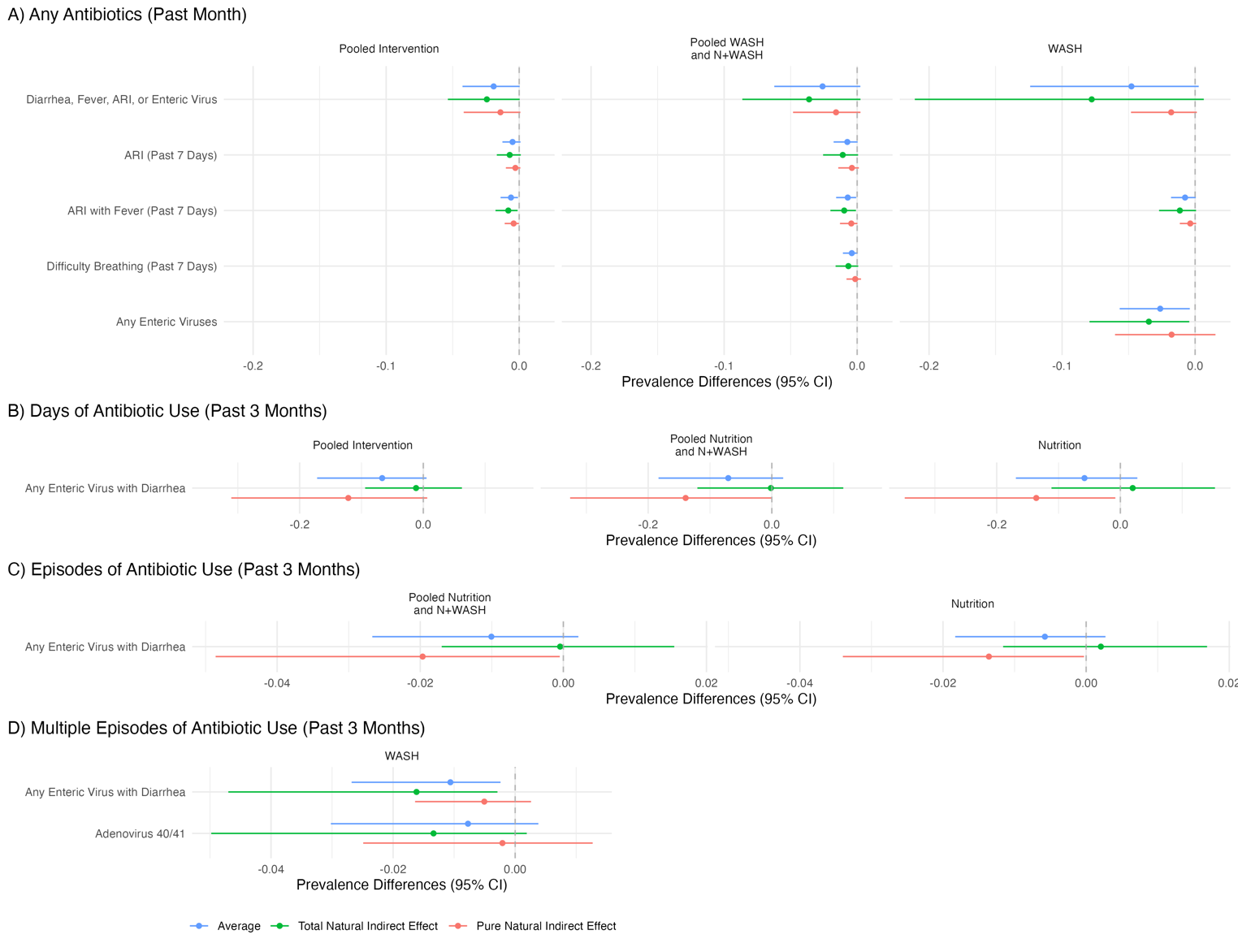


# Figure S7: Mediated Effects, with Significant Intervention-Mediator Interactions

Estimates and 95% CIs of total, pure, and averaged natural indirect effects, computed only when the difference in total and pure natural indirect effects was greater than 1% or had a t-test p-value < 0.2 when outcome models included an intervention-mediator interaction term.


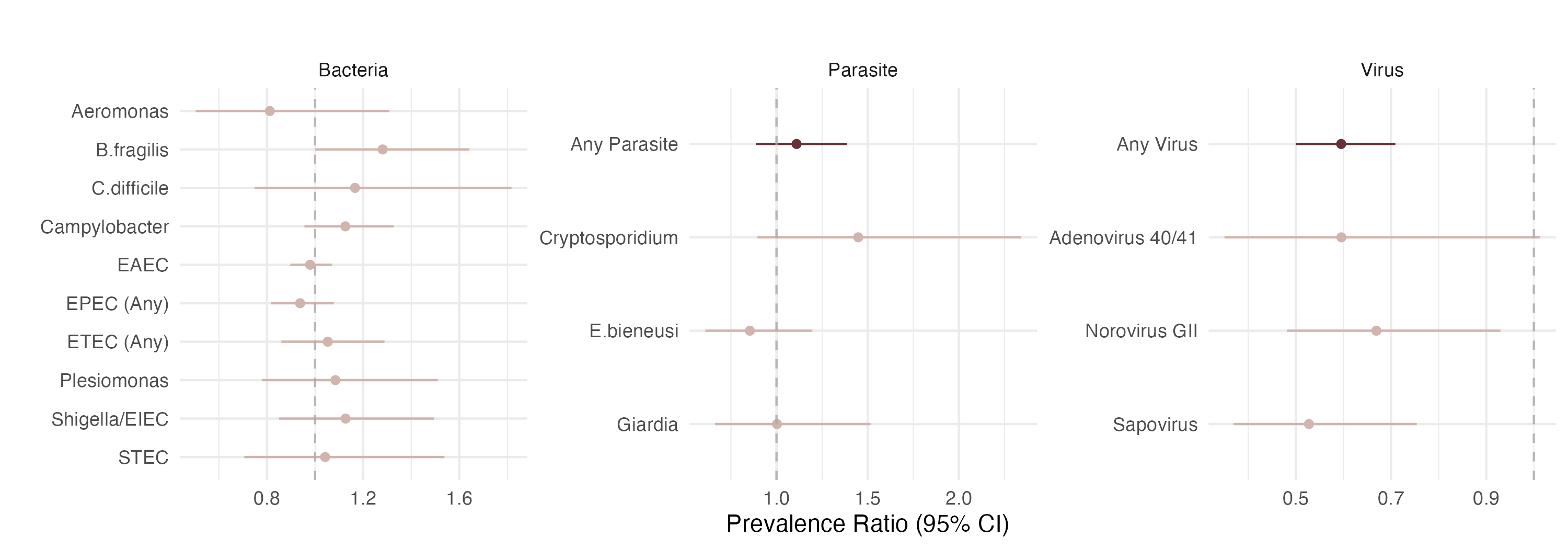


# Figure S8: Intervention-Mediator Effects, Pooled Intervention Group and Enteric Pathogen Carriage

Prevalence differences and 95% confidence intervals for the effects of any WASH or Nutrition intervention on enteric pathogen carriage assessed via qPCR. Pathogens with <5% prevalence in the study population are excluded


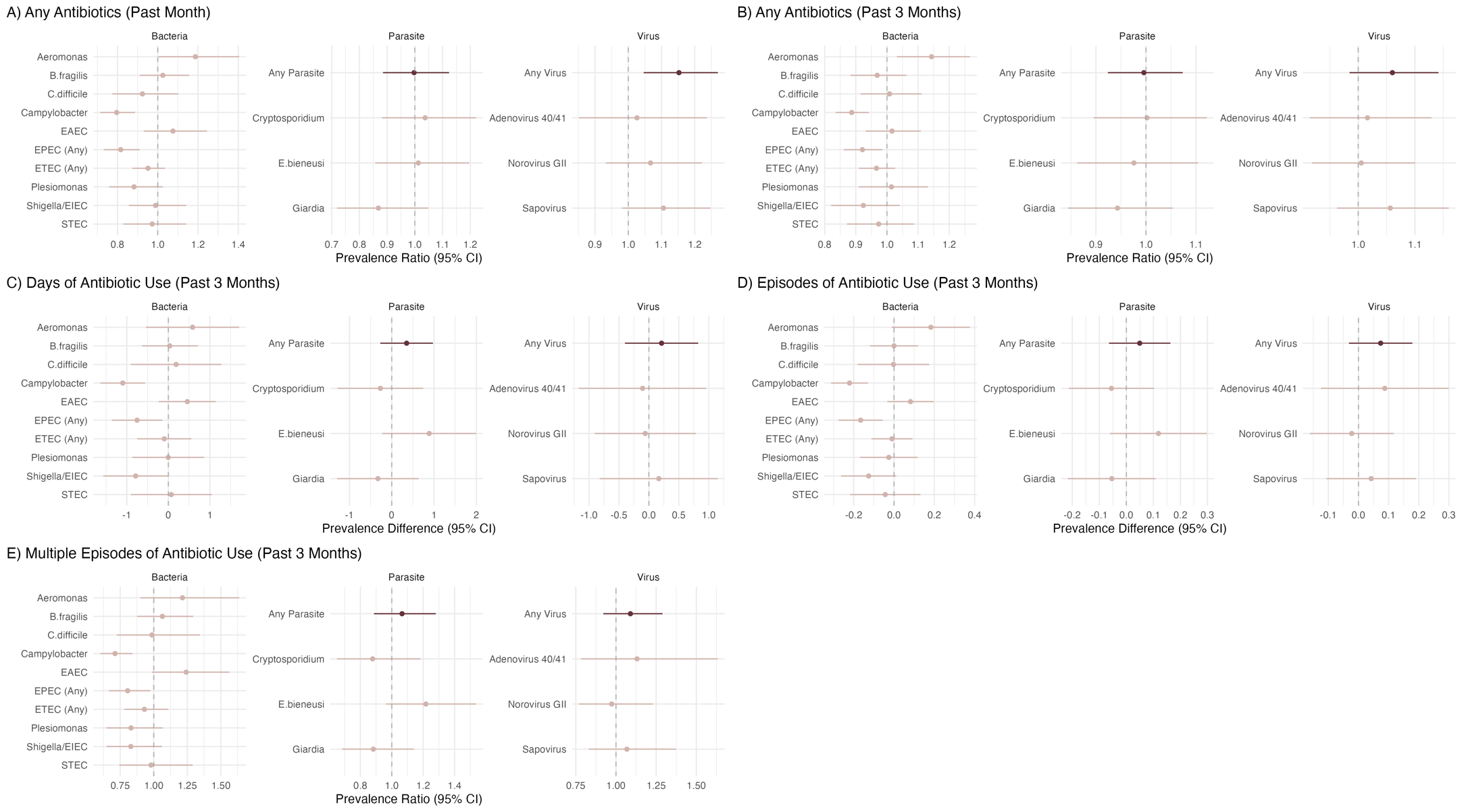


# Figure S9: Intervention-Mediator Effects, Pooled Intervention Group and Enteric Pathogen Carriage

Prevalence ratios and 95% confidence intervals for the effects of any WASH or Nutrition intervention on enteric pathogen carriage assessed via qPCR. Pathogens with <5% prevalence in the study population are excluded


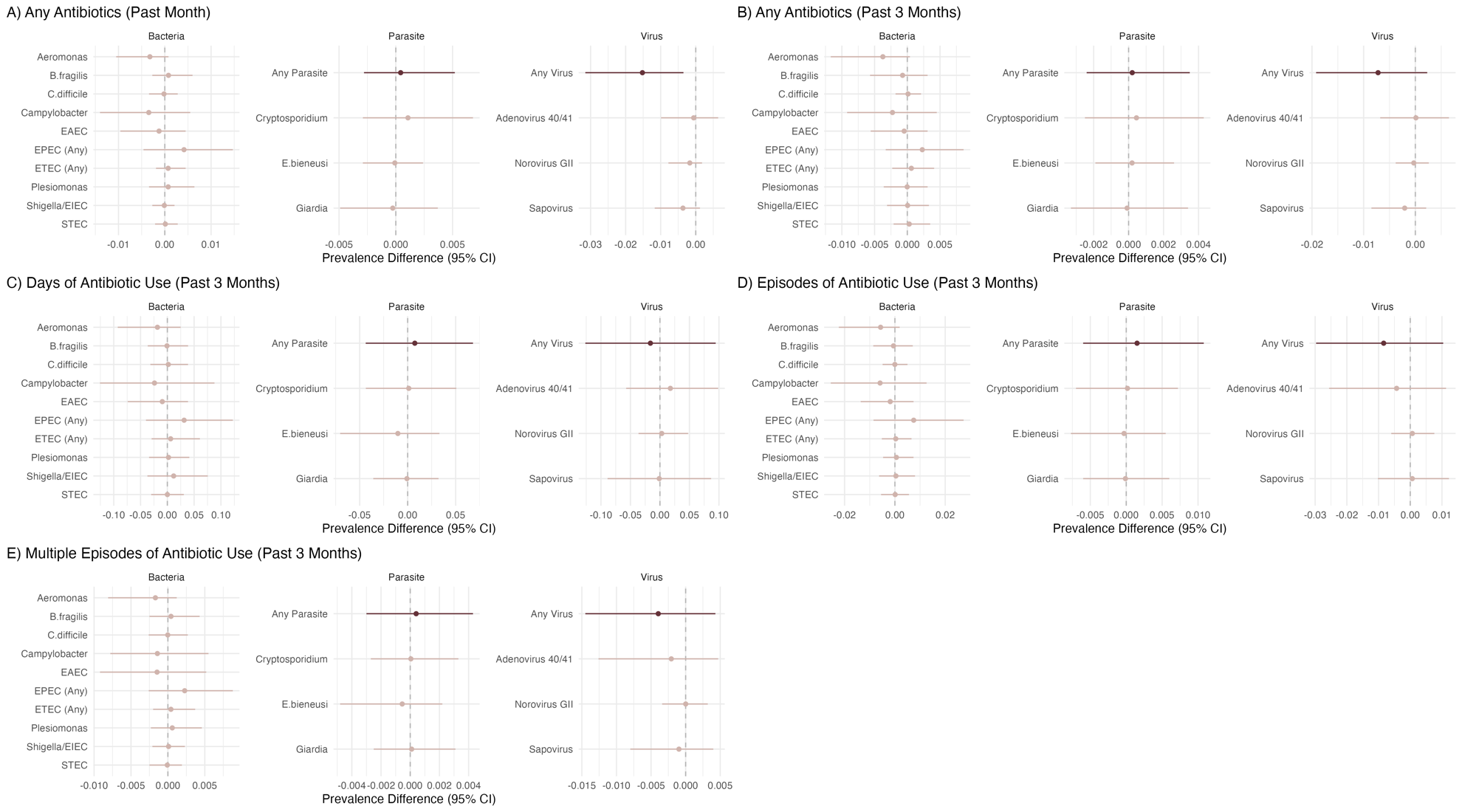


# Figure S10: Mediated Effects, Pooled Intervention Group and Enteric Pathogen Carriage

Total natural indirect effect estimates and 95% confidence intervals of any WASH or Nutrition intervention on antibiotic use, through enteric pathogen carriage assessed via qPCR

**
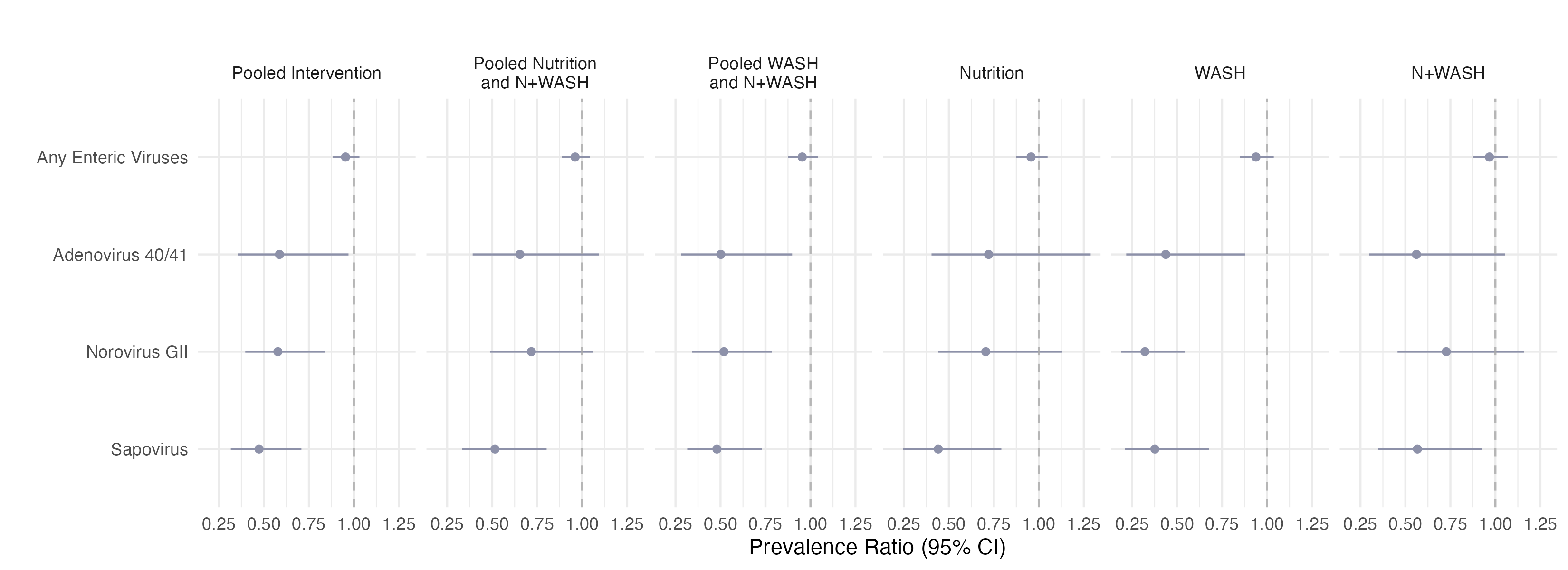
**

# Figure S11: Intervention-Mediator Effects, for Enteric Viruses with Diarrheal Etiology

Prevalence ratios and 95% confidence intervals for the effects of WASH and/or Nutrition interventions for enteric virus carriage at 14 months with pathogen loads that reflect diarrheal etiology based on published Ct cutoff values from the MAL-ED study. Any Enteric Virus is the presence of Adenovirus 40/41, Norovirus GII, or Sapovirus that exceeded the etiology cutoff.


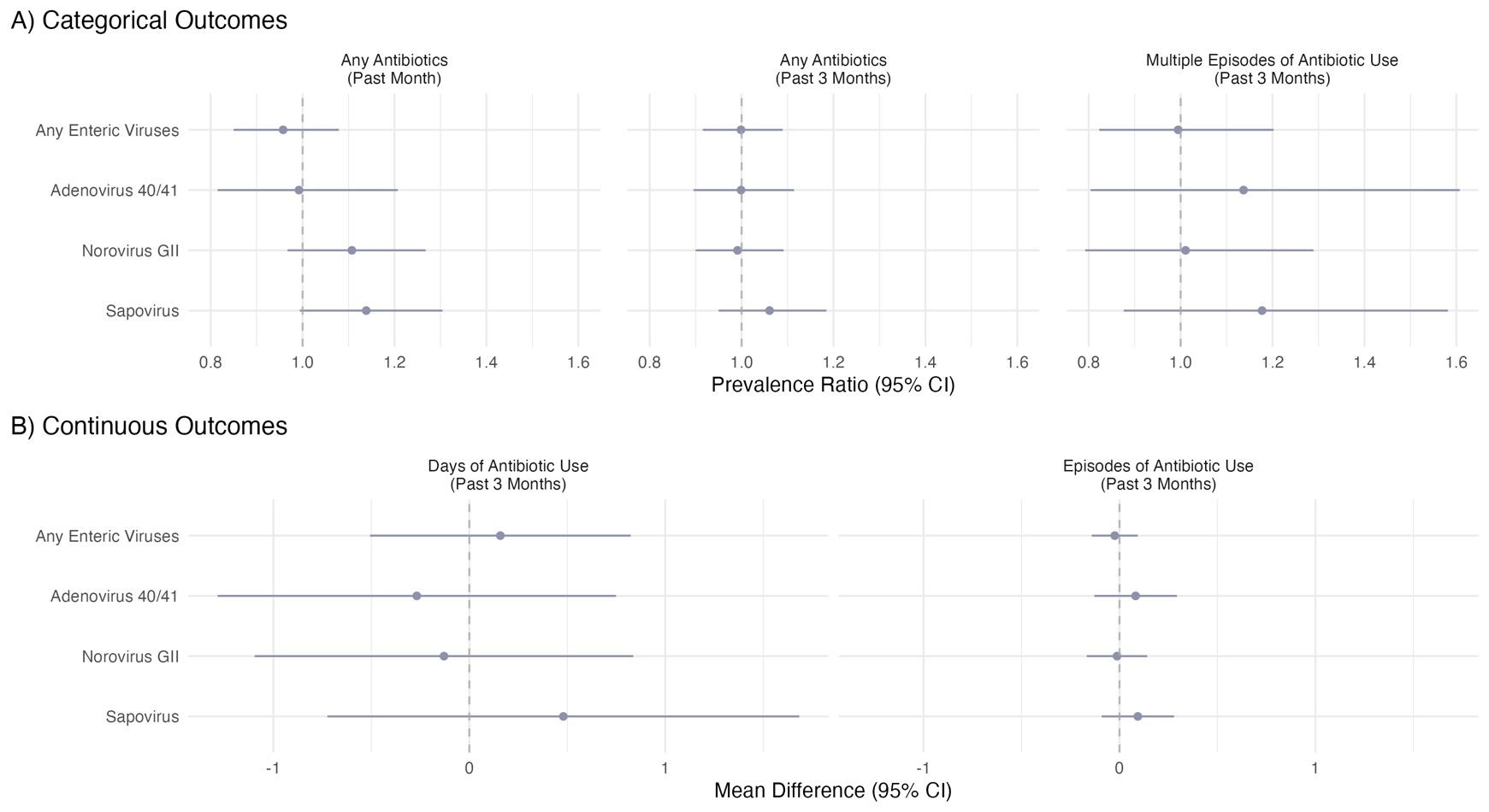


# Figure S12: Mediator-Outcome Effects, for Enteric Viruses with Diarrheal Etiology

(A) Prevalence ratios and 95% confidence intervals for the effect of enteric virus carriage at 14 months with pathogen loads that reflect diarrheal etiology on categorical measures of antibiotic use and (B) Mean differences and 95% confidence intervals for the effect of enteric virus carriage at 14 months with pathogen loads that reflect diarrheal etiology on continuous measures of antibiotic use. Diarrheal etiology is assessed using published Ct cutoff values from the MAL-ED study. Any Enteric Virus is the presence of Adenovirus 40/41, Norovirus GII, or Sapovirus that exceeded the etiology cutoff. Antibiotic use is reported by a caregiver under either a 1- or 3-month lookback period at 14 and 28 months.


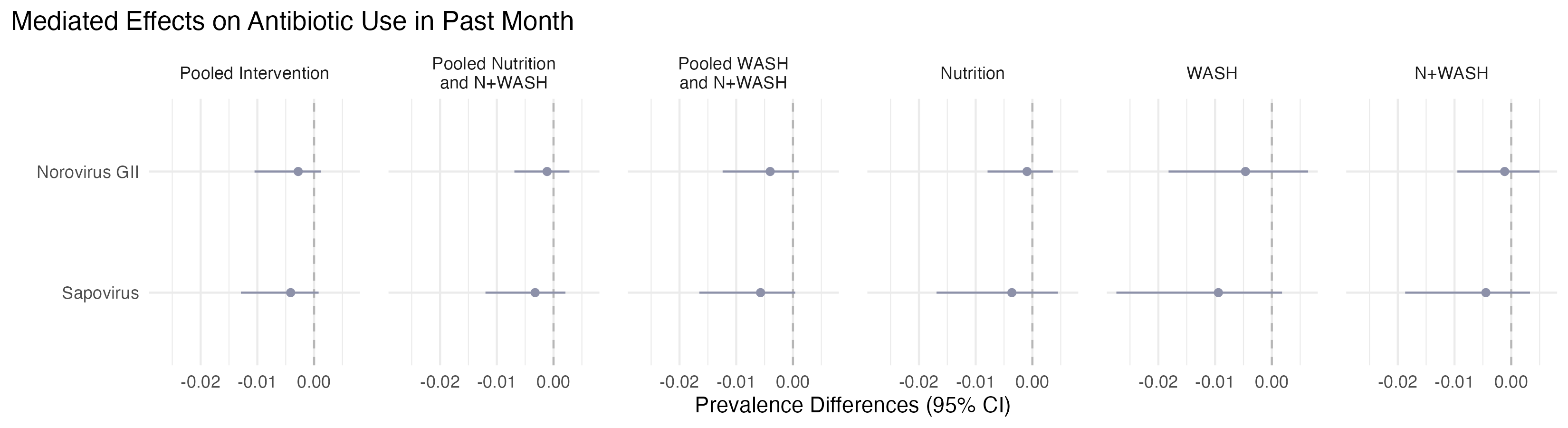


# Figure S13: Mediated Effects, for Enteric Viruses with Diarrheal Etiology

Total natural indirect effect estimates and 95% confidence intervals of WASH and/or Nutrition interventions on antibiotic use, for significant mediator-outcome relationships (p-val < 0.05) involving enteric virus carriage at 14 months with pathogen loads that reflect diarrheal etiology. Diarrheal etiology is assessed using published Ct cutoff values from the MAL-ED study. Any Enteric Virus is the presence of Adenovirus 40/41, Norovirus GII, or Sapovirus that exceeded the etiology cutoff. Antibiotic use is reported by a caregiver under either a 1- or 3-month lookback period at 14 and 28 months.


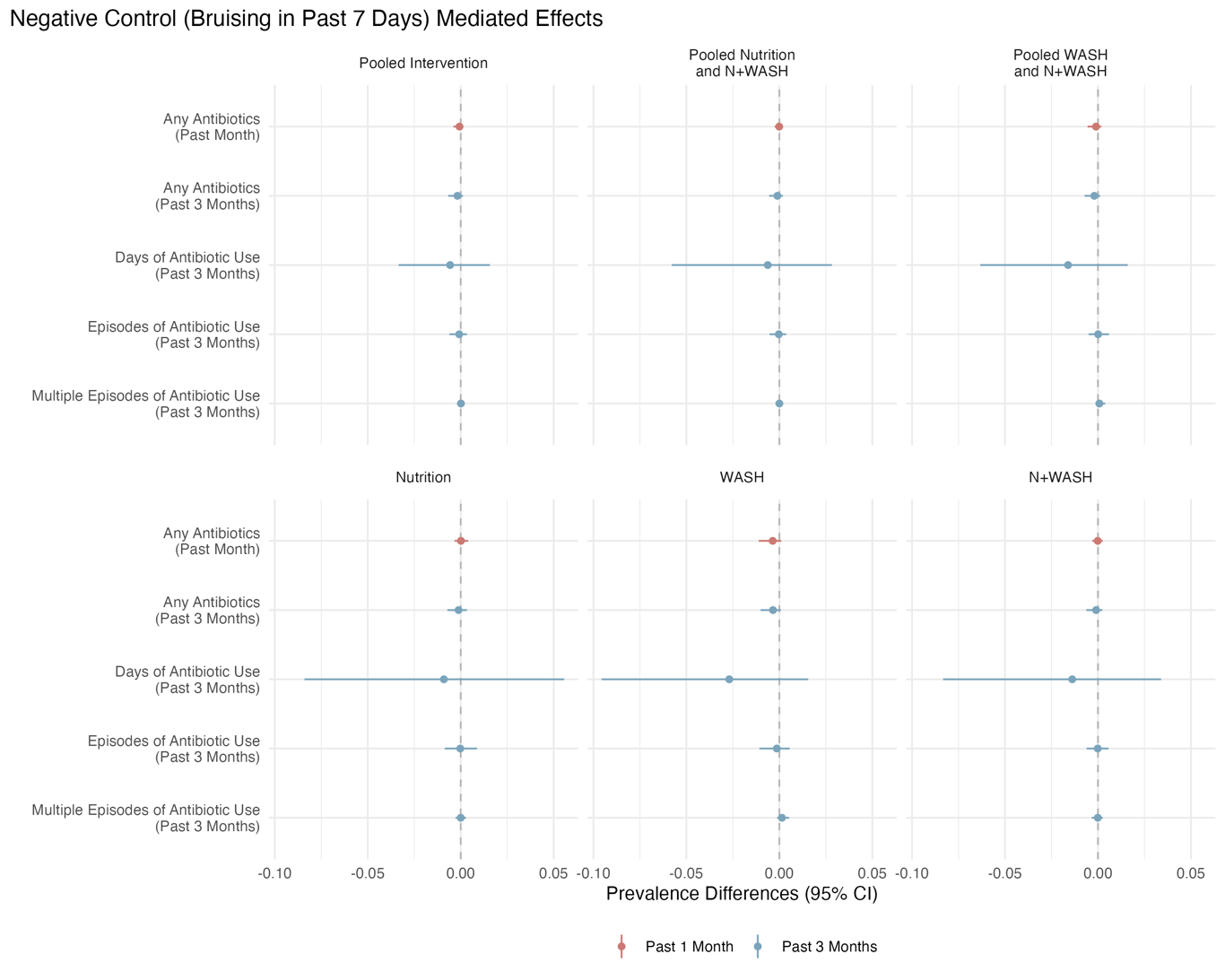


# Figure S14: Mediated Effects, Negative Control

Total natural indirect effect estimates and 95% confidence intervals of WASH and/or Nutrition interventions on antibiotic use through pathways involving bruising, a negative control. Bruising is reported by a caregiver under a 7-day lookback period at 14 and 28 months. Antibiotic use is reported by a caregiver under either a 1- or 3-month lookback period at 14 and 28 months.

# Table S1: Distribution of missing antibiotic use data across arms.

| **Intervention Group** | **Any Antibiotics (Past Month)** | **Any Antibiotics (Past 3 Months)** | **Days of Antibiotic Use (Past 3 Months)** | **Episodes of Antibiotic Use (Past 3 Months)** | **Multiple Episodes of Antibiotic Use (Past 3 Months)** |
| --- | --- | --- | --- | --- | --- |
| ***AGE: 14 MONTHS*** | | | | | |
| Control | 1 (0.27%) | 0 (0%) | 4 (1.06%) | 0 (0%) | 0 (0%) |
| Nutrition | 0 (0%) | 0 (0%) | 1 (0.27%) | 0 (0%) | 0 (0%) |
| Nutrition + WASH | 0 (0%) | 0 (0%) | 0 (0%) | 0 (0%) | 0 (0%) |
| WASH | 0 (0%) | 0 (0%) | 0 (0%) | 0 (0%) | 0 (0%) |
| ***AGE: 28 MONTHS*** | | | | | |
| Control | 0 (0%) | 0 (0%) | 1 (0.28%) | 0 (0%) | 0 (0%) |
| Nutrition | 3 (0.81%) | 0 (0%) | 0 (0%) | 0 (0%) | 0 (0%) |
| Nutrition + WASH | 4 (1%) | 0 (0%) | 1 (0.25%) | 0 (0%) | 0 (0%) |
| WASH | 1 (0.25%) | 0 (0%) | 0 (0%) | 0 (0%) | 0 (0%) |

# Table S2: Distribution of Baseline Characteristics, Mediators, and Antibiotic Use Outcomes, All Intervention Groups

|  | **Nutrition**  **(N = 419)** | **WASH**  **(N= 434)** | **NWASH**  **(N = 441)** | **Nutrition + NWASH**  **(N = 860)** | **WASH + NWASH**  **(N = 875)** | **Control**  **(N = 422)** |
| --- | --- | --- | --- | --- | --- | --- |
| **BASELINE COVARIATES** | | | | | | |
| Month of Measurement | | | | | | |
| *Feb-May* | 236 (31.51%) | 246 (31.06%) | 207 (26.54%) | 443 (28.97%) | 453 (28.82%) | 253 (34.42%) |
| *Jun-Sep* | 270 (36.05%) | 329 (41.54%) | 327 (41.92%) | 597 (39.05%) | 656 (41.73%) | 258 (35.1%) |
| *Oct-Jan* | 243 (32.44%) | 217 (27.4%) | 246 (31.54%) | 489 (31.98%) | 463 (29.45%) | 224 (30.48%) |
| Child's age | 15.71 (7.43) | 15.68 (7.44) | 15.95 (7.37) | 15.83 (7.39) | 15.81 (7.40) | 16.43 (7.40) |
| Child's sex | | | | | | |
| *Female* | 361 (48.20%) | 371 (46.84%) | 410 (52.56%) | 771 (50.43%) | 781 (49.68%) | 372 (50.61%) |
| *Male* | 388 (51.80%) | 421 (53.16%) | 370 (47.44%) | 758 (49.57%) | 791 (50.32%) | 363 (49.39%) |
| Birth Order | | | | | | |
| *1* | 269 (35.91%) | 248 (31.31%) | 269 (34.49%) | 538 (35.19%) | 517 (32.89%) | 272 (37.01%) |
| *2+* | 463 (61.82%) | 529 (66.79%) | 494 (63.33%) | 957 (62.59%) | 1023 (65.08%) | 416 (56.6%) |
| *Missing* | 17 (2.27%) | 15 (1.89%) | 17 (2.18%) | 34 (2.22%) | 32 (2.04%) | 47 (6.39%) |
| Mother's age | 23.75 (5.00) | 24.46 (5.38) | 24.24 (5.48) | 24.00 (5.26) | 24.35 (5.43) | 23.45 (4.85) |
| Mother's height | 150.41 (5.55) | 150.44 (5.46) | 150.05 (5.22) | 150.22 (5.38) | 150.25 (5.34) | 150.84 (5.16) |
| Mother's education | | | | | | |
| *No education* | 112 (14.95%) | 96 (12.12%) | 126 (16.15%) | 238 (15.57%) | 222 (14.12%) | 78 (10.61%) |
| *Primary (1-5y)* | 220 (29.37%) | 222 (28.03%) | 250 (32.05%) | 470 (30.74%) | 472 (30.03%) | 179 (24.35%) |
| *Secondary (>5y)* | 417 (55.67%) | 474 (59.85%) | 404 (51.79%) | 821 (53.7%) | 878 (55.85%) | 478 (65.03%) |
| Household food insecurity | | | | | | |
| *Food Secure* | 538 (71.83%) | 534 (67.42%) | 554 (71.03%) | 1092 (71.42%) | 1088 (69.21%) | 543 (73.88%) |
| *Mildly Food Insecure* | 57 (7.61%) | 77 (9.72%) | 67 (8.59%) | 124 (8.11%) | 144 (9.16%) | 62 (8.44%) |
| *Moderately/Severely*  *Food Insecure* | 154 (20.56%) | 181 (22.85%) | 159 (20.38%) | 313 (20.47%) | 340 (21.63%) | 130 (17.69%) |
| Number of individuals in  household under age 18 | 1.52 (1.30) | 1.65 (1.21) | 1.72 (1.29) | 1.62 (1.30) | 1.68 (1.25) | 1.56 (1.25) |
| Number of individuals in  compound | 11.33 (6.46) | 11.43 (6.38) | 11.46 (6.82) | 11.40 (6.65) | 11.44 (6.60) | 10.10 (6.07) |
| Distance to water source | 0.76 (1.55) | 1.14 (6.19) | 0.76 (2.23) | 0.76 (1.93) | 0.95 (4.67) | 0.75 (1.53) |
| Improved Roof Material  (tin, cement) | 738 (98.53%) | 781 (98.61%) | 770 (98.72%) | 1508 (98.63%) | 1551 (98.66%) | 729 (99.18%) |
| Improved Wall Material  (wood, brick, tin) | 507 (67.69%) | 498 (62.88%) | 525 (67.31%) | 1032 (67.5%) | 1023 (65.08%) | 449 (61.09%) |
| Improved Floor Material  (wood, concrete) | 89 (11.88%) | 91 (11.49%) | 94 (12.05%) | 183 (11.97%) | 185 (11.77%) | 121 (16.46%) |
| Household Wealth Index | | | | | | |
| *Wealth Q1* | 176 (23.5%) | 180 (22.73%) | 160 (20.51%) | 336 (21.98%) | 340 (21.63%) | 100 (13.61%) |
| *Wealth Q2* | 178 (23.77%) | 167 (21.09%) | 195 (25%) | 373 (24.4%) | 362 (23.03%) | 138 (18.78%) |
| *Wealth Q3* | 130 (17.36%) | 186 (23.48%) | 156 (20%) | 286 (18.71%) | 342 (21.76%) | 185 (25.17%) |
| *Wealth Q4* | 178 (23.77%) | 173 (21.84%) | 172 (22.05%) | 350 (22.89%) | 345 (21.95%) | 193 (26.26%) |
| *Missing* | 87 (11.62%) | 86 (10.86%) | 97 (12.44%) | 184 (12.03%) | 183 (11.64%) | 119 (16.19%) |
| **MEDIATORS** | | | | | | |
| Diarrhea, Fever, ARI, or  Enteric Virus | 232 (66.67%) | 225 (63.03%) | 226 (64.94%) | 458 (65.8%) | 451 (63.97%) | 238 (76.28%) |
| Diarrhea Fever, or ARI | 308 (41.45%) | 332 (42.29%) | 275 (35.48%) | 583 (38.41%) | 607 (38.91%) | 306 (42.21%) |
| Diarrhea | 75 (10.09%) | 62 (7.9%) | 55 (7.1%) | 130 (8.56%) | 117 (7.5%) | 84 (11.59%) |
| ARI | 145 (19.52%) | 160 (20.38%) | 128 (16.52%) | 273 (17.98%) | 288 (18.46%) | 157 (21.66%) |
| ARI with Fever  (Past 7 Days) | 73 (9.83%) | 82 (10.45%) | 75 (9.68%) | 148 (9.75%) | 157 (10.06%) | 95 (13.1%) |
| Fever (Past 7 Days) | 201 (27.05%) | 225 (28.66%) | 199 (25.68%) | 400 (26.35%) | 424 (27.18%) | 209 (28.83%) |
| Number of Enteric Viruses | 0.41 (0.64) | 0.32 (0.56) | 0.41 (0.63) | 0.41 (0.63) | 0.36 (0.59) | 0.60 (0.63) |
| Any Enteric Viruses | 116 (33.33%) | 98 (27.45%) | 116 (33.33%) | 232 (33.33%) | 214 (30.35%) | 163 (52.24%) |
| **ANTIBIOTIC OUTCOMES** | | | | | | |
| Any Antibiotics  (Past Month) | 324 (43.43%) | 350 (44.25%) | 366 (47.16%) | 690 (45.34%) | 716 (45.69%) | 363 (49.46%) |
| Any Antibiotics  (Past 3 Months) | 440 (58.74%) | 484 (61.11%) | 473 (60.64%) | 913 (59.71%) | 957 (60.88%) | 489 (66.53%) |
| Days of Antibiotic Use  (Past 3 Months) | 4.38 (5.41) | 4.32 (4.99) | 4.33 (5.12) | 4.36 (5.26) | 4.33 (5.05) | 5.17 (5.59) |
| Episodes of Antibiotic Use  (Past 3 Months) | 0.84 (0.92) | 0.86 (0.88) | 0.84 (0.87) | 0.84 (0.89) | 0.85 (0.87) | 1.00 (0.95) |
| Multiple Episodes of  Antibiotic Use  (Past 3 Months) | 133 (17.76%) | 156 (19.7%) | 138 (17.69%) | 271 (17.72%) | 294 (18.7%) | 181 (24.63%) |

Sample sizes, child demographics, household characteristics, mediator prevalence, and antibiotic use prevalence in each intervention group. For categorical variables, the number of occurrences and percentages are reported. For continuous variables, the mean and standard deviation (SD) are reported.

# Table S3: Distribution of Baseline Characteristics, Mediators, and Antibiotic Use Outcomes, By Intervention Arm and Follow-Up Period

|  | **Any WASH Intervention**  **(N = 1,080)** | | **Control**  **(N = 329)** | |
| --- | --- | --- | --- | --- |
|  | **14 Mos.**  **N=1,151** | **28 Mos.**  **N=1,170** | **14 Mos.**  **N=377** | **28 Mos**  **N=358** |
| **BASELINE COVARIATES** | | | | |
| Month of Measurement | | | | |
| *Feb-May* | 265 (23.02%) | 424 (36.24%) | 139 (36.87%) | 114 (31.84%) |
| *Jun-Sep* | 633 (55%) | 293 (25.04%) | 89 (23.61%) | 169 (47.21%) |
| *Oct-Jan* | 253 (21.98%) | 453 (38.72%) | 149 (39.52%) | 75 (20.95%) |
| Child's age | 8.56 (1.74) | 22.84 (2.15) | 9.42 (1.72) | 23.74 (1.98) |
| Child's sex | | | | |
| *Female* | 574 (49.87%) | 568 (48.55%) | 189 (50.13%) | 183 (51.12%) |
| *Male* | 577 (50.13%) | 602 (51.45%) | 188 (49.87%) | 175 (48.88%) |
| Birth Order | | | | |
| *1* | 383 (33.28%) | 403 (34.44%) | 139 (36.87%) | 133 (37.15%) |
| *2+* | 730 (63.42%) | 756 (64.62%) | 206 (54.64%) | 210 (58.66%) |
| *Missing* | 38 (3.3%) | 11 (0.94%) | 32 (8.49%) | 15 (4.19%) |
| Mother's age | 24.18 (5.33) | 24.14 (5.27) | 23.54 (4.83) | 23.37 (4.87) |
| Mother's height | 150.31 (5.44) | 150.28 (5.38) | 150.84 (5.12) | 150.83 (5.21) |
| Mother's education | | | | |
| *No education* | 168 (14.6%) | 166 (14.19%) | 43 (11.41%) | 35 (9.78%) |
| *Primary (1-5y)* | 340 (29.54%) | 352 (30.09%) | 91 (24.14%) | 88 (24.58%) |
| *Secondary (>5y)* | 643 (55.86%) | 652 (55.73%) | 243 (64.46%) | 235 (65.64%) |
| Household food insecurity | | | | |
| *Food Secure* | 807 (70.11%) | 819 (70%) | 280 (74.27%) | 263 (73.46%) |
| *Mildly Food Insecure* | 98 (8.51%) | 103 (8.8%) | 29 (7.69%) | 33 (9.22%) |
| *Moderately/Severely Food Insecure* | 246 (21.37%) | 248 (21.2%) | 68 (18.04%) | 62 (17.32%) |
| Number of individuals in household under age 18 | 1.62 (1.27) | 1.64 (1.28) | 1.55 (1.24) | 1.56 (1.27) |
| Number of individuals in compound | 11.43 (6.59) | 11.39 (6.51) | 10.05 (6.13) | 10.16 (6.01) |
| Distance to water source | 0.88 (3.94) | 0.89 (3.94) | 0.76 (1.53) | 0.75 (1.54) |
| Improved Roof Material  (tin, cement) | 1136 (98.7%) | 1153 (98.55%) | 374 (99.2%) | 355 (99.16%) |
| Improved Wall Material  (wood, brick, tin) | 754 (65.51%) | 776 (66.32%) | 227 (60.21%) | 222 (62.01%) |
| Improved Floor Material  (wood, concrete) | 136 (11.82%) | 138 (11.79%) | 63 (16.71%) | 58 (16.2%) |
| Household Wealth Index | | | | |
| *Wealth Q1* | 273 (23.72%) | 243 (20.77%) | 53 (14.06%) | 47 (13.13%) |
| *Wealth Q2* | 285 (24.76%) | 255 (21.79%) | 72 (19.1%) | 66 (18.44%) |
| *Wealth Q3* | 247 (21.46%) | 225 (19.23%) | 99 (26.26%) | 86 (24.02%) |
| *Wealth Q4* | 274 (23.81%) | 249 (21.28%) | 105 (27.85%) | 88 (24.58%) |
| *Missing* | 72 (6.26%) | 198 (16.92%) | 48 (12.73%) | 71 (19.83%) |
| **MEDIATORS** | | | | |
| Diarrhea, Fever, ARI, or Enteric Virus | 683 (64.86%) | NA | 238 (76.28%) | NA |
| Diarrhea Fever, or ARI | 596 (51.78%) | 319 (27.69%) | 198 (52.52%) | 108 (31.03%) |
| Diarrhea | 147 (12.77%) | 45 (3.91%) | 67 (17.77%) | 17 (4.89%) |
| ARI | 347 (30.15%) | 86 (7.47%) | 119 (31.56%) | 38 (10.92%) |
| ARI with Fever | 183 (15.9%) | 47 (4.08%) | 75 (19.89%) | 20 (5.75%) |
| Fever (Past 7 Days) | 368 (31.97%) | 257 (22.31%) | 126 (33.42%) | 83 (23.85%) |
| Number of Enteric Viruses | 0.38 (0.61) | NA | 0.60 (0.63) | NA |
| Any Enteric Viruses | 330 (31.34%) | NA | 163 (52.24%) | NA |
| **ANTIBIOTIC OUTCOMES** | | | | |
| Any Antibiotics  (Past Month) | 621 (53.95%) | 419 (36.06%) | 221 (58.78%) | 142 (39.66%) |
| Any Antibiotics  (Past 3 Months) | 807 (70.11%) | 590 (50.43%) | 283 (75.07%) | 206 (57.54%) |
| Days of Antibiotic Use (Past 3 Months) | 5.39 (5.61) | 3.32 (4.47) | 6.27 (5.91) | 4.01 (4.99) |
| Episodes of Antibiotic Use  (Past 3 Months) | 1.06 (0.96) | 0.65 (0.76) | 1.23 (0.99) | 0.77 (0.84) |
| Multiple Episodes of Antibiotic Use  (Past 3 Months) | 295 (25.63%) | 132 (11.28%) | 131 (34.75%) | 50 (13.97%) |

Sample sizes, child characteristics, mediator prevalence, and antibiotic use prevalence in each intervention group, by follow-up period. For categorical variables, the number of occurrences and percentages are reported. For continuous variables, the mean and standard deviation (SD) are reported.

# Table S4: Joint Prevalence of Mediators

| **Age** | **Mediator** | **Pooled Intervention** | **Control** |
| --- | --- | --- | --- |
| Pooled Ages | Diarrhea only | 34 (1.5%) | 8 (1.1%) |
|  | ARI only | 87 (3.7%) | 13 (1.8%) |
|  | Fever only | 86 (3.7%) | 16 (2.2%) |
|  | Enteric Virus only | 138 (5.9%) | 71 (9.7%) |
|  | Diarrhea + ARI | 8 (0.3%) | 1 (0.1%) |
|  | Diarrhea + Fever | 26 (1.1%) | 4 (0.5%) |
|  | Diarrhea + ARI + Fever | 10 (0.4%) | 4 (0.5%) |
|  | ARI + Enteric Virus | 43 (1.9%) | 18 (2.4%) |
|  | Diarrhea + ARI + Fever + Enteric Virus | 14 (0.6%) | 8 (1.1%) |
| 14 months | Diarrhea only | 34 (3.0%) | 8 (2.1%) |
|  | ARI only | 87 (7.6%) | 13 (3.4%) |
|  | Fever only | 86 (7.5%) | 16 (4.2%) |
|  | Enteric Virus only | 138 (12.0%) | 71 (18.8%) |
|  | Diarrhea + ARI | 8 (0.7%) | 1 (0.3%) |
|  | Diarrhea + Fever | 26 (2.3%) | 4 (1.1%) |
|  | Diarrhea + ARI + Fever | 10 (0.9%) | 4 (1.1%) |
|  | ARI + Enteric Virus | 43 (3.7%) | 18 (4.8%) |
|  | Diarrhea + ARI + Fever + Enteric Virus | 14 (1.2%) | 8 (2.1%) |
| 28 months | Diarrhea only | 23 (2.0%) | 7 (2.0%) |
|  | ARI only | 38 (3.2%) | 16 (4.5%) |
|  | Fever only | 191 (16.3%) | 57 (15.9%) |
|  | Diarrhea + ARI | 1 (0.1%) | 2 (0.6%) |
|  | Diarrhea + Fever | 19 (1.6%) | 6 (1.7%) |
|  | Diarrhea + ARI + Fever | 2 (0.2%) | 2 (0.6%) |

Number and percentage of children with various combinations of co-occurring mediators in measurements taken at 14 and/or 28 months of age

# Table S5: Intervention-Outcome Effects

| **Intervention Group** | **Outcome** | **Prevalence/Mean in Control Arm** | **Prevalence/Mean in Intervention Arm** | **Prevalence/Mean Difference (95% CI)** | **Prevalence Ratio (95% CI)** |
| --- | --- | --- | --- | --- | --- |
| Pooled Intervention | Any antibiotic use in past month | 0.495 (0.459, 0.530) | 0.450 (0.427, 0.472) | -0.055 (-0.099, -0.012) | 0.890 (0.813, 0.974) |
|  | Any antibiotic use in past 3 months | 0.665 (0.618, 0.713) | 0.602 (0.581, 0.623) | -0.072 (-0.124, -0.017) | 0.892 (0.822, 0.967) |
|  | Days of antibiotic use in past 3 months | 5.167 (4.724, 5.610) | 4.345 (4.120, 4.571) | -0.929 (-1.405, -0.453) | ---- |
|  | Episodes of antibiotic use in past 3 months | 1.004 (0.929, 1.079) | 0.850 (0.812, 0.889) | -0.175 (-0.257, -0.092) | ---- |
|  | Multiple episodes of antibiotic use | 0.246 (0.214, 0.279) | 0.184 (0.167, 0.201) | -0.071 (-0.105, -0.038) | 0.719 (0.623, 0.829) |
| Pooled Nutrition and Nutrition + WASH | Any antibiotic use in past month | 0.495 (0.459, 0.530) | 0.453 (0.425, 0.481) | -0.050 (-0.097, -0.002) | 0.901 (0.815, 0.997) |
|  | Any antibiotic use in past 3 months | 0.665 (0.618, 0.713) | 0.597 (0.570, 0.625) | -0.074 (-0.124, -0.019) | 0.887 (0.815, 0.965) |
|  | Days of antibiotic use in past 3 months | 5.167 (4.724, 5.610) | 4.357 (4.029, 4.685) | -0.917 (-1.443, -0.390) | ---- |
|  | Episodes of antibiotic use in past 3 months | 1.004 (0.929, 1.079) | 0.843 (0.791, 0.895) | -0.181 (-0.269, -0.092) | ---- |
|  | Multiple episodes of antibiotic use | 0.246 (0.214, 0.279) | 0.177 (0.157, 0.198) | -0.077 (-0.117, -0.041) | 0.692 (0.587, 0.815) |
| Pooled WASH and Nutrition + WASH | Any antibiotic use in past month | 0.495 (0.459, 0.530) | 0.457 (0.431, 0.483) | -0.049 (-0.094, -0.005) | 0.903 (0.823, 0.991) |
|  | Any antibiotic use in past 3 months | 0.665 (0.618, 0.713) | 0.609 (0.582, 0.636) | -0.065 (-0.122, -0.009) | 0.900 (0.825, 0.981) |
|  | Days of antibiotic use in past 3 months | 5.167 (4.724, 5.610) | 4.328 (4.090, 4.567) | -0.960 (-1.454, -0.465) | ---- |
|  | Episodes of antibiotic use in past 3 months | 1.004 (0.929, 1.079) | 0.854 (0.809, 0.898) | -0.174 (-0.260, -0.088) | ---- |
|  | Multiple episodes of antibiotic use | 0.246 (0.214, 0.279) | 0.187 (0.168, 0.206) | -0.069 (-0.107, -0.035) | 0.730 (0.626, 0.851) |
| Nutrition | Any antibiotic use in past month | 0.495 (0.459, 0.530) | 0.434 (0.397, 0.471) | -0.070 (-0.123, -0.017) | 0.860 (0.765, 0.967) |
|  | Any antibiotic use in past 3 months | 0.665 (0.618, 0.713) | 0.587 (0.549, 0.626) | -0.083 (-0.143, -0.027) | 0.875 (0.795, 0.962) |
|  | Days of antibiotic use in past 3 months | 5.167 (4.724, 5.610) | 4.381 (3.935, 4.827) | -0.873 (-1.463, -0.283) | ---- |
|  | Episodes of antibiotic use in past 3 months | 1.004 (0.929, 1.079) | 0.844 (0.770, 0.917) | -0.179 (-0.280, -0.077) | ---- |
|  | Multiple episodes of antibiotic use | 0.246 (0.214, 0.279) | 0.178 (0.149, 0.206) | -0.077 (-0.117, -0.038) | 0.692 (0.574, 0.834) |
| WASH | Any antibiotic use in past month | 0.495 (0.459, 0.530) | 0.442 (0.406, 0.479) | -0.066 (-0.115, -0.020) | 0.867 (0.780, 0.964) |
|  | Any antibiotic use in past 3 months | 0.665 (0.618, 0.713) | 0.611 (0.578, 0.645) | -0.066 (-0.127, -0.003) | 0.901 (0.820, 0.990) |
|  | Days of antibiotic use in past 3 months | 5.167 (4.724, 5.610) | 4.323 (4.022, 4.624) | -0.960 (-1.481, -0.438) | ---- |
|  | Episodes of antibiotic use in past 3 months | 1.004 (0.929, 1.079) | 0.865 (0.802, 0.928) | -0.165 (-0.262, -0.067) | ---- |
|  | Multiple episodes of antibiotic use | 0.246 (0.214, 0.279) | 0.197 (0.169, 0.225) | -0.060 (-0.100, -0.021) | 0.767 (0.644, 0.913) |
| Nutrition + WASH | Any antibiotic use in past month | 0.495 (0.459, 0.530) | 0.472 (0.434, 0.509) | -0.031 (-0.086, 0.020) | 0.940 (0.841, 1.050) |
|  | Any antibiotic use in past 3 months | 0.665 (0.618, 0.713) | 0.606 (0.567, 0.646) | -0.067 (-0.129, -0.011) | 0.900 (0.817, 0.990) |
|  | Days of antibiotic use in past 3 months | 5.167 (4.724, 5.610) | 4.334 (3.948, 4.720) | -0.958 (-1.538, -0.379) | ---- |
|  | Episodes of antibiotic use in past 3 months | 1.004 (0.929, 1.079) | 0.842 (0.776, 0.909) | -0.184 (-0.281, -0.086) | ---- |
|  | Multiple episodes of antibiotic use | 0.246 (0.214, 0.279) | 0.177 (0.152, 0.202) | -0.076 (-0.118, -0.034) | 0.691 (0.569, 0.839) |

Point estimates and 95% confidence intervals (CIs) for the total effect of any WASH or nutrition intervention on antibiotic use outcomes. For categorical outcomes (any antibiotic use, multiple episodes of antibiotic use), the prevalence difference and prevalence ratio are reported. For continuous outcomes (days of antibiotic use, episodes of antibiotic use), only the mean difference is reported. Antibiotic use is reported by a caregiver under either a 1- or 3-month lookback period at 14 and 28 months.

# Table S6: Intervention-Mediator Effects

| **Intervention Group** | **Mediator** | **Prevalence/Mean in Control Arm** | **Prevalence/Mean in Intervention Arm** | **Mean Difference (95% CI)** | **Prevalence Ratio (95% CI)** |
| --- | --- | --- | --- | --- | --- |
| Pooled Intervention | Diarrhea, Fever, ARI, or Enteric Virus | 0.748 (0.697, 0.798) | 0.647 (0.616, 0.678) | ---- | 0.862 (0.785, 0.946) |
|  | Diarrhea, Fever, or ARI | 0.422 (0.390, 0.454) | 0.397 (0.378, 0.417) | ---- | 0.913 (0.833, 1.001) |
|  | Diarrhea | 0.116 (0.092, 0.140) | 0.083 (0.069, 0.097) | ---- | 0.671 (0.508, 0.886) |
|  | ARI | 0.217 (0.183, 0.250) | 0.188 (0.172, 0.204) | ---- | 0.835 (0.702, 0.994) |
|  | ARI with Fever | 0.131 (0.107, 0.155) | 0.100 (0.088, 0.112) | ---- | 0.739 (0.604, 0.906) |
|  | Difficulty Breathing | 0.040 (0.026, 0.054) | 0.029 (0.022, 0.037) | ---- | 0.706 (0.449, 1.110) |
|  | Fever (Past 7 Days) | 0.288 (0.254, 0.323) | 0.271 (0.253, 0.290) | ---- | 0.925 (0.819, 1.044) |
|  | Fever (Past 14 Days) | 0.443 (0.391, 0.495) | 0.421 (0.388, 0.453) | ---- | 0.936 (0.810, 1.081) |
|  | Any Enteric Virus | 0.495 (0.423, 0.568) | 0.306 (0.271, 0.341) | ---- | 0.595 (0.500, 0.709) |
|  | Any Enteric Virus with Diarrhea | 0.063 (0.041, 0.084) | 0.027 (0.021, 0.033) | ---- | 0.351 (0.242, 0.510) |
|  | Number of Enteric Viruses | 0.568 (0.470, 0.667) | 0.368 (0.319, 0.417) | -0.215 (-0.321, -0.110) | ---- |
|  | Adenovirus 40/41 | 0.103 (0.047, 0.159) | 0.070 (0.046, 0.093) | ---- | 0.596 (0.350, 1.013) |
|  | Norovirus GII | 0.191 (0.146, 0.237) | 0.133 (0.107, 0.160) | ---- | 0.669 (0.481, 0.930) |
|  | Sapovirus | 0.185 (0.134, 0.237) | 0.100 (0.079, 0.121) | ---- | 0.528 (0.369, 0.754) |
| Pooled Nutrition and Nutrition + WASH | Diarrhea, Fever, ARI, or Enteric Virus | 0.748 (0.697, 0.798) | 0.654 (0.617, 0.691) | ---- | 0.869 (0.790, 0.956) |
|  | Diarrhea, Fever, or ARI | 0.422 (0.390, 0.454) | 0.384 (0.359, 0.409) | ---- | 0.883 (0.799, 0.976) |
|  | Diarrhea | 0.116 (0.092, 0.140) | 0.086 (0.068, 0.103) | ---- | 0.689 (0.514, 0.924) |
|  | ARI | 0.217 (0.183, 0.250) | 0.180 (0.160, 0.199) | ---- | 0.803 (0.656, 0.982) |
|  | ARI with Fever | 0.131 (0.107, 0.155) | 0.097 (0.083, 0.112) | ---- | 0.726 (0.573, 0.921) |
|  | Difficulty Breathing | 0.040 (0.026, 0.054) | 0.030 (0.020, 0.039) | ---- | 0.719 (0.452, 1.143) |
|  | Fever (Past 7 Days) | 0.288 (0.254, 0.323) | 0.264 (0.241, 0.286) | ---- | 0.899 (0.790, 1.024) |
|  | Fever (Past 14 Days) | 0.443 (0.391, 0.495) | 0.409 (0.371, 0.447) | ---- | 0.900 (0.778, 1.041) |
|  | Any Enteric Virus | 0.495 (0.423, 0.568) | 0.326 (0.282, 0.371) | ---- | 0.643 (0.533, 0.776) |
|  | Any Enteric Virus with Diarrhea | 0.063 (0.041, 0.084) | 0.028 (0.021, 0.035) | ---- | 0.369 (0.247, 0.549) |
|  | Number of Enteric Viruses | 0.568 (0.470, 0.667) | 0.399 (0.338, 0.461) | -0.179 (-0.287, -0.070) | ---- |
|  | Adenovirus 40/41 | 0.103 (0.047, 0.159) | 0.076 (0.047, 0.105) | ---- | 0.661 (0.389, 1.126) |
|  | Norovirus GII | 0.191 (0.146, 0.237) | 0.156 (0.121, 0.192) | ---- | 0.799 (0.570, 1.119) |
|  | Sapovirus | 0.185 (0.134, 0.237) | 0.108 (0.084, 0.133) | ---- | 0.579 (0.392, 0.855) |
| Pooled WASH and Nutrition + WASH | Diarrhea, Fever, ARI, or Enteric Virus | 0.748 (0.697, 0.798) | 0.638 (0.601, 0.675) | ---- | 0.849 (0.765, 0.943) |
|  | Diarrhea, Fever, or ARI | 0.422 (0.390, 0.454) | 0.389 (0.366, 0.412) | ---- | 0.894 (0.812, 0.985) |
|  | Diarrhea | 0.116 (0.092, 0.140) | 0.075 (0.059, 0.091) | ---- | 0.605 (0.441, 0.829) |
|  | ARI | 0.217 (0.183, 0.250) | 0.185 (0.164, 0.205) | ---- | 0.818 (0.690, 0.968) |
|  | ARI with Fever | 0.131 (0.107, 0.155) | 0.101 (0.086, 0.116) | ---- | 0.744 (0.605, 0.915) |
|  | Difficulty Breathing | 0.040 (0.026, 0.054) | 0.027 (0.018, 0.036) | ---- | 0.649 (0.393, 1.070) |
|  | Fever (Past 7 Days) | 0.288 (0.254, 0.323) | 0.272 (0.248, 0.296) | ---- | 0.927 (0.814, 1.055) |
|  | Fever (Past 14 Days) | 0.443 (0.391, 0.495) | 0.425 (0.384, 0.466) | ---- | 0.964 (0.827, 1.124) |
|  | Any Enteric Virus | 0.495 (0.423, 0.568) | 0.294 (0.256, 0.333) | ---- | 0.571 (0.469, 0.696) |
|  | Any Enteric Virus with Diarrhea | 0.063 (0.041, 0.084) | 0.024 (0.016, 0.031) | ---- | 0.302 (0.193, 0.474) |
|  | Number of Enteric Viruses | 0.568 (0.470, 0.667) | 0.349 (0.296, 0.403) | -0.240 (-0.356, -0.125) | ---- |
|  | Adenovirus 40/41 | 0.103 (0.047, 0.159) | 0.063 (0.040, 0.086) | ---- | 0.508 (0.277, 0.934) |
|  | Norovirus GII | 0.191 (0.146, 0.237) | 0.121 (0.093, 0.149) | ---- | 0.610 (0.423, 0.879) |
|  | Sapovirus | 0.185 (0.134, 0.237) | 0.100 (0.074, 0.127) | ---- | 0.528 (0.364, 0.766) |
| Nutrition | Diarrhea, Fever, ARI, or Enteric Virus | 0.748 (0.697, 0.798) | 0.665 (0.612, 0.717) | ---- | 0.882 (0.794, 0.979) |
|  | Diarrhea, Fever, or ARI | 0.422 (0.390, 0.454) | 0.415 (0.381, 0.448) | ---- | 0.955 (0.853, 1.070) |
|  | Diarrhea | 0.116 (0.092, 0.140) | 0.101 (0.074, 0.128) | ---- | 0.807 (0.581, 1.121) |
|  | ARI | 0.217 (0.183, 0.250) | 0.195 (0.170, 0.220) | ---- | 0.877 (0.696, 1.105) |
|  | ARI with Fever | 0.131 (0.107, 0.155) | 0.098 (0.079, 0.117) | ---- | 0.733 (0.555, 0.966) |
|  | Difficulty Breathing | 0.040 (0.026, 0.054) | 0.034 (0.021, 0.046) | ---- | 0.824 (0.481, 1.411) |
|  | Fever (Past 7 Days) | 0.288 (0.254, 0.323) | 0.271 (0.238, 0.303) | ---- | 0.922 (0.785, 1.082) |
|  | Fever (Past 14 Days) | 0.443 (0.391, 0.495) | 0.411 (0.363, 0.460) | ---- | 0.899 (0.763, 1.059) |
|  | Any Enteric Virus | 0.495 (0.423, 0.568) | 0.330 (0.261, 0.398) | ---- | 0.642 (0.507, 0.814) |
|  | Any Enteric Virus with Diarrhea | 0.063 (0.041, 0.084) | 0.034 (0.022, 0.046) | ---- | 0.451 (0.289, 0.706) |
|  | Number of Enteric Viruses | 0.568 (0.470, 0.667) | 0.406 (0.315, 0.497) | -0.176 (-0.299, -0.052) | ---- |
|  | Adenovirus 40/41 | 0.103 (0.047, 0.159) | 0.082 (0.043, 0.122) | ---- | 0.736 (0.403, 1.342) |
|  | Norovirus GII | 0.191 (0.146, 0.237) | 0.159 (0.110, 0.208) | ---- | 0.806 (0.543, 1.196) |
|  | Sapovirus | 0.185 (0.134, 0.237) | 0.099 (0.062, 0.137) | ---- | 0.507 (0.298, 0.864) |
| WASH | Diarrhea, Fever, ARI, or Enteric Virus | 0.748 (0.697, 0.798) | 0.633 (0.585, 0.682) | ---- | 0.838 (0.745, 0.943) |
|  | Diarrhea, Fever, or ARI | 0.422 (0.390, 0.454) | 0.423 (0.395, 0.451) | ---- | 0.972 (0.876, 1.078) |
|  | Diarrhea | 0.116 (0.092, 0.140) | 0.079 (0.060, 0.098) | ---- | 0.633 (0.450, 0.889) |
|  | ARI | 0.217 (0.183, 0.250) | 0.204 (0.179, 0.229) | ---- | 0.903 (0.766, 1.066) |
|  | ARI with Fever | 0.131 (0.107, 0.155) | 0.104 (0.083, 0.126) | ---- | 0.768 (0.609, 0.968) |
|  | Difficulty Breathing | 0.040 (0.026, 0.054) | 0.028 (0.016, 0.040) | ---- | 0.679 (0.372, 1.238) |
|  | Fever (Past 7 Days) | 0.288 (0.254, 0.323) | 0.287 (0.256, 0.317) | ---- | 0.975 (0.842, 1.128) |
|  | Fever (Past 14 Days) | 0.443 (0.391, 0.495) | 0.443 (0.390, 0.496) | ---- | 1.010 (0.851, 1.200) |
|  | Any Enteric Virus | 0.495 (0.423, 0.568) | 0.266 (0.219, 0.314) | ---- | 0.502 (0.396, 0.636) |
|  | Any Enteric Virus with Diarrhea | 0.063 (0.041, 0.084) | 0.026 (0.016, 0.037) | ---- | 0.318 (0.190, 0.531) |
|  | Number of Enteric Viruses | 0.568 (0.470, 0.667) | 0.307 (0.244, 0.370) | -0.296 (-0.418, -0.174) | ---- |
|  | Adenovirus 40/41 | 0.103 (0.047, 0.159) | 0.057 (0.031, 0.083) | ---- | 0.450 (0.219, 0.923) |
|  | Norovirus GII | 0.191 (0.146, 0.237) | 0.090 (0.061, 0.119) | ---- | 0.437 (0.288, 0.664) |
|  | Sapovirus | 0.185 (0.134, 0.237) | 0.084 (0.051, 0.118) | ---- | 0.418 (0.261, 0.669) |
| Nutrition + WASH | Diarrhea, Fever, ARI, or Enteric Virus | 0.748 (0.697, 0.798) | 0.643 (0.591, 0.696) | ---- | 0.852 (0.758, 0.958) |
|  | Diarrhea, Fever, or ARI | 0.422 (0.390, 0.454) | 0.355 (0.318, 0.392) | ---- | 0.817 (0.719, 0.928) |
|  | Diarrhea | 0.116 (0.092, 0.140) | 0.071 (0.049, 0.093) | ---- | 0.575 (0.392, 0.844) |
|  | ARI | 0.217 (0.183, 0.250) | 0.165 (0.135, 0.195) | ---- | 0.737 (0.585, 0.928) |
|  | ARI with Fever | 0.131 (0.107, 0.155) | 0.097 (0.077, 0.117) | ---- | 0.724 (0.553, 0.948) |
|  | Difficulty Breathing | 0.040 (0.026, 0.054) | 0.026 (0.012, 0.039) | ---- | 0.621 (0.340, 1.135) |
|  | Fever (Past 7 Days) | 0.288 (0.254, 0.323) | 0.257 (0.224, 0.289) | ---- | 0.880 (0.754, 1.026) |
|  | Fever (Past 14 Days) | 0.443 (0.391, 0.495) | 0.406 (0.351, 0.462) | ---- | 0.916 (0.773, 1.085) |
|  | Any Enteric Virus | 0.495 (0.423, 0.568) | 0.323 (0.264, 0.383) | ---- | 0.640 (0.505, 0.809) |
|  | Any Enteric Virus with Diarrhea | 0.063 (0.041, 0.084) | 0.021 (0.011, 0.031) | ---- | 0.287 (0.162, 0.507) |
|  | Number of Enteric Viruses | 0.568 (0.470, 0.667) | 0.393 (0.312, 0.473) | -0.191 (-0.321, -0.062) | ---- |
|  | Adenovirus 40/41 | 0.103 (0.047, 0.159) | 0.070 (0.038, 0.102) | ---- | 0.565 (0.297, 1.076) |
|  | Norovirus GII | 0.191 (0.146, 0.237) | 0.153 (0.109, 0.197) | ---- | 0.795 (0.533, 1.186) |
|  | Sapovirus | 0.185 (0.134, 0.237) | 0.117 (0.079, 0.155) | ---- | 0.629 (0.407, 0.973) |

Point estimates and 95% confidence intervals for the effects of WASH and/or Nutrition interventions on potential mediators. For categorical mediators, the prevalence ratio is reported. For continuous mediators, the mean difference is reported. Diarrhea, ARI, ARI with Fever, and Fever are all reported by a caregiver under a 7-day lookback period at 14 and 28 months. Any Enteric Virus is the presence of adenovirus 40/41, norovirus GI, norovirus GII, sapovirus, rotavirus, or astrovirus in stool collected at 14 months, and Any Enteric Virus with Diarrhea is the presence of any enteric virus with caregiver reported diarrhea in the prior 7 days at 14 months.

# Table S7: Mediator-Outcome Effects

| **Mediator** | **Outcome** | **Prevalence/Mean in Absence of Mediator** | **Prevalence/Mean in Presence of Mediator** | **Mean Difference (95% CI)** | **Prevalence Ratio (95% CI)** |
| --- | --- | --- | --- | --- | --- |
| Diarrhea, Fever, ARI, or Enteric Virus | Any antibiotic use in past month | 0.433 (0.397, 0.469) | 0.621 (0.591, 0.652) | ---- | 1.429 (1.295, 1.577) |
| Diarrhea, Fever, or ARI | Any antibiotic use in past month | 0.395 (0.374, 0.416) | 0.559 (0.525, 0.593) | ---- | 1.296 (1.200, 1.399) |
| Diarrhea | Any antibiotic use in past month | 0.444 (0.424, 0.465) | 0.630 (0.569, 0.692) | ---- | 1.265 (1.135, 1.409) |
| ARI | Any antibiotic use in past month | 0.423 (0.402, 0.444) | 0.620 (0.575, 0.664) | ---- | 1.331 (1.225, 1.446) |
| ARI with Fever | Any antibiotic use in past month | 0.435 (0.414, 0.456) | 0.679 (0.623, 0.735) | ---- | 1.396 (1.265, 1.541) |
| Difficulty Breathing | Any antibiotic use in past month | 0.454 (0.433, 0.474) | 0.695 (0.595, 0.794) | ---- | 1.492 (1.286, 1.731) |
| Fever (Past 7 Days) | Any antibiotic use in past month | 0.422 (0.401, 0.443) | 0.564 (0.527, 0.602) | ---- | 1.250 (1.154, 1.354) |
| Fever (Past 14 Days) | Any antibiotic use in past month | 0.458 (0.427, 0.490) | 0.677 (0.635, 0.719) | ---- | 1.441 (1.302, 1.595) |
| Any Enteric Virus | Any antibiotic use in past month | 0.528 (0.494, 0.562) | 0.618 (0.578, 0.658) | ---- | 1.153 (1.046, 1.270) |
|  | Any antibiotic use in past 3 months | 0.705 (0.672, 0.738) | 0.753 (0.714, 0.791) | ---- | 1.060 (0.985, 1.142) |
|  | Days of antibiotic use in past 3 months | 5.606 (5.260, 5.953) | 5.804 (5.276, 6.333) | 0.212 (-0.400, 0.824) | ---- |
|  | Episodes of antibiotic use in past 3 months | 1.085 (1.021, 1.149) | 1.170 (1.083, 1.257) | 0.074 (-0.032, 0.179) | ---- |
|  | Multiple episodes of antibiotic use | 0.271 (0.242, 0.300) | 0.308 (0.264, 0.353) | ---- | 1.090 (0.921, 1.289) |
| Any Enteric Virus with Diarrhea | Any antibiotic use in past month | 0.452 (0.431, 0.472) | 0.752 (0.667, 0.838) | ---- | 1.373 (1.189, 1.585) |
|  | Any antibiotic use in past 3 months | 0.610 (0.589, 0.632) | 0.829 (0.757, 0.900) | ---- | 1.179 (1.069, 1.301) |
|  | Days of antibiotic use in past 3 months | 4.485 (4.259, 4.711) | 6.625 (5.391, 7.859) | 1.425 (0.092, 2.759) | ---- |
|  | Episodes of antibiotic use in past 3 months | 0.874 (0.835, 0.914) | 1.343 (1.164, 1.522) | 0.276 (0.078, 0.475) | ---- |
|  | Multiple episodes of antibiotic use | 0.195 (0.177, 0.213) | 0.362 (0.273, 0.451) | ---- | 1.311 (0.991, 1.734) |
| Number of Enteric Viruses | Any antibiotic use in past month | 0.528 (0.494, 0.562) | 0.618 (0.578, 0.658) | ---- | 1.075 (0.999, 1.156) |
|  | Any antibiotic use in past 3 months | 0.705 (0.672, 0.738) | 0.753 (0.714, 0.791) | ---- | 1.021 (0.966, 1.080) |
|  | Days of antibiotic use in past 3 months | 5.606 (5.260, 5.953) | 5.804 (5.276, 6.333) | 0.010 (-0.463, 0.483) | ---- |
|  | Episodes of antibiotic use in past 3 months | 1.085 (1.021, 1.149) | 1.170 (1.083, 1.257) | 0.021 (-0.059, 0.102) | ---- |
|  | Multiple episodes of antibiotic use | 0.271 (0.242, 0.300) | 0.308 (0.264, 0.353) | ---- | 1.026 (0.899, 1.172) |
| Adenovirus 40/41 | Any antibiotic use in past month | 0.559 (0.531, 0.587) | 0.569 (0.476, 0.661) | ---- | 1.026 (0.851, 1.237) |
|  | Any antibiotic use in past 3 months | 0.721 (0.695, 0.748) | 0.725 (0.660, 0.789) | ---- | 1.016 (0.914, 1.129) |
|  | Days of antibiotic use in past 3 months | 5.695 (5.392, 5.998) | 5.444 (4.518, 6.370) | -0.106 (-1.169, 0.958) | ---- |
|  | Episodes of antibiotic use in past 3 months | 1.109 (1.058, 1.160) | 1.183 (0.991, 1.376) | 0.087 (-0.125, 0.299) | ---- |
|  | Multiple episodes of antibiotic use | 0.282 (0.257, 0.306) | 0.312 (0.202, 0.422) | ---- | 1.131 (0.783, 1.634) |
| Norovirus GII | Any antibiotic use in past month | 0.551 (0.522, 0.580) | 0.609 (0.542, 0.676) | ---- | 1.067 (0.932, 1.222) |
|  | Any antibiotic use in past 3 months | 0.719 (0.693, 0.746) | 0.734 (0.680, 0.789) | ---- | 1.005 (0.918, 1.101) |
|  | Days of antibiotic use in past 3 months | 5.666 (5.370, 5.963) | 5.728 (4.937, 6.519) | -0.061 (-0.908, 0.786) | ---- |
|  | Episodes of antibiotic use in past 3 months | 1.114 (1.061, 1.167) | 1.121 (1.000, 1.241) | -0.022 (-0.162, 0.117) | ---- |
|  | Multiple episodes of antibiotic use | 0.282 (0.257, 0.308) | 0.295 (0.229, 0.360) | ---- | 0.973 (0.769, 1.231) |
| Sapovirus | Any antibiotic use in past month | 0.552 (0.524, 0.580) | 0.613 (0.551, 0.675) | ---- | 1.106 (0.981, 1.248) |
|  | Any antibiotic use in past 3 months | 0.718 (0.692, 0.743) | 0.751 (0.688, 0.815) | ---- | 1.056 (0.963, 1.160) |
|  | Days of antibiotic use in past 3 months | 5.677 (5.364, 5.990) | 5.663 (4.763, 6.562) | 0.166 (-0.821, 1.153) | ---- |
|  | Episodes of antibiotic use in past 3 months | 1.111 (1.056, 1.167) | 1.142 (1.010, 1.274) | 0.042 (-0.106, 0.191) | ---- |
|  | Multiple episodes of antibiotic use | 0.282 (0.255, 0.309) | 0.302 (0.232, 0.371) | ---- | 1.068 (0.830, 1.373) |

Point estimates and 95% confidence intervals for the effects of mediators on antibiotic use. For categorical outcomes (any antibiotic use, multiple episodes of antibiotic use), the prevalence ratio is reported. For continuous outcomes (days of antibiotic use, episodes of antibiotic use), the mean difference is reported. Diarrhea, ARI, ARI with Fever, and Fever are all reported by a caregiver under a 7-day lookback period at 14 and 28 months. Any Enteric Virus is the presence of adenovirus 40/41, norovirus GI, norovirus GII, sapovirus, rotavirus, or astrovirus in stool collected at 14 months, and Any Enteric Virus with Diarrhea is the presence of any enteric virus with caregiver reported diarrhea in the prior 7 days at 14 months. Antibiotic use is reported by a caregiver under either a 1- or 3-month lookback period at 14 and 28 months.

# Table S8: Mediated Effects

| **Intervention Group** | **Mediator** | **Outcome** | **Total Natural Indirect Effect (Prevalence Difference)** | **Total Natural Indirect Effect (Prevalence Ratio)** |
| --- | --- | --- | --- | --- |
| Pooled Intervention | Diarrhea, Fever, ARI, or Enteric Virus | Any antibiotic use in past month | -0.021 (-0.045, 0.003) | 1.058 (1.014, 1.115) |
|  | Diarrhea, Fever, or ARI | Any antibiotic use in past month | -0.006 (-0.014, 0.002) | 1.021 (1.004, 1.043) |
|  | Diarrhea | Any antibiotic use in past month | -0.006 (-0.012, -0.001) | 0.997 (0.988, 1.005) |
|  | ARI | Any antibiotic use in past month | -0.006 (-0.014, 0.001) | 1.006 (0.994, 1.022) |
|  | ARI with Fever | Any antibiotic use in past month | -0.007 (-0.015, -0.001) | 1.002 (0.992, 1.013) |
|  | Difficulty Breathing | Any antibiotic use in past month | -0.003 (-0.008, 0.002) | 1.001 (0.993, 1.009) |
|  | Fever (Past 7 Days) | Any antibiotic use in past month | -0.003 (-0.009, 0.003) | 1.011 (0.999, 1.025) |
|  | Fever (Past 14 Days) | Any antibiotic use in past month | -0.008 (-0.029, 0.012) | 1.058 (1.018, 1.109) |
|  | Any Enteric Virus | Any antibiotic use in past month | -0.015 (-0.030, -0.004) | 0.984 (0.968, 0.997) |
|  | Any Enteric Virus with Diarrhea | Any antibiotic use in past month | -0.011 (-0.020, -0.004) | 0.989 (0.980, 0.996) |
|  |  | Any antibiotic use in past 3 months | -0.007 (-0.015, -0.002) | 0.992 (0.986, 0.997) |
|  |  | Days of antibiotic use in past 3 months | -0.055 (-0.142, 0.014) | ---- |
|  |  | Episodes of antibiotic use in past 3 months | -0.015 (-0.033, -0.003) | ---- |
|  |  | Multiple episodes of antibiotic use | -0.004 (-0.011, 0.001) | 0.992 (0.980, 1.005) |
|  | Adenovirus 40/41 | Multiple episodes of antibiotic use | -0.002 (-0.015, 0.005) | 0.998 (0.976, 1.026) |
|  | Sapovirus | Any antibiotic use in past month | -0.004 (-0.011, 0.001) | 0.996 (0.985, 1.003) |
| Pooled Nutrition and Nutrition + WASH | Diarrhea, Fever, ARI, or Enteric Virus | Any antibiotic use in past month | -0.011 (-0.027, 0.003) | 1.021 (0.994, 1.068) |
|  | Diarrhea, Fever, or ARI | Any antibiotic use in past month | -0.005 (-0.013, 0.001) | 1.009 (0.995, 1.029) |
|  | Diarrhea | Any antibiotic use in past month | -0.005 (-0.012, 0.000) | 0.997 (0.987, 1.006) |
|  | ARI | Any antibiotic use in past month | -0.004 (-0.011, 0.002) | 1.002 (0.992, 1.016) |
|  | ARI with Fever | Any antibiotic use in past month | -0.005 (-0.013, 0.001) | 1.001 (0.991, 1.013) |
|  | Difficulty Breathing | Any antibiotic use in past month | -0.003 (-0.008, 0.002) | 1.001 (0.994, 1.010) |
|  | Fever (Past 7 Days) | Any antibiotic use in past month | -0.003 (-0.008, 0.002) | 1.005 (0.995, 1.018) |
|  | Fever (Past 14 Days) | Any antibiotic use in past month | -0.010 (-0.031, 0.010) | 1.036 (1.002, 1.080) |
|  | Any Enteric Virus | Any antibiotic use in past month | -0.012 (-0.028, -0.001) | 0.988 (0.972, 1.001) |
|  | Any Enteric Virus with Diarrhea | Any antibiotic use in past month | -0.008 (-0.018, -0.003) | 0.992 (0.983, 0.999) |
|  |  | Any antibiotic use in past 3 months | -0.007 (-0.015, -0.002) | 0.992 (0.984, 0.998) |
|  |  | Days of antibiotic use in past 3 months | -0.069 (-0.175, 0.022) | ---- |
|  |  | Episodes of antibiotic use in past 3 months | -0.010 (-0.026, 0.002) | ---- |
|  |  | Multiple episodes of antibiotic use | -0.002 (-0.009, 0.004) | 0.997 (0.982, 1.018) |
|  | Adenovirus 40/41 | Multiple episodes of antibiotic use | 0.000 (-0.011, 0.008) | 1.005 (0.978, 1.047) |
|  | Sapovirus | Any antibiotic use in past month | -0.003 (-0.010, 0.003) | 0.997 (0.986, 1.006) |
| Pooled WASH and Nutrition + WASH | Diarrhea, Fever, ARI, or Enteric Virus | Any antibiotic use in past month | -0.029 (-0.070, 0.001) | 1.079 (1.015, 1.173) |
|  | Diarrhea, Fever, or ARI | Any antibiotic use in past month | -0.010 (-0.023, 0.001) | 1.021 (0.999, 1.051) |
|  | Diarrhea | Any antibiotic use in past month | -0.009 (-0.018, -0.002) | 0.992 (0.981, 1.002) |
|  | ARI | Any antibiotic use in past month | -0.008 (-0.019, 0.000) | 1.004 (0.989, 1.022) |
|  | ARI with Fever | Any antibiotic use in past month | -0.007 (-0.016, -0.001) | 1.000 (0.988, 1.013) |
|  | Difficulty Breathing | Any antibiotic use in past month | -0.004 (-0.012, 0.000) | 0.998 (0.990, 1.008) |
|  | Fever (Past 7 Days) | Any antibiotic use in past month | -0.005 (-0.014, 0.002) | 1.012 (0.997, 1.033) |
|  | Fever (Past 14 Days) | Any antibiotic use in past month | -0.003 (-0.028, 0.019) | 1.065 (1.019, 1.123) |
|  | Any Enteric Virus | Any antibiotic use in past month | -0.018 (-0.037, -0.005) | 0.981 (0.961, 0.997) |
|  | Any Enteric Virus with Diarrhea | Any antibiotic use in past month | -0.013 (-0.025, -0.004) | 0.986 (0.974, 0.995) |
|  | Any Enteric Virus with Diarrhea | Any antibiotic use in past 3 months | -0.008 (-0.016, -0.001) | 0.992 (0.983, 0.998) |
|  |  | Days of antibiotic use in past 3 months | -0.092 (-0.217, 0.008) | ---- |
|  |  | Episodes of antibiotic use in past 3 months | -0.020 (-0.042, -0.004) | ---- |
|  |  | Multiple episodes of antibiotic use | -0.006 (-0.015, 0.001) | 0.989 (0.974, 1.003) |
|  | Adenovirus 40/41 | Multiple episodes of antibiotic use | -0.004 (-0.026, 0.009) | 0.998 (0.961, 1.048) |
|  | Sapovirus | Any antibiotic use in past month | -0.005 (-0.015, 0.001) | 0.995 (0.984, 1.003) |
| Nutrition | Diarrhea, Fever, ARI, or Enteric Virus | Any antibiotic use in past month | -0.010 (-0.029, 0.007) | 1.022 (0.990, 1.077) |
|  | Diarrhea, Fever, or ARI | Any antibiotic use in past month | -0.002 (-0.009, 0.005) | 1.015 (0.998, 1.041) |
|  | Diarrhea | Any antibiotic use in past month | -0.003 (-0.010, 0.002) | 1.001 (0.992, 1.012) |
|  | ARI | Any antibiotic use in past month | -0.002 (-0.009, 0.004) | 1.005 (0.994, 1.022) |
|  | ARI with Fever | Any antibiotic use in past month | -0.004 (-0.013, 0.002) | 1.001 (0.990, 1.014) |
|  | Difficulty Breathing | Any antibiotic use in past month | -0.001 (-0.005, 0.003) | 1.001 (0.994, 1.012) |
|  | Fever (Past 7 Days) | Any antibiotic use in past month | -0.002 (-0.009, 0.004) | 1.008 (0.996, 1.027) |
|  | Fever (Past 14 Days) | Any antibiotic use in past month | -0.009 (-0.035, 0.012) | 1.035 (0.996, 1.087) |
|  | Any Enteric Virus | Any antibiotic use in past month | -0.012 (-0.032, 0.000) | 0.988 (0.968, 1.006) |
|  | Any Enteric Virus with Diarrhea | Any antibiotic use in past month | -0.007 (-0.016, -0.001) | 0.994 (0.984, 1.002) |
|  |  | Any antibiotic use in past 3 months | -0.007 (-0.015, -0.001) | 0.992 (0.984, 0.998) |
|  |  | Days of antibiotic use in past 3 months | -0.072 (-0.203, 0.009) | ---- |
|  |  | Episodes of antibiotic use in past 3 months | -0.008 (-0.024, 0.001) | ---- |
|  |  | Multiple episodes of antibiotic use | -0.002 (-0.008, 0.002) | 0.996 (0.983, 1.011) |
|  | Adenovirus 40/41 | Multiple episodes of antibiotic use | -0.002 (-0.014, 0.004) | 1.000 (0.979, 1.034) |
|  | Sapovirus | Any antibiotic use in past month | -0.002 (-0.012, 0.006) | 0.997 (0.981, 1.012) |
| WASH | Diarrhea, Fever, ARI, or Enteric Virus | Any antibiotic use in past month | -0.044 (-0.103, 0.002) | 1.105 (1.012, 1.249) |
|  | Diarrhea, Fever, or ARI | Any antibiotic use in past month | -0.004 (-0.018, 0.010) | 1.042 (1.008, 1.085) |
|  | Diarrhea | Any antibiotic use in past month | -0.007 (-0.017, 0.000) | 0.995 (0.984, 1.007) |
|  | ARI | Any antibiotic use in past month | -0.005 (-0.016, 0.004) | 1.007 (0.992, 1.029) |
|  | ARI with Fever | Any antibiotic use in past month | -0.007 (-0.018, 0.001) | 1.000 (0.987, 1.017) |
|  | Difficulty Breathing | Any antibiotic use in past month | -0.002 (-0.008, 0.001) | 0.998 (0.991, 1.007) |
|  | Fever (Past 7 Days) | Any antibiotic use in past month | -0.003 (-0.015, 0.007) | 1.022 (1.000, 1.050) |
|  | Fever (Past 14 Days) | Any antibiotic use in past month | 0.002 (-0.029, 0.032) | 1.095 (1.028, 1.180) |
|  | Any Enteric Virus | Any antibiotic use in past month | -0.024 (-0.053, -0.002) | 0.972 (0.946, 0.995) |
|  | Any Enteric Virus with Diarrhea | Any antibiotic use in past month | -0.011 (-0.025, -0.002) | 0.988 (0.974, 0.996) |
|  |  | Any antibiotic use in past 3 months | -0.008 (-0.017, -0.001) | 0.992 (0.983, 0.998) |
|  |  | Days of antibiotic use in past 3 months | -0.105 (-0.255, 0.003) | ---- |
|  |  | Episodes of antibiotic use in past 3 months | -0.023 (-0.051, -0.005) | ---- |
|  |  | Multiple episodes of antibiotic use | -0.007 (-0.015, -0.001) | 0.988 (0.975, 0.998) |
|  | Adenovirus 40/41 | Multiple episodes of antibiotic use | -0.005 (-0.020, 0.003) | 0.993 (0.963, 1.018) |
|  | Sapovirus | Any antibiotic use in past month | -0.007 (-0.022, 0.003) | 0.990 (0.972, 1.006) |
| Nutrition + WASH | Diarrhea, Fever, ARI, or Enteric Virus | Any antibiotic use in past month | -0.016 (-0.046, 0.006) | 1.027 (0.988, 1.093) |
|  | Diarrhea, Fever, or ARI | Any antibiotic use in past month | -0.010 (-0.022, -0.001) | 1.005 (0.988, 1.030) |
|  | Diarrhea | Any antibiotic use in past month | -0.008 (-0.018, -0.001) | 0.992 (0.981, 1.004) |
|  | ARI | Any antibiotic use in past month | -0.005 (-0.013, 0.000) | 0.998 (0.989, 1.009) |
|  | ARI with Fever | Any antibiotic use in past month | -0.004 (-0.012, 0.002) | 1.000 (0.990, 1.011) |
|  | Difficulty Breathing | Any antibiotic use in past month | -0.004 (-0.012, 0.001) | 0.999 (0.991, 1.010) |
|  | Fever (Past 7 Days) | Any antibiotic use in past month | -0.004 (-0.013, 0.003) | 1.007 (0.994, 1.024) |
|  | Fever (Past 14 Days) | Any antibiotic use in past month | -0.008 (-0.032, 0.015) | 1.036 (0.997, 1.091) |
|  | Any Enteric Virus | Any antibiotic use in past month | -0.014 (-0.037, -0.001) | 0.987 (0.964, 1.005) |
|  | Any Enteric Virus with Diarrhea | Any antibiotic use in past month | -0.008 (-0.019, -0.002) | 0.991 (0.981, 0.999) |
|  |  | Any antibiotic use in past 3 months | -0.008 (-0.019, -0.002) | 0.991 (0.982, 0.999) |
|  |  | Days of antibiotic use in past 3 months | -0.101 (-0.249, 0.023) | ---- |
|  |  | Episodes of antibiotic use in past 3 months | -0.017 (-0.041, 0.002) | ---- |
|  |  | Multiple episodes of antibiotic use | -0.002 (-0.011, 0.004) | 0.998 (0.978, 1.028) |
|  | Adenovirus 40/41 | Multiple episodes of antibiotic use | 0.000 (-0.017, 0.013) | 1.012 (0.971, 1.087) |
|  | Sapovirus | Any antibiotic use in past month | -0.003 (-0.015, 0.003) | 0.997 (0.983, 1.011) |

Point estimates and 95% confidence intervals for the total natural indirect effects of WASH and/or Nutrition intervention on antibiotic use. For categorical outcomes (any antibiotic use, multiple episodes of antibiotic use), the prevalence ratio and prevalence difference are reported. For continuous outcomes (days of antibiotic use, episodes of antibiotic use), the mean difference is reported. Diarrhea, ARI, ARI with Fever, and Fever are all reported by a caregiver under a 7-day lookback period at 14 and 28 months. Any Enteric Virus is the presence of adenovirus 40/41, norovirus GI, norovirus GII, sapovirus, rotavirus, or astrovirus in stool collected at 14 months, and Any Enteric Virus with Diarrhea is the presence of any enteric virus with caregiver reported diarrhea in the prior 7 days at 14 months. Antibiotic use is reported by a caregiver under either a 1- or 3-month lookback period at 14 and 28 months.

# Table S9: Mediated Effects, with Intervention-Mediator Interactions

| **Intervention Group** | **Mediator** | **Outcome** | **Pure Natural Indirect Effect** | **Total Natural Indirect Effect** |
| --- | --- | --- | --- | --- |
| Pooled Intervention | Diarrhea, Fever, ARI, or Enteric Virus | Any antibiotic use in past month | -0.014 (-0.042, 0.001) | -0.024 (-0.054, 0.000) |
|  | Diarrhea, Fever, or ARI | Any antibiotic use in past month | -0.006 (-0.017, 0.002) | -0.006 (-0.016, 0.002) |
|  | Diarrhea | Any antibiotic use in past month | -0.006 (-0.014, -0.001) | -0.006 (-0.013, -0.001) |
|  | ARI | Any antibiotic use in past month | -0.003 (-0.010, 0.001) | -0.007 (-0.017, 0.001) |
|  | ARI with Fever | Any antibiotic use in past month | -0.004 (-0.011, 0.000) | -0.008 (-0.018, -0.001) |
|  | Difficulty Breathing | Any antibiotic use in past month | 0.000 (-0.005, 0.003) | -0.004 (-0.010, 0.002) |
|  | Fever (Past 7 Days) | Any antibiotic use in past month | -0.003 (-0.012, 0.004) | -0.003 (-0.009, 0.003) |
|  | Fever (Past 14 Days) | Any antibiotic use in past month | -0.007 (-0.029, 0.011) | -0.008 (-0.034, 0.014) |
|  | Any Enteric Virus | Any antibiotic use in past month | -0.013 (-0.041, 0.008) | -0.016 (-0.037, -0.002) |
|  | Any Enteric Virus with Diarrhea | Any antibiotic use in past month | -0.009 (-0.020, -0.001) | -0.013 (-0.025, -0.005) |
|  |  | Any antibiotic use in past 3 months | -0.009 (-0.022, 0.000) | -0.007 (-0.016, 0.000) |
|  |  | Days of antibiotic use in past 3 months | -0.121 (-0.311, 0.006) | -0.012 (-0.094, 0.062) |
|  |  | Episodes of antibiotic use in past 3 months | -0.023 (-0.054, 0.001) | -0.010 (-0.028, 0.005) |
|  |  | Multiple episodes of antibiotic use | -0.004 (-0.017, 0.004) | -0.004 (-0.015, 0.002) |
|  | Adenovirus 40/41 | Multiple episodes of antibiotic use | -0.004 (-0.029, 0.010) | -0.002 (-0.012, 0.006) |
|  | Sapovirus | Any antibiotic use in past month | -0.005 (-0.020, 0.005) | -0.004 (-0.013, 0.002) |
| Pooled Nutrition and Nutrition + WASH | Diarrhea, Fever, ARI, or Enteric Virus | Any antibiotic use in past month | -0.013 (-0.043, 0.004) | -0.011 (-0.029, 0.004) |
|  | Diarrhea, Fever, or ARI | Any antibiotic use in past month | -0.007 (-0.019, 0.001) | -0.005 (-0.014, 0.001) |
|  | Diarrhea | Any antibiotic use in past month | -0.006 (-0.015, 0.000) | -0.005 (-0.013, 0.000) |
|  | ARI | Any antibiotic use in past month | -0.002 (-0.008, 0.002) | -0.006 (-0.016, 0.002) |
|  | ARI with Fever | Any antibiotic use in past month | -0.003 (-0.009, 0.001) | -0.007 (-0.017, 0.001) |
|  | Difficulty Breathing | Any antibiotic use in past month | 0.000 (-0.005, 0.003) | -0.004 (-0.013, 0.004) |
|  | Fever (Past 7 Days) | Any antibiotic use in past month | -0.004 (-0.013, 0.003) | -0.002 (-0.008, 0.002) |
|  | Fever (Past 14 Days) | Any antibiotic use in past month | -0.010 (-0.030, 0.009) | -0.010 (-0.032, 0.010) |
|  | Any Enteric Virus | Any antibiotic use in past month | -0.013 (-0.042, 0.005) | -0.012 (-0.032, 0.001) |
|  | Any Enteric Virus with Diarrhea | Any antibiotic use in past month | -0.006 (-0.017, 0.000) | -0.011 (-0.024, -0.003) |
|  |  | Any antibiotic use in past 3 months | -0.009 (-0.020, -0.001) | -0.007 (-0.020, 0.001) |
|  |  | Days of antibiotic use in past 3 months | -0.139 (-0.326, -0.001) | -0.001 (-0.120, 0.116) |
|  |  | Episodes of antibiotic use in past 3 months | -0.020 (-0.049, 0.000) | 0.000 (-0.017, 0.016) |
|  |  | Multiple episodes of antibiotic use | -0.006 (-0.020, 0.003) | 0.001 (-0.012, 0.008) |
|  | Adenovirus 40/41 | Multiple episodes of antibiotic use | -0.005 (-0.034, 0.008) | 0.002 (-0.006, 0.011) |
|  | Sapovirus | Any antibiotic use in past month | -0.004 (-0.020, 0.004) | -0.002 (-0.011, 0.004) |
| Pooled WASH and Nutrition + WASH | Diarrhea, Fever, ARI, or Enteric Virus | Any antibiotic use in past month | -0.016 (-0.048, 0.002) | -0.036 (-0.086, 0.002) |
|  | Diarrhea, Fever, or ARI | Any antibiotic use in past month | -0.008 (-0.021, 0.001) | -0.011 (-0.026, 0.001) |
|  | Diarrhea | Any antibiotic use in past month | -0.009 (-0.020, -0.001) | -0.010 (-0.021, -0.003) |
|  | ARI | Any antibiotic use in past month | -0.004 (-0.014, 0.001) | -0.011 (-0.026, 0.001) |
|  | ARI with Fever | Any antibiotic use in past month | -0.004 (-0.013, 0.000) | -0.010 (-0.020, -0.001) |
|  | Difficulty Breathing | Any antibiotic use in past month | -0.001 (-0.008, 0.003) | -0.007 (-0.016, 0.001) |
|  | Fever (Past 7 Days) | Any antibiotic use in past month | -0.005 (-0.016, 0.003) | -0.005 (-0.015, 0.003) |
|  | Fever (Past 14 Days) | Any antibiotic use in past month | -0.004 (-0.025, 0.016) | -0.005 (-0.033, 0.021) |
|  | Any Enteric Virus | Any antibiotic use in past month | -0.015 (-0.044, 0.008) | -0.021 (-0.048, -0.003) |
|  | Any Enteric Virus with Diarrhea | Any antibiotic use in past month | -0.012 (-0.028, 0.000) | -0.016 (-0.035, -0.004) |
|  | Any Enteric Virus with Diarrhea | Any antibiotic use in past 3 months | -0.008 (-0.021, 0.001) | -0.008 (-0.020, 0.001) |
|  |  | Days of antibiotic use in past 3 months | -0.128 (-0.314, 0.033) | -0.048 (-0.170, 0.058) |
|  |  | Episodes of antibiotic use in past 3 months | -0.022 (-0.054, 0.003) | -0.019 (-0.044, 0.000) |
|  |  | Multiple episodes of antibiotic use | -0.004 (-0.019, 0.005) | -0.010 (-0.029, 0.001) |
|  | Adenovirus 40/41 | Multiple episodes of antibiotic use | -0.005 (-0.038, 0.017) | -0.004 (-0.026, 0.010) |
|  | Sapovirus | Any antibiotic use in past month | -0.005 (-0.019, 0.005) | -0.005 (-0.017, 0.002) |
| Nutrition | Diarrhea, Fever, ARI, or Enteric Virus | Any antibiotic use in past month | -0.013 (-0.048, 0.009) | -0.009 (-0.032, 0.005) |
|  | Diarrhea, Fever, or ARI | Any antibiotic use in past month | -0.002 (-0.013, 0.007) | -0.001 (-0.009, 0.005) |
|  | Diarrhea | Any antibiotic use in past month | -0.003 (-0.012, 0.003) | -0.002 (-0.010, 0.003) |
|  | ARI | Any antibiotic use in past month | -0.001 (-0.006, 0.003) | -0.003 (-0.015, 0.007) |
|  | ARI with Fever | Any antibiotic use in past month | -0.003 (-0.009, 0.001) | -0.007 (-0.020, 0.003) |
|  | Difficulty Breathing | Any antibiotic use in past month | 0.000 (-0.004, 0.003) | -0.002 (-0.010, 0.006) |
|  | Fever (Past 7 Days) | Any antibiotic use in past month | -0.003 (-0.015, 0.006) | -0.001 (-0.007, 0.003) |
|  | Fever (Past 14 Days) | Any antibiotic use in past month | -0.008 (-0.028, 0.010) | -0.014 (-0.048, 0.013) |
|  | Any Enteric Virus | Any antibiotic use in past month | -0.014 (-0.046, 0.006) | -0.011 (-0.035, 0.006) |
|  | Any Enteric Virus with Diarrhea | Any antibiotic use in past month | -0.005 (-0.018, 0.001) | -0.012 (-0.028, -0.002) |
|  |  | Any antibiotic use in past 3 months | -0.008 (-0.019, -0.001) | -0.006 (-0.022, 0.004) |
|  |  | Days of antibiotic use in past 3 months | -0.136 (-0.349, -0.008) | 0.020 (-0.111, 0.153) |
|  |  | Episodes of antibiotic use in past 3 months | -0.014 (-0.034, 0.000) | 0.002 (-0.012, 0.017) |
|  |  | Multiple episodes of antibiotic use | -0.005 (-0.018, 0.002) | 0.000 (-0.013, 0.008) |
|  | Adenovirus 40/41 | Multiple episodes of antibiotic use | -0.006 (-0.034, 0.006) | -0.001 (-0.016, 0.009) |
|  | Sapovirus | Any antibiotic use in past month | -0.005 (-0.023, 0.006) | 0.001 (-0.012, 0.014) |
| WASH | Diarrhea, Fever, ARI, or Enteric Virus | Any antibiotic use in past month | -0.018 (-0.048, 0.001) | -0.078 (-0.211, 0.006) |
|  | Diarrhea, Fever, or ARI | Any antibiotic use in past month | -0.003 (-0.016, 0.007) | -0.007 (-0.029, 0.014) |
|  | Diarrhea | Any antibiotic use in past month | -0.006 (-0.017, 0.000) | -0.009 (-0.024, 0.000) |
|  | ARI | Any antibiotic use in past month | -0.002 (-0.010, 0.002) | -0.009 (-0.027, 0.005) |
|  | ARI with Fever | Any antibiotic use in past month | -0.004 (-0.012, 0.001) | -0.011 (-0.027, 0.001) |
|  | Difficulty Breathing | Any antibiotic use in past month | -0.001 (-0.008, 0.003) | -0.003 (-0.012, 0.001) |
|  | Fever (Past 7 Days) | Any antibiotic use in past month | -0.003 (-0.013, 0.006) | -0.004 (-0.020, 0.010) |
|  | Fever (Past 14 Days) | Any antibiotic use in past month | 0.001 (-0.026, 0.028) | 0.003 (-0.043, 0.050) |
|  | Any Enteric Virus | Any antibiotic use in past month | -0.018 (-0.060, 0.015) | -0.035 (-0.079, -0.004) |
|  | Any Enteric Virus with Diarrhea | Any antibiotic use in past month | -0.010 (-0.027, 0.001) | -0.017 (-0.041, -0.003) |
|  |  | Any antibiotic use in past 3 months | -0.008 (-0.021, 0.000) | -0.008 (-0.024, 0.002) |
|  |  | Days of antibiotic use in past 3 months | -0.122 (-0.311, 0.018) | -0.053 (-0.226, 0.086) |
|  |  | Episodes of antibiotic use in past 3 months | -0.021 (-0.052, 0.001) | -0.029 (-0.072, 0.001) |
|  |  | Multiple episodes of antibiotic use | -0.005 (-0.016, 0.003) | -0.016 (-0.047, -0.003) |
|  | Adenovirus 40/41 | Multiple episodes of antibiotic use | -0.002 (-0.025, 0.013) | -0.013 (-0.050, 0.002) |
|  | Sapovirus | Any antibiotic use in past month | -0.006 (-0.026, 0.010) | -0.013 (-0.045, 0.007) |
| Nutrition + WASH | Diarrhea, Fever, ARI, or Enteric Virus | Any antibiotic use in past month | -0.016 (-0.054, 0.005) | -0.018 (-0.056, 0.006) |
|  | Diarrhea, Fever, or ARI | Any antibiotic use in past month | -0.010 (-0.025, -0.001) | -0.010 (-0.025, 0.000) |
|  | Diarrhea | Any antibiotic use in past month | -0.008 (-0.019, -0.001) | -0.010 (-0.025, 0.000) |
|  | ARI | Any antibiotic use in past month | -0.003 (-0.011, 0.002) | -0.007 (-0.020, 0.000) |
|  | ARI with Fever | Any antibiotic use in past month | -0.003 (-0.010, 0.001) | -0.006 (-0.017, 0.003) |
|  | Difficulty Breathing | Any antibiotic use in past month | 0.000 (-0.006, 0.004) | -0.008 (-0.021, 0.003) |
|  | Fever (Past 7 Days) | Any antibiotic use in past month | -0.005 (-0.016, 0.003) | -0.004 (-0.015, 0.002) |
|  | Fever (Past 14 Days) | Any antibiotic use in past month | -0.007 (-0.032, 0.016) | -0.007 (-0.034, 0.016) |
|  | Any Enteric Virus | Any antibiotic use in past month | -0.015 (-0.046, 0.005) | -0.015 (-0.045, 0.004) |
|  | Any Enteric Virus with Diarrhea | Any antibiotic use in past month | -0.007 (-0.019, 0.000) | -0.012 (-0.033, -0.001) |
|  |  | Any antibiotic use in past 3 months | -0.009 (-0.021, 0.000) | -0.009 (-0.028, 0.004) |
|  |  | Days of antibiotic use in past 3 months | -0.126 (-0.327, 0.032) | -0.038 (-0.226, 0.148) |
|  |  | Episodes of antibiotic use in past 3 months | -0.022 (-0.050, 0.001) | -0.005 (-0.034, 0.022) |
|  |  | Multiple episodes of antibiotic use | -0.003 (-0.017, 0.006) | -0.002 (-0.025, 0.010) |
|  | Adenovirus 40/41 | Multiple episodes of antibiotic use | -0.005 (-0.037, 0.013) | 0.005 (-0.017, 0.023) |
|  | Sapovirus | Any antibiotic use in past month | -0.004 (-0.021, 0.005) | -0.004 (-0.020, 0.004) |

Point estimates and 95% confidence intervals for the total and pure natural indirect effects of WASH and/or Nutrition intervention on antibiotic use, computed only when the difference in total and pure natural indirect effects was greater than 1% or had a t-test p-value < 0.2 when outcome models included an intervention-mediator interaction term. For categorical outcomes (any antibiotic use, multiple episodes of antibiotic use), the prevalence difference is reported. For continuous outcomes (days of antibiotic use, episodes of antibiotic use), the mean difference is reported. Diarrhea, ARI, ARI with Fever, and Fever are all reported by a caregiver under a 7-day lookback period at 14 and 28 months. Any Enteric Virus is the presence of adenovirus 40/41, norovirus GI, norovirus GII, sapovirus, rotavirus, or astrovirus in stool collected at 14 months, and Any Enteric Virus with Diarrhea is the presence of any enteric virus with caregiver reported diarrhea in the prior 7 days at 14 months. Antibiotic use is reported by a caregiver under either a 1- or 3-month lookback period at 14 and 28 months.

# Table S10: Intervention-Mediator Effects, Pooled Intervention Group and Enteric Pathogen Carriage

| **Mediator** | **Prevalence/Mean in Control Arm** | **Prevalence/Mean in Intervention Arm** | **Prevalence Ratio (95% CI)** |
| --- | --- | --- | --- |
| ***BACTERIA*** | | | |
| Aeromonas | 0.064 (0.039, 0.089) | 0.059 (0.043, 0.076) | 0.812 (0.504, 1.308) |
| B.fragilis | 0.185 (0.149, 0.222) | 0.241 (0.211, 0.271) | 1.281 (1.000, 1.641) |
| C.difficile | 0.082 (0.052, 0.112) | 0.095 (0.076, 0.115) | 1.166 (0.748, 1.817) |
| Campylobacter | 0.413 (0.351, 0.475) | 0.487 (0.444, 0.529) | 1.126 (0.956, 1.326) |
| EAEC | 0.757 (0.700, 0.814) | 0.760 (0.731, 0.789) | 0.979 (0.897, 1.070) |
| EPEC (Any) | 0.538 (0.478, 0.598) | 0.520 (0.488, 0.552) | 0.938 (0.816, 1.078) |
| ETEC (Any) | 0.362 (0.295, 0.429) | 0.407 (0.362, 0.452) | 1.053 (0.860, 1.289) |
| Plesiomonas | 0.137 (0.091, 0.182) | 0.159 (0.129, 0.190) | 1.084 (0.778, 1.511) |
| Shigella/EIEC | 0.125 (0.087, 0.162) | 0.149 (0.119, 0.179) | 1.127 (0.850, 1.494) |
| STEC | 0.109 (0.073, 0.146) | 0.114 (0.092, 0.136) | 1.042 (0.706, 1.538) |
| ***PARASITES*** | | | |
| Any Parasite | 0.307 (0.242, 0.372) | 0.365 (0.329, 0.401) | 1.110 (0.888, 1.387) |
| Cryptosporidium | 0.079 (0.044, 0.114) | 0.133 (0.108, 0.159) | 1.448 (0.896, 2.341) |
| E.bieneusi | 0.106 (0.075, 0.137) | 0.101 (0.082, 0.120) | 0.854 (0.609, 1.196) |
| Giardia | 0.137 (0.085, 0.188) | 0.135 (0.112, 0.159) | 1.003 (0.663, 1.515) |
| ***VIRUSES*** | | | |
| Any Virus | 0.495 (0.423, 0.568) | 0.306 (0.271, 0.341) | 0.595 (0.500, 0.709) |
| Adenovirus 40/41 | 0.103 (0.047, 0.159) | 0.070 (0.046, 0.093) | 0.596 (0.350, 1.013) |
| Norovirus GII | 0.191 (0.146, 0.237) | 0.133 (0.107, 0.160) | 0.669 (0.481, 0.930) |
| Sapovirus | 0.185 (0.134, 0.237) | 0.100 (0.079, 0.121) | 0.528 (0.369, 0.754) |

Prevalence ratios and 95% confidence intervals for the effects of any WASH or Nutrition intervention on enteric pathogen carriage. Enteric pathogen carriage was measured via qPCR. Only pathogens with over 5% prevalence in the study sample were included.

# Table S11: Mediator-Outcome Effects, Pooled Intervention Group and Enteric Pathogen Etiology

| **Outcome** | **Mediator** | **Prevalence/Mean in Absence of Mediator** | **Prevalence/Mean in Presence of Mediator** | **Mean Difference (95% CI)** | **Prevalence Ratio (95% CI)** |
| --- | --- | --- | --- | --- | --- |
| Any Antibiotics  (Past Month) | ***BACTERIA*** | | | | |
|  | Aeromonas | 0.554 (0.528, 0.581) | 0.635 (0.538, 0.733) | ---- | 1.187 (1.003, 1.405) |
|  | B.fragilis | 0.557 (0.527, 0.586) | 0.569 (0.512, 0.625) | ---- | 1.025 (0.910, 1.155) |
|  | C.difficile | 0.562 (0.533, 0.591) | 0.531 (0.450, 0.612) | ---- | 0.924 (0.774, 1.102) |
|  | Campylobacter | 0.614 (0.577, 0.652) | 0.497 (0.461, 0.533) | ---- | 0.796 (0.714, 0.887) |
|  | EAEC | 0.538 (0.470, 0.607) | 0.566 (0.538, 0.594) | ---- | 1.076 (0.930, 1.244) |
|  | EPEC (Any) | 0.619 (0.582, 0.655) | 0.505 (0.466, 0.545) | ---- | 0.816 (0.732, 0.910) |
|  | ETEC (Any) | 0.577 (0.546, 0.608) | 0.532 (0.498, 0.566) | ---- | 0.952 (0.874, 1.036) |
|  | Plesiomonas | 0.571 (0.543, 0.600) | 0.493 (0.431, 0.556) | ---- | 0.882 (0.759, 1.025) |
|  | Shigella/EIEC | 0.562 (0.532, 0.592) | 0.545 (0.477, 0.612) | ---- | 0.989 (0.856, 1.142) |
|  | STEC | 0.561 (0.532, 0.589) | 0.547 (0.474, 0.620) | ---- | 0.972 (0.829, 1.140) |
|  | ***PARASITES*** | | | | |
|  | Any Parasite | 0.556 (0.518, 0.594) | 0.566 (0.524, 0.608) | ---- | 0.997 (0.885, 1.123) |
|  | Cryptosporidium | 0.558 (0.528, 0.588) | 0.571 (0.492, 0.649) | ---- | 1.037 (0.881, 1.221) |
|  | E.bieneusi | 0.558 (0.530, 0.587) | 0.569 (0.485, 0.654) | ---- | 1.012 (0.857, 1.196) |
|  | Giardia | 0.565 (0.535, 0.595) | 0.524 (0.441, 0.606) | ---- | 0.868 (0.720, 1.048) |
|  | ***VIRUSES*** | | | | |
|  | Any Virus | 0.528 (0.494, 0.562) | 0.618 (0.578, 0.658) | ---- | 1.153 (1.046, 1.270) |
|  | Adenovirus 40/41 | 0.559 (0.531, 0.587) | 0.569 (0.476, 0.661) | ---- | 1.026 (0.851, 1.237) |
|  | Norovirus GII | 0.551 (0.522, 0.580) | 0.609 (0.542, 0.676) | ---- | 1.067 (0.932, 1.222) |
|  | Sapovirus | 0.552 (0.524, 0.580) | 0.613 (0.551, 0.675) | ---- | 1.106 (0.981, 1.248) |
| Any Antibiotics (Past 3 Months) | ***BACTERIA*** | | | | |
|  | Aeromonas | 0.717 (0.693, 0.740) | 0.800 (0.716, 0.884) | ---- | 1.143 (1.032, 1.267) |
|  | B.fragilis | 0.726 (0.697, 0.755) | 0.707 (0.656, 0.758) | ---- | 0.969 (0.883, 1.063) |
|  | C.difficile | 0.721 (0.696, 0.747) | 0.723 (0.660, 0.786) | ---- | 1.008 (0.915, 1.111) |
|  | Campylobacter | 0.759 (0.728, 0.790) | 0.679 (0.647, 0.711) | ---- | 0.887 (0.835, 0.942) |
|  | EAEC | 0.711 (0.652, 0.770) | 0.725 (0.701, 0.749) | ---- | 1.016 (0.932, 1.109) |
|  | EPEC (Any) | 0.754 (0.719, 0.788) | 0.692 (0.659, 0.725) | ---- | 0.921 (0.861, 0.985) |
|  | ETEC (Any) | 0.734 (0.709, 0.759) | 0.703 (0.667, 0.738) | ---- | 0.966 (0.910, 1.026) |
|  | Plesiomonas | 0.722 (0.698, 0.746) | 0.719 (0.649, 0.789) | ---- | 1.014 (0.909, 1.131) |
|  | Shigella/EIEC | 0.729 (0.701, 0.756) | 0.678 (0.611, 0.745) | ---- | 0.924 (0.821, 1.041) |
|  | STEC | 0.725 (0.699, 0.750) | 0.698 (0.627, 0.769) | ---- | 0.974 (0.872, 1.087) |
|  | ***PARASITES*** | | | | |
|  | Any Parasite | 0.721 (0.688, 0.753) | 0.723 (0.686, 0.760) | ---- | 0.996 (0.924, 1.073) |
|  | Cryptosporidium | 0.722 (0.696, 0.748) | 0.718 (0.651, 0.784) | ---- | 1.002 (0.895, 1.122) |
|  | E.bieneusi | 0.724 (0.698, 0.750) | 0.701 (0.624, 0.779) | ---- | 0.976 (0.863, 1.104) |
|  | Giardia | 0.726 (0.699, 0.752) | 0.696 (0.629, 0.764) | ---- | 0.943 (0.844, 1.054) |
|  | ***VIRUSES*** | | | | |
|  | Any Virus | 0.705 (0.672, 0.738) | 0.753 (0.714, 0.791) | ---- | 1.060 (0.985, 1.142) |
|  | Adenovirus 40/41 | 0.721 (0.695, 0.748) | 0.725 (0.660, 0.789) | ---- | 1.016 (0.914, 1.129) |
|  | Norovirus GII | 0.719 (0.693, 0.746) | 0.734 (0.680, 0.789) | ---- | 1.005 (0.918, 1.101) |
|  | Sapovirus | 0.718 (0.692, 0.743) | 0.751 (0.688, 0.815) | ---- | 1.056 (0.963, 1.160) |
| Days of Antibiotic Use (Past 3 Months) | ***BACTERIA*** | | | | |
|  | Aeromonas | 5.649 (5.350, 5.949) | 6.082 (5.001, 7.164) | 0.583 (-0.539, 1.705) | ---- |
|  | B.fragilis | 5.689 (5.362, 6.017) | 5.628 (5.081, 6.176) | 0.033 (-0.641, 0.707) | ---- |
|  | C.difficile | 5.659 (5.351, 5.967) | 5.838 (4.837, 6.840) | 0.187 (-0.901, 1.275) | ---- |
|  | Campylobacter | 6.283 (5.890, 6.677) | 4.989 (4.644, 5.335) | -1.098 (-1.639, -0.557) | ---- |
|  | EAEC | 5.464 (4.879, 6.050) | 5.742 (5.413, 6.072) | 0.455 (-0.231, 1.142) | ---- |
|  | EPEC (Any) | 6.067 (5.610, 6.524) | 5.320 (4.955, 5.686) | -0.753 (-1.362, -0.144) | ---- |
|  | ETEC (Any) | 5.841 (5.488, 6.193) | 5.425 (4.997, 5.853) | -0.098 (-0.747, 0.552) | ---- |
|  | Plesiomonas | 5.736 (5.389, 6.082) | 5.346 (4.678, 6.014) | -0.003 (-0.868, 0.862) | ---- |
|  | Shigella/EIEC | 5.828 (5.512, 6.144) | 4.767 (4.170, 5.365) | -0.788 (-1.567, -0.009) | ---- |
|  | STEC | 5.674 (5.366, 5.981) | 5.690 (4.797, 6.583) | 0.072 (-0.902, 1.046) | ---- |
|  | ***PARASITES*** | | | | |
|  | Any Parasite | 5.560 (5.205, 5.915) | 5.889 (5.402, 6.375) | 0.352 (-0.270, 0.974) | ---- |
|  | Cryptosporidium | 5.698 (5.364, 6.032) | 5.512 (4.587, 6.437) | -0.268 (-1.282, 0.745) | ---- |
|  | E.bieneusi | 5.584 (5.285, 5.882) | 6.479 (5.490, 7.468) | 0.885 (-0.223, 1.993) | ---- |
|  | Giardia | 5.691 (5.379, 6.003) | 5.576 (4.738, 6.414) | -0.328 (-1.291, 0.636) | ---- |
|  | ***VIRUSES*** | | | | |
|  | Any Virus | 5.606 (5.260, 5.953) | 5.804 (5.276, 6.333) | 0.212 (-0.400, 0.824) | ---- |
|  | Adenovirus 40/41 | 5.695 (5.392, 5.998) | 5.444 (4.518, 6.370) | -0.106 (-1.169, 0.958) | ---- |
|  | Norovirus GII | 5.666 (5.370, 5.963) | 5.728 (4.937, 6.519) | -0.061 (-0.908, 0.786) | ---- |
|  | Sapovirus | 5.677 (5.364, 5.990) | 5.663 (4.763, 6.562) | 0.166 (-0.821, 1.153) | ---- |
| Episodes of Antibiotic Use (Past 3 Months) | ***BACTERIA*** | | | | |
|  | Aeromonas | 1.106 (1.056, 1.155) | 1.259 (1.067, 1.451) | 0.183 (-0.011, 0.377) | ---- |
|  | B.fragilis | 1.119 (1.062, 1.176) | 1.103 (1.000, 1.206) | 0.000 (-0.120, 0.120) | ---- |
|  | C.difficile | 1.117 (1.064, 1.170) | 1.100 (0.940, 1.260) | -0.003 (-0.180, 0.175) | ---- |
|  | Campylobacter | 1.226 (1.165, 1.287) | 0.989 (0.920, 1.059) | -0.221 (-0.312, -0.129) | ---- |
|  | EAEC | 1.074 (0.970, 1.177) | 1.128 (1.074, 1.182) | 0.082 (-0.033, 0.197) | ---- |
|  | EPEC (Any) | 1.206 (1.124, 1.288) | 1.033 (0.967, 1.098) | -0.166 (-0.275, -0.056) | ---- |
|  | ETEC (Any) | 1.131 (1.072, 1.189) | 1.091 (1.018, 1.165) | -0.010 (-0.112, 0.092) | ---- |
|  | Plesiomonas | 1.126 (1.071, 1.181) | 1.055 (0.932, 1.178) | -0.026 (-0.169, 0.118) | ---- |
|  | Shigella/EIEC | 1.134 (1.080, 1.189) | 1.000 (0.884, 1.116) | -0.126 (-0.263, 0.011) | ---- |
|  | STEC | 1.118 (1.066, 1.170) | 1.094 (0.927, 1.262) | -0.043 (-0.218, 0.131) | ---- |
|  | ***PARASITES*** | | | | |
|  | Any Parasite | 1.097 (1.030, 1.165) | 1.147 (1.069, 1.226) | 0.049 (-0.064, 0.163) | ---- |
|  | Cryptosporidium | 1.120 (1.062, 1.178) | 1.076 (0.935, 1.218) | -0.055 (-0.212, 0.103) | ---- |
|  | E.bieneusi | 1.102 (1.051, 1.153) | 1.229 (1.064, 1.395) | 0.119 (-0.060, 0.298) | ---- |
|  | Giardia | 1.121 (1.065, 1.177) | 1.079 (0.942, 1.215) | -0.054 (-0.216, 0.109) | ---- |
|  | ***VIRUSES*** | | | | |
|  | Any Virus | 1.085 (1.021, 1.149) | 1.170 (1.083, 1.257) | 0.074 (-0.032, 0.179) | ---- |
|  | Adenovirus 40/41 | 1.109 (1.058, 1.160) | 1.183 (0.991, 1.376) | 0.087 (-0.125, 0.299) | ---- |
|  | Norovirus GII | 1.114 (1.061, 1.167) | 1.121 (1.000, 1.241) | -0.022 (-0.162, 0.117) | ---- |
|  | Sapovirus | 1.111 (1.056, 1.167) | 1.142 (1.010, 1.274) | 0.042 (-0.106, 0.191) | ---- |
| Multiple Episodes of Antibiotic Use (Past 3 Months) | ***BACTERIA*** | | | | |
|  | Aeromonas | 0.281 (0.255, 0.307) | 0.329 (0.240, 0.418) | ---- | 1.214 (0.900, 1.637) |
|  | B.fragilis | 0.284 (0.258, 0.311) | 0.283 (0.230, 0.337) | ---- | 1.064 (0.875, 1.294) |
|  | C.difficile | 0.285 (0.258, 0.311) | 0.277 (0.195, 0.359) | ---- | 0.986 (0.724, 1.344) |
|  | Campylobacter | 0.333 (0.304, 0.363) | 0.228 (0.194, 0.263) | ---- | 0.711 (0.601, 0.841) |
|  | EAEC | 0.251 (0.202, 0.300) | 0.295 (0.266, 0.323) | ---- | 1.242 (0.986, 1.564) |
|  | EPEC (Any) | 0.321 (0.278, 0.364) | 0.251 (0.219, 0.282) | ---- | 0.805 (0.665, 0.975) |
|  | ETEC (Any) | 0.299 (0.268, 0.330) | 0.262 (0.228, 0.295) | ---- | 0.930 (0.781, 1.109) |
|  | Plesiomonas | 0.294 (0.266, 0.322) | 0.230 (0.179, 0.282) | ---- | 0.831 (0.646, 1.068) |
|  | Shigella/EIEC | 0.291 (0.264, 0.318) | 0.243 (0.187, 0.298) | ---- | 0.829 (0.648, 1.062) |
|  | STEC | 0.283 (0.257, 0.310) | 0.289 (0.214, 0.365) | ---- | 0.979 (0.743, 1.290) |
|  | ***PARASITES*** | | | | |
|  | Any Parasite | 0.283 (0.251, 0.314) | 0.287 (0.248, 0.325) | ---- | 1.065 (0.887, 1.281) |
|  | Cryptosporidium | 0.288 (0.260, 0.316) | 0.259 (0.194, 0.324) | ---- | 0.878 (0.652, 1.183) |
|  | E.bieneusi | 0.278 (0.254, 0.303) | 0.333 (0.257, 0.410) | ---- | 1.217 (0.964, 1.537) |
|  | Giardia | 0.290 (0.263, 0.317) | 0.246 (0.189, 0.303) | ---- | 0.882 (0.683, 1.140) |
|  | ***VIRUSES*** | | | | |
|  | Any Virus | 0.271 (0.242, 0.300) | 0.308 (0.264, 0.353) | ---- | 1.090 (0.921, 1.289) |
|  | Adenovirus 40/41 | 0.282 (0.257, 0.306) | 0.312 (0.202, 0.422) | ---- | 1.131 (0.783, 1.634) |
|  | Norovirus GII | 0.282 (0.257, 0.308) | 0.295 (0.229, 0.360) | ---- | 0.973 (0.769, 1.231) |
|  | Sapovirus | 0.282 (0.255, 0.309) | 0.302 (0.232, 0.371) | ---- | 1.068 (0.830, 1.373) |

Point estimates and 95% confidence intervals for the effects of enteric pathogen carriage on antibiotic use. For categorical outcomes (any antibiotic use, multiple episodes of antibiotic use), the prevalence ratio is reported. For continuous outcomes (days of antibiotic use, episodes of antibiotic use), the mean difference is reported. Enteric pathogen carriage was measured via qPCR. Only pathogens with over 5% prevalence in the study sample were included.

# Table S12: Mediated Effects, Pooled Intervention Group and Enteric Pathogen Carriage

| **Outcome** | **Mediator** | **Prevalence Difference (95% CI)** |
| --- | --- | --- |
| Any Antibiotics  (Past Month) | ***BACTERIA*** | |
|  | Aeromonas | -0.003 (-0.011, 0.001) |
|  | B.fragilis | 0.001 (-0.003, 0.006) |
|  | C.difficile | 0.000 (-0.003, 0.003) |
|  | Campylobacter | -0.003 (-0.014, 0.005) |
|  | EAEC | -0.001 (-0.010, 0.004) |
|  | EPEC (Any) | 0.004 (-0.005, 0.015) |
|  | ETEC (Any) | 0.001 (-0.002, 0.004) |
|  | Plesiomonas | 0.001 (-0.003, 0.006) |
|  | Shigella/EIEC | 0.000 (-0.003, 0.002) |
|  | STEC | 0.000 (-0.002, 0.003) |
|  | ***PARASITES*** | |
|  | Any Parasite | 0.000 (-0.003, 0.005) |
|  | Cryptosporidium | 0.001 (-0.003, 0.007) |
|  | E.bieneusi | 0.000 (-0.003, 0.002) |
|  | Giardia | 0.000 (-0.005, 0.004) |
|  | ***VIRUSES*** | |
|  | Any Virus | -0.015 (-0.032, -0.004) |
|  | Adenovirus 40/41 | -0.001 (-0.010, 0.006) |
|  | Norovirus GII | -0.002 (-0.008, 0.002) |
|  | Sapovirus | -0.004 (-0.012, 0.001) |
| Any Antibiotics (Past 3 Months) | ***BACTERIA*** | |
|  | Aeromonas | -0.004 (-0.012, 0.000) |
|  | B.fragilis | -0.001 (-0.006, 0.003) |
|  | C.difficile | 0.000 (-0.002, 0.002) |
|  | Campylobacter | -0.002 (-0.009, 0.004) |
|  | EAEC | 0.000 (-0.006, 0.003) |
|  | EPEC (Any) | 0.002 (-0.003, 0.009) |
|  | ETEC (Any) | 0.001 (-0.002, 0.004) |
|  | Plesiomonas | 0.000 (-0.004, 0.003) |
|  | Shigella/EIEC | 0.000 (-0.003, 0.003) |
|  | STEC | 0.000 (-0.002, 0.004) |
|  | ***PARASITES*** | |
|  | Any Parasite | 0.000 (-0.002, 0.004) |
|  | Cryptosporidium | 0.000 (-0.002, 0.004) |
|  | E.bieneusi | 0.000 (-0.002, 0.003) |
|  | Giardia | 0.000 (-0.003, 0.003) |
|  | ***VIRUSES*** | |
|  | Any Virus | -0.007 (-0.019, 0.002) |
|  | Adenovirus 40/41 | 0.000 (-0.007, 0.006) |
|  | Norovirus GII | 0.000 (-0.004, 0.003) |
|  | Sapovirus | -0.002 (-0.009, 0.002) |
| Days of Antibiotic Use (Past 3 Months) | ***BACTERIA*** | |
|  | Aeromonas | -0.019 (-0.093, 0.025) |
|  | B.fragilis | -0.001 (-0.037, 0.039) |
|  | C.difficile | 0.002 (-0.032, 0.039) |
|  | Campylobacter | -0.024 (-0.126, 0.088) |
|  | EAEC | -0.009 (-0.074, 0.039) |
|  | EPEC (Any) | 0.031 (-0.040, 0.122) |
|  | ETEC (Any) | 0.006 (-0.029, 0.061) |
|  | Plesiomonas | 0.002 (-0.034, 0.041) |
|  | Shigella/EIEC | 0.012 (-0.037, 0.075) |
|  | STEC | 0.000 (-0.030, 0.031) |
|  | ***PARASITES*** | |
|  | Any Parasite | 0.007 (-0.044, 0.068) |
|  | Cryptosporidium | 0.001 (-0.044, 0.051) |
|  | E.bieneusi | -0.010 (-0.070, 0.033) |
|  | Giardia | -0.001 (-0.036, 0.032) |
|  | ***VIRUSES*** | |
|  | Any Virus | -0.016 (-0.126, 0.094) |
|  | Adenovirus 40/41 | 0.018 (-0.057, 0.099) |
|  | Norovirus GII | 0.003 (-0.036, 0.048) |
|  | Sapovirus | -0.001 (-0.089, 0.087) |
| Episodes of Antibiotic Use (Past 3 Months) | ***BACTERIA*** | |
|  | Aeromonas | -0.006 (-0.022, 0.002) |
|  | B.fragilis | -0.001 (-0.009, 0.007) |
|  | C.difficile | 0.000 (-0.005, 0.005) |
|  | Campylobacter | -0.006 (-0.025, 0.013) |
|  | EAEC | -0.002 (-0.014, 0.007) |
|  | EPEC (Any) | 0.007 (-0.009, 0.027) |
|  | ETEC (Any) | 0.000 (-0.005, 0.007) |
|  | Plesiomonas | 0.001 (-0.005, 0.007) |
|  | Shigella/EIEC | 0.000 (-0.006, 0.008) |
|  | STEC | 0.000 (-0.005, 0.006) |
|  | ***PARASITES*** | |
|  | Any Parasite | 0.002 (-0.006, 0.011) |
|  | Cryptosporidium | 0.000 (-0.007, 0.007) |
|  | E.bieneusi | 0.000 (-0.008, 0.005) |
|  | Giardia | 0.000 (-0.006, 0.006) |
|  | ***VIRUSES*** | |
|  | Any Virus | -0.008 (-0.030, 0.010) |
|  | Adenovirus 40/41 | -0.004 (-0.026, 0.011) |
|  | Norovirus GII | 0.001 (-0.006, 0.008) |
|  | Sapovirus | 0.001 (-0.010, 0.012) |
| Multiple Episodes of Antibiotic Use (Past 3 Months) | ***BACTERIA*** | |
|  | Aeromonas | -0.002 (-0.008, 0.001) |
|  | B.fragilis | 0.000 (-0.002, 0.004) |
|  | C.difficile | 0.000 (-0.003, 0.003) |
|  | Campylobacter | -0.001 (-0.008, 0.005) |
|  | EAEC | -0.001 (-0.009, 0.005) |
|  | EPEC (Any) | 0.002 (-0.003, 0.009) |
|  | ETEC (Any) | 0.000 (-0.002, 0.004) |
|  | Plesiomonas | 0.001 (-0.002, 0.005) |
|  | Shigella/EIEC | 0.000 (-0.002, 0.002) |
|  | STEC | 0.000 (-0.002, 0.002) |
|  | ***PARASITES*** | |
|  | Any Parasite | 0.000 (-0.003, 0.004) |
|  | Cryptosporidium | 0.000 (-0.003, 0.003) |
|  | E.bieneusi | -0.001 (-0.005, 0.002) |
|  | Giardia | 0.000 (-0.002, 0.003) |
|  | ***VIRUSES*** | |
|  | Any Virus | -0.004 (-0.015, 0.004) |
|  | Adenovirus 40/41 | -0.002 (-0.013, 0.005) |
|  | Norovirus GII | 0.000 (-0.003, 0.003) |
|  | Sapovirus | -0.001 (-0.008, 0.004) |

Point estimates and 95% confidence intervals for the total natural indirect effects of any WASH or Nutrition intervention on antibiotic use. Enteric pathogen carriage was measured via qPCR. Only pathogens with over 5% prevalence in the study sample were included.

# Table S13: Intervention-Mediator Effects, for Enteric Viruses with Pathogen Loads Reflecting Diarrheal Etiology

| **Intervention Group** | **Mediator** | **Prevalence/Mean in Control Arm** | **Prevalence/Mean in Intervention Arm** | **Prevalence Ratio (95% CI)** |
| --- | --- | --- | --- | --- |
| Pooled Intervention | Any Enteric Virus | 0.751 (0.698, 0.804) | 0.746 (0.713, 0.779) | 0.954 (0.882, 1.031) |
|  | Adenovirus 40/41 | 0.100 (0.049, 0.152) | 0.067 (0.045, 0.088) | 0.587 (0.355, 0.970) |
|  | Norovirus GII | 0.164 (0.119, 0.209) | 0.098 (0.077, 0.120) | 0.578 (0.397, 0.841) |
|  | Sapovirus | 0.158 (0.112, 0.204) | 0.077 (0.059, 0.095) | 0.473 (0.316, 0.709) |
| Pooled Nutrition and Nutrition + WASH | Any Enteric Virus | 0.751 (0.698, 0.804) | 0.755 (0.719, 0.791) | 0.961 (0.887, 1.042) |
|  | Adenovirus 40/41 | 0.100 (0.049, 0.152) | 0.073 (0.045, 0.102) | 0.654 (0.391, 1.093) |
|  | Norovirus GII | 0.164 (0.119, 0.209) | 0.120 (0.090, 0.149) | 0.718 (0.487, 1.058) |
|  | Sapovirus | 0.158 (0.112, 0.204) | 0.083 (0.061, 0.105) | 0.516 (0.331, 0.802) |
| Pooled WASH and Nutrition + WASH | Any Enteric Virus | 0.751 (0.698, 0.804) | 0.740 (0.704, 0.776) | 0.954 (0.875, 1.040) |
|  | Adenovirus 40/41 | 0.100 (0.049, 0.152) | 0.061 (0.039, 0.082) | 0.502 (0.280, 0.898) |
|  | Norovirus GII | 0.164 (0.119, 0.209) | 0.088 (0.066, 0.110) | 0.519 (0.343, 0.786) |
|  | Sapovirus | 0.158 (0.112, 0.204) | 0.077 (0.056, 0.098) | 0.480 (0.315, 0.731) |
| Nutrition | Any Enteric Virus | 0.751 (0.698, 0.804) | 0.759 (0.706, 0.811) | 0.957 (0.873, 1.049) |
|  | Adenovirus 40/41 | 0.100 (0.049, 0.152) | 0.080 (0.041, 0.118) | 0.721 (0.404, 1.288) |
|  | Norovirus GII | 0.164 (0.119, 0.209) | 0.119 (0.074, 0.164) | 0.705 (0.441, 1.129) |
|  | Sapovirus | 0.158 (0.112, 0.204) | 0.077 (0.044, 0.109) | 0.441 (0.246, 0.792) |
| WASH | Any Enteric Virus | 0.751 (0.698, 0.804) | 0.728 (0.678, 0.779) | 0.938 (0.848, 1.037) |
|  | Adenovirus 40/41 | 0.100 (0.049, 0.152) | 0.054 (0.029, 0.079) | 0.437 (0.217, 0.878) |
|  | Norovirus GII | 0.164 (0.119, 0.209) | 0.057 (0.033, 0.081) | 0.321 (0.190, 0.544) |
|  | Sapovirus | 0.158 (0.112, 0.204) | 0.065 (0.034, 0.096) | 0.376 (0.209, 0.677) |
| Nutrition + WASH | Any Enteric Virus | 0.751 (0.698, 0.804) | 0.752 (0.703, 0.802) | 0.967 (0.875, 1.068) |
|  | Adenovirus 40/41 | 0.100 (0.049, 0.152) | 0.067 (0.036, 0.098) | 0.561 (0.299, 1.055) |
|  | Norovirus GII | 0.164 (0.119, 0.209) | 0.120 (0.081, 0.159) | 0.727 (0.456, 1.159) |
|  | Sapovirus | 0.158 (0.112, 0.204) | 0.089 (0.059, 0.120) | 0.567 (0.348, 0.923) |

Prevalance ratio estimates and 95% confidence intervals for the effects of WASH and/or Nutrition interventions on enteric virus carriage at 14 months with pathogen loads that reflect diarrheal etiology based on published Ct cutoff values from the MAL-ED study. Any Enteric Virus is the presence of Adenovirus 40/41, Norovirus GII, or Sapovirus that exceeded the etiology cutoff.

# Table S14: Mediator-Outcome Effects, for Enteric Viruses with Pathogen Loads Reflecting Diarrheal Etiology

| **Mediator** | **Outcome** | **Prevalence/Mean in Absence of Mediator** | **Prevalence/Mean in Presence of Mediator** | **Mean Difference (95% CI)** | **Prevalence Ratio (95% CI)** |
| --- | --- | --- | --- | --- | --- |
| Any Enteric Virus | Any antibiotic use in past month | 0.590 (0.539, 0.641) | 0.549 (0.517, 0.581) | ---- | 0.958 (0.850, 1.079) |
|  | Any antibiotic use in past 3 months | 0.736 (0.687, 0.785) | 0.717 (0.688, 0.745) | ---- | 0.999 (0.916, 1.089) |
|  | Days of antibiotic use in past 3 months | 5.738 (5.241, 6.236) | 5.654 (5.327, 5.982) | 0.158 (-0.507, 0.824) | ---- |
|  | Episodes of antibiotic use in past 3 months | 1.163 (1.070, 1.256) | 1.099 (1.042, 1.156) | -0.024 (-0.142, 0.093) | ---- |
|  | Multiple episodes of antibiotic use | 0.298 (0.252, 0.344) | 0.279 (0.252, 0.307) | ---- | 0.995 (0.823, 1.202) |
| Adenovirus 40/41 | Any antibiotic use in past month | 0.560 (0.532, 0.588) | 0.552 (0.455, 0.650) | ---- | 0.992 (0.815, 1.207) |
|  | Any antibiotic use in past 3 months | 0.722 (0.696, 0.748) | 0.714 (0.646, 0.783) | ---- | 0.999 (0.895, 1.114) |
|  | Days of antibiotic use in past 3 months | 5.704 (5.401, 6.007) | 5.317 (4.418, 6.217) | -0.268 (-1.285, 0.749) | ---- |
|  | Episodes of antibiotic use in past 3 months | 1.110 (1.059, 1.161) | 1.181 (0.989, 1.373) | 0.082 (-0.129, 0.293) | ---- |
|  | Multiple episodes of antibiotic use | 0.282 (0.257, 0.306) | 0.314 (0.210, 0.419) | ---- | 1.137 (0.804, 1.607) |
| Norovirus GII | Any antibiotic use in past month | 0.550 (0.523, 0.577) | 0.631 (0.560, 0.702) | ---- | 1.107 (0.967, 1.268) |
|  | Any antibiotic use in past 3 months | 0.721 (0.696, 0.747) | 0.725 (0.664, 0.786) | ---- | 0.991 (0.900, 1.091) |
|  | Days of antibiotic use in past 3 months | 5.667 (5.371, 5.962) | 5.742 (4.829, 6.656) | -0.129 (-1.096, 0.837) | ---- |
|  | Episodes of antibiotic use in past 3 months | 1.113 (1.061, 1.164) | 1.131 (0.995, 1.267) | -0.013 (-0.167, 0.142) | ---- |
|  | Multiple episodes of antibiotic use | 0.281 (0.256, 0.306) | 0.306 (0.235, 0.378) | ---- | 1.011 (0.792, 1.289) |
| Sapovirus | Any antibiotic use in past month | 0.552 (0.525, 0.580) | 0.627 (0.546, 0.708) | ---- | 1.139 (0.994, 1.304) |
|  | Any antibiotic use in past 3 months | 0.719 (0.694, 0.744) | 0.748 (0.667, 0.830) | ---- | 1.061 (0.950, 1.185) |
|  | Days of antibiotic use in past 3 months | 5.648 (5.344, 5.953) | 5.933 (4.792, 7.074) | 0.480 (-0.724, 1.684) | ---- |
|  | Episodes of antibiotic use in past 3 months | 1.108 (1.053, 1.163) | 1.185 (1.014, 1.356) | 0.094 (-0.091, 0.278) | ---- |
|  | Multiple episodes of antibiotic use | 0.279 (0.252, 0.306) | 0.333 (0.246, 0.421) | ---- | 1.177 (0.876, 1.581) |

Point estimates and 95% confidence intervals for the effect of enteric virus carriage at 14 months with pathogen loads that reflect diarrheal etiology. For categorical mediators, the prevalence ratio is reported. For continuous mediators, the mean difference is reported. Diarrheal etiology is assessed using published Ct cutoff values from the MAL-ED study. Any Enteric Virus is the presence of Adenovirus 40/41, Norovirus GII, or Sapovirus that exceeded the etiology cutoff. Antibiotic use is reported by a caregiver under either a 1- or 3-month lookback period at 14 and 28 months.

# Table S15: Mediated Effects, for Enteric Viruses with Pathogen Loads Reflecting Diarrheal Etiology

| **Intervention Group** | **Mediator** | **Outcome** | **Total Natural Indirect Effect (Prevalence Difference)** | **Total Natural Indirect Effect (Prevalence Ratio)** |
| --- | --- | --- | --- | --- |
| Pooled Intervention | Adenovirus 40/41 (Symptomatic) | Multiple episodes of antibiotic use in past 3 months | -0.002 (-0.012, 0.004) | 0.997 (0.976, 1.025) |
|  | Norovirus GII (Symptomatic) | Any antibiotic use in past month | -0.003 (-0.011, 0.001) | 0.997 (0.988, 1.004) |
|  | Sapovirus (Symptomatic) | Any antibiotic use in past month | -0.004 (-0.013, 0.001) | 0.995 (0.985, 1.002) |
|  | Sapovirus (Symptomatic) | Multiple episodes of antibiotic use in past 3 months | -0.003 (-0.011, 0.003) | 0.995 (0.978, 1.013) |
| Pooled Nutrition and Nutrition + WASH | Adenovirus 40/41 (Symptomatic) | Multiple episodes of antibiotic use in past 3 months | 0.000 (-0.008, 0.007) | 1.005 (0.983, 1.043) |
|  | Norovirus GII (Symptomatic) | Any antibiotic use in past month | -0.001 (-0.007, 0.003) | 1.000 (0.993, 1.008) |
|  | Sapovirus (Symptomatic) | Any antibiotic use in past month | -0.003 (-0.012, 0.002) | 0.996 (0.985, 1.005) |
|  | Sapovirus (Symptomatic) | Multiple episodes of antibiotic use in past 3 months | -0.002 (-0.012, 0.003) | 0.998 (0.981, 1.017) |
| Pooled WASH and Nutrition + WASH | Adenovirus 40/41 (Symptomatic) | Multiple episodes of antibiotic use in past 3 months | -0.003 (-0.021, 0.008) | 0.997 (0.966, 1.042) |
|  | Norovirus GII (Symptomatic) | Any antibiotic use in past month | -0.004 (-0.012, 0.001) | 0.996 (0.984, 1.004) |
|  | Sapovirus (Symptomatic) | Any antibiotic use in past month | -0.006 (-0.016, 0.000) | 0.994 (0.982, 1.004) |
|  | Sapovirus (Symptomatic) | Multiple episodes of antibiotic use in past 3 months | -0.005 (-0.016, 0.001) | 0.994 (0.975, 1.012) |
| Nutrition | Adenovirus 40/41 (Symptomatic) | Multiple episodes of antibiotic use in past 3 months | -0.001 (-0.010, 0.004) | 1.001 (0.979, 1.034) |
|  | Norovirus GII (Symptomatic) | Any antibiotic use in past month | -0.001 (-0.008, 0.004) | 1.000 (0.991, 1.009) |
|  | Sapovirus (Symptomatic) | Any antibiotic use in past month | -0.004 (-0.017, 0.004) | 0.996 (0.982, 1.012) |
|  | Sapovirus (Symptomatic) | Multiple episodes of antibiotic use in past 3 months | -0.003 (-0.015, 0.004) | 0.996 (0.970, 1.025) |
| WASH | Adenovirus 40/41 (Symptomatic) | Multiple episodes of antibiotic use in past 3 months | -0.005 (-0.019, 0.004) | 0.992 (0.966, 1.017) |
|  | Norovirus GII (Symptomatic) | Any antibiotic use in past month | -0.005 (-0.018, 0.006) | 0.994 (0.974, 1.013) |
|  | Sapovirus (Symptomatic) | Any antibiotic use in past month | -0.009 (-0.027, 0.002) | 0.988 (0.970, 1.002) |
|  | Sapovirus (Symptomatic) | Multiple episodes of antibiotic use in past 3 months | -0.009 (-0.028, 0.003) | 0.985 (0.954, 1.014) |
| Nutrition + WASH | Adenovirus 40/41 (Symptomatic) | Multiple episodes of antibiotic use in past 3 months | 0.001 (-0.016, 0.013) | 1.013 (0.970, 1.103) |
|  | Norovirus GII (Symptomatic) | Any antibiotic use in past month | -0.001 (-0.010, 0.005) | 1.000 (0.992, 1.012) |
|  | Sapovirus (Symptomatic) | Any antibiotic use in past month | -0.004 (-0.019, 0.003) | 0.997 (0.982, 1.011) |
|  | Sapovirus (Symptomatic) | Multiple episodes of antibiotic use in past 3 months | -0.005 (-0.018, 0.003) | 0.998 (0.974, 1.029) |

Point estimates and 95% confidence intervals for the total natural indirect effects of WASH and/or Nutrition intervention on antibiotic use through pathways involving enteric virus carriage at 14 months with pathogen loads that reflect diarrheal etiology. Diarrheal etiology is assessed using published Ct cutoff values from the MAL-ED study. Any Enteric Virus is the presence of Adenovirus 40/41, Norovirus GII, or Sapovirus that exceeded the etiology cutoff. Antibiotic use is reported by a caregiver under either a 1- or 3-month lookback period at 14 and 28 months.

# Table S16: Mediated Effects, Negative Control

| **Intervention Group** | **Mediator** | **Outcome** | **Total Natural Indirect Effect (Prevalence Difference)** |
| --- | --- | --- | --- |
| Pooled Intervention | Bruising (Past 7 Days) | Any antibiotic use in past month | -0.001 (-0.004, 0.001) |
|  |  | Any antibiotic use in past 3 months | -0.002 (-0.007, 0.001) |
|  |  | Days of antibiotic use in past 3 months | -0.006 (-0.033, 0.016) |
|  |  | Episodes of antibiotic use in past 3 months | -0.001 (-0.006, 0.003) |
|  |  | Multiple episodes of antibiotic use | 0.000 (-0.001, 0.002) |
| Pooled Nutrition and Nutrition + WASH | Bruising (Past 7 Days) | Any antibiotic use in past month | 0.000 (-0.002, 0.002) |
|  |  | Any antibiotic use in past 3 months | -0.001 (-0.005, 0.002) |
|  |  | Days of antibiotic use in past 3 months | -0.006 (-0.058, 0.028) |
|  |  | Episodes of antibiotic use in past 3 months | 0.000 (-0.005, 0.004) |
|  |  | Multiple episodes of antibiotic use | 0.000 (-0.002, 0.002) |
| Pooled WASH and  Nutrition + WASH | Bruising (Past 7 Days) | Any antibiotic use in past month | -0.001 (-0.006, 0.002) |
|  |  | Any antibiotic use in past 3 months | -0.002 (-0.007, 0.001) |
|  |  | Days of antibiotic use in past 3 months | -0.016 (-0.063, 0.016) |
|  |  | Episodes of antibiotic use in past 3 months | 0.000 (-0.005, 0.006) |
|  |  | Multiple episodes of antibiotic use | 0.001 (-0.001, 0.004) |
| Nutrition | Bruising (Past 7 Days) | Any antibiotic use in past month | 0.000 (-0.003, 0.004) |
|  |  | Any antibiotic use in past 3 months | -0.001 (-0.007, 0.003) |
|  |  | Days of antibiotic use in past 3 months | -0.009 (-0.084, 0.056) |
|  |  | Episodes of antibiotic use in past 3 months | 0.000 (-0.009, 0.009) |
|  |  | Multiple episodes of antibiotic use | 0.000 (-0.003, 0.003) |
| WASH | Bruising (Past 7 Days) | Any antibiotic use in past month | -0.004 (-0.011, 0.001) |
|  |  | Any antibiotic use in past 3 months | -0.003 (-0.010, 0.001) |
|  |  | Days of antibiotic use in past 3 months | -0.027 (-0.096, 0.016) |
|  |  | Episodes of antibiotic use in past 3 months | -0.001 (-0.011, 0.006) |
|  |  | Multiple episodes of antibiotic use | 0.001 (-0.001, 0.005) |
| Nutrition + WASH | Bruising (Past 7 Days) | Any antibiotic use in past month | 0.000 (-0.003, 0.003) |
|  |  | Any antibiotic use in past 3 months | -0.001 (-0.006, 0.002) |
|  |  | Days of antibiotic use in past 3 months | -0.014 (-0.083, 0.034) |
|  |  | Episodes of antibiotic use in past 3 months | 0.000 (-0.006, 0.006) |
|  |  | Multiple episodes of antibiotic use | 0.000 (-0.003, 0.003) |

Point estimates and 95% confidence intervals for the total natural indirect effects of WASH and/or Nutrition intervention on antibiotic use through pathways involving bruising, a negative control. Bruising is reported by a caregiver under a 7-day lookback period at 14 and 28 months. Antibiotic use is reported by a caregiver under either a 1- or 3-month lookback period at 14 and 28 months.
